# How have media campaigns been used to promote and discourage healthy and unhealthy beverages in the United States? A systematic scoping review to inform future research to reduce sugary beverage health risks

**DOI:** 10.1111/obr.13425

**Published:** 2022-02-09

**Authors:** Vivica I. Kraak, Katherine Consavage Stanley, Paige B. Harrigan, Mi Zhou

**Affiliations:** ^1^ Department of Human Nutrition, Foods, and Exercise Virginia Tech Blacksburg Virginia USA; ^2^ Department of Public Health University of California Merced Merced California USA

**Keywords:** advertising and marketing, healthy beverage behaviors, sugary beverages, typology, U.S. media campaigns

## Abstract

Sugary beverage consumption is associated with many health risks. This study used a proof‐of‐concept media campaign typology to examine U.S. beverage campaigns that promoted healthy beverages and encouraged or discouraged sugary beverages. We used a three‐step systematic scoping review to identify, organize, analyze, and synthesize evidence. Step 1 used Preferred Reporting Items for Systematic Review and Meta‐Analysis Extension for Scoping Reviews (PRISMA‐ScR) guidelines to search four electronic databases and gray literature through 2021. Step 2 categorized relevant media campaigns using a media campaign typology. Step 3 examined campaign evaluation outcomes. We identified 280 campaigns organized into six campaign typology categories. The media landscape was dominated by corporate marketing campaigns for branded sugary beverages (65.8%; *n* = 184) followed by public awareness (9.6%; *n* = 27), public policy (8.2%; *n* = 23), social marketing (7.1%; *n* = 20), corporate social responsibility (5.7%; *n* = 16), and countermarketing (3.6%; *n* = 10) campaigns. Evaluations for 20 unique campaigns implemented over 30 years (1992–2021) across 14 states showed reduced sugary beverage or juice and increased water or low‐fat milk sales and intake. Positive short‐term cognitive and mid–term retail and behavioral changes were reported. There was limited evidence for long‐term policy, social norm, and population health outcomes. Future research is needed to use media campaigns in strategic communications to reduce sugary beverage health risks for Americans.

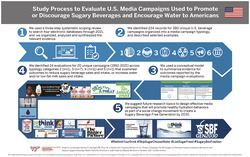

## 1 INTRODUCTION

Human behaviors are influenced by the images, stories, and textual messages communicated through the mass media.^1^ Public health media campaigns use print, broadcast, and social media to influence people's attitudes and behaviors to reduce health risks.^1^ Many corporate‐funded marketing campaigns deliver competing messages that encourage people to buy and consume sugary beverages that contribute to poor diet quality, dental caries, obesity, type 2 diabetes, and cardiovascular disease in the United States (U.S.) and other countries.^2^, ^3^, ^4^, ^5^

Sugary beverage products are the primary source of added sugars in Americans' diets and include carbonated soft drinks (i.e., soda) and sports and energy drinks; sweetened fruit drinks, juices, and nectars; enhanced waters; sweetened teas and coffees; and sweetened cow's milk and plant‐based nondairy milks.^2^, ^3^ Consumption patterns of sugary beverages vary by age, income, race, and ethnicity. On average, half to two thirds of American adults and nearly two thirds of U.S. children consume at least one sugary beverage each day that contributes 130 or more calories to their daily energy intake.^2^, ^3^, ^6^ Neither the World Health Organization nor the U.S. government have issued comprehensive healthy beverage guidelines across the lifespan.^7^

Health‐oriented media campaigns have used print, broadcast, and social media to support policy, systems, and environmental (PSE) strategies to discourage sugary beverages and promote water, milk, or noncaloric beverages.^7^ We developed a proof‐of‐concept media campaign typology published previously that describes mass media campaigns having different goals, objectives, paradigms, and target populations to promote or discourage behaviors.^7^ The campaign typology has six categories including corporate advertising, marketing, or entertainment; corporate social responsibility, public relations, or cause marketing; social marketing; public information, awareness, education, or health promotion; media advocacy or countermarketing; and political or public policy campaigns.^7^

No study has summarized evidence for U.S. nonalcoholic beverage campaigns or described how corporate versus government and civil society‐funded media campaigns have used print, broadcast, and digital or social media to communicate messages to target populations about healthy and unhealthy beverage products in a crowded media message ecosystem.^7^

**FIGURE 1:**
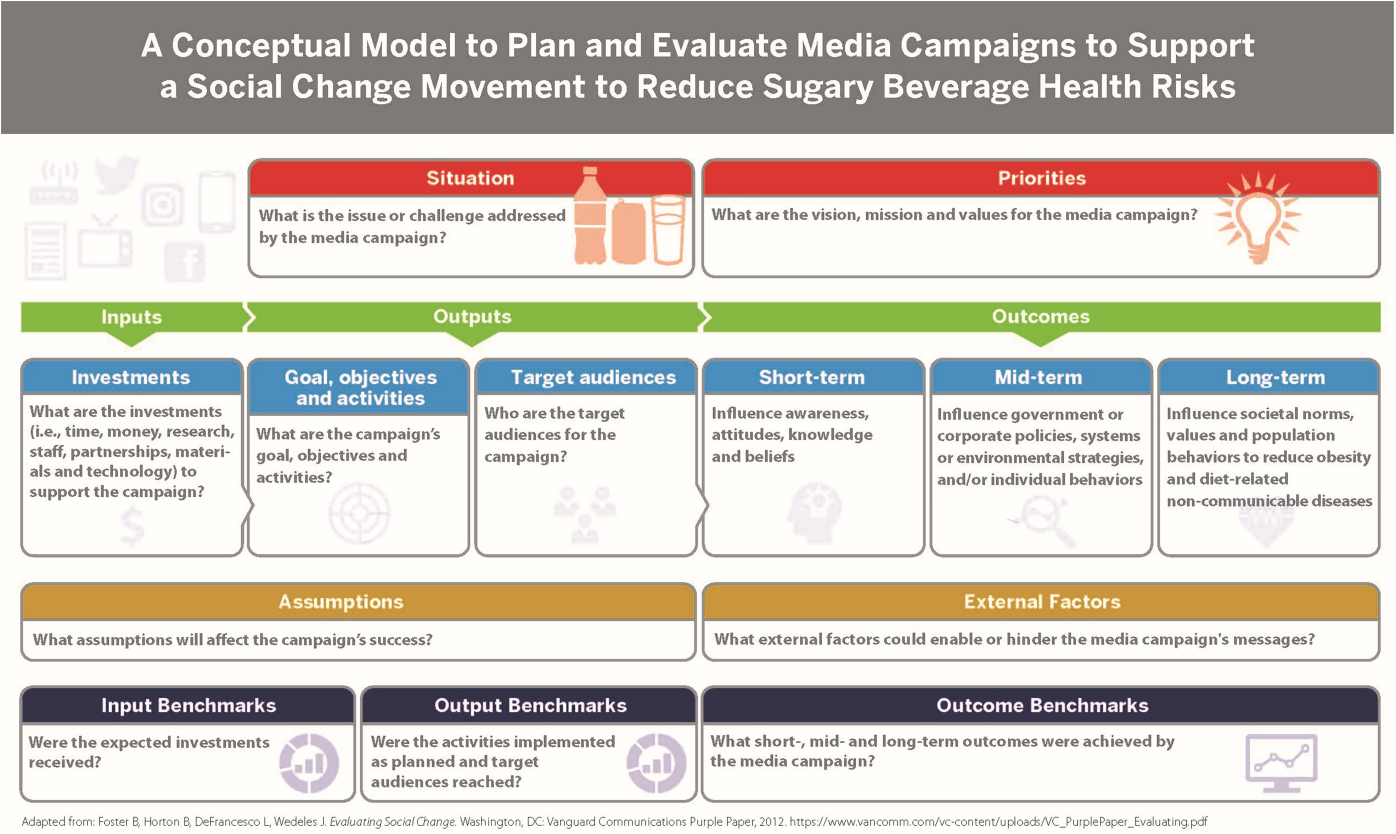
A conceptual model to plan and evaluate media campaigns for a social change movement to reduce sugary beverage health risks

Figure 1 shows a conceptual model to plan and evaluate media campaigns to support a social change movement to reduce sugary beverage health risks for Americans.^7^ In a previous review, we defined social change as a long‐term process that fosters collective action to transform social norms, attitudes, and behaviors of populations for a specific issue over years or decades.^7^ The Tobacco‐Free Generation and Tobacco Endgame concepts^7^, ^8^ have used media to build policy support for a social change movement to de‐normalize and discourage youth from using tobacco and vaping products. These concepts could be adapted to examine evidence for U.S. beverage media campaigns to normalize healthy hydration behaviors and establish a Sugary Beverage‐Free Generation.^7^ This study addresses these research gaps.

### 1.1 Study purpose

This study conducted a systematic scoping review of U.S. beverage media campaigns, categorized evidence into a media campaign typology,^7^ and addressed other research gaps. First, we provide a summary of U.S. beverage recommendations, consumption, and marketing trends. Thereafter, we describe the results from a systematic search of the published and gray literature for U.S. media campaigns used to influence nonalcoholic beverage behaviors, organize the campaigns chronologically by campaign type into the media campaign typology, and synthesize evidence from published media campaign evaluations. We suggest future research needs to build a social change movement to reduce sugary beverage health risks for Americans.

## 2 TRENDS IN U.S. EXPERT BEVERAGE RECOMMENDATIONS, POPULATION INTAKE, AND MARKETING

The 2020 Dietary Guidelines Advisory Committee (DGAC) report noted that sugary beverages contribute about 30%, 50%, and 60% of added sugars to the diets of U.S. children, adolescents, and adults, respectively.^9^ The Dietary Guidelines for Americans (DGA) 2020–2025 report^10^ recommended general guidelines for healthy nonalcoholic beverage intake that encourage (1) water and other beverages that contribute beneficial nutrients, including low‐fat and nonfat milk or a fortified nondairy soy milk and limited amounts of 100% juice; (2) coffee, tea, and flavored water without added sugars or fats; (3) ≤400 mg of caffeine intake per day; (4) <10% of total daily calories from added sugars; and (5) the replacement of sugary beverages with water or other unsweetened beverages.^10^ The DGA 2020–2025 also provided specific healthy beverage targets for infants and toddlers under 2 years of age.^10^ However, the DGA report lacked specific recommendations for daily water intake for children over 2 years old, adolescents, or adults and did not address artificially sweetened or functional beverages.

The U.S. Healthy Eating Research (HER) National Program, supported by the Robert Wood Johnson Foundation (RWJF), convened expert panels that published healthy beverage targets for water, milk, and 100% juice for infants and toddlers, children, teens, and adults in 2013,^11^ and the guidelines for children birth to age 5 years were updated in 2019.^12^ The panel's recommendations were not incorporated into the DGA 2020–2025 for all age groups.^10^

The average U.S. adult woman and man consume about 10 cups (8 oz/240 ml) and 12 cups (8 oz/240 ml) of fluids daily, respectively, which are below the recommended Dietary Reference Intake (DRI) of 11.5–15.5 cups of fluids daily.^13^ Two thirds of U.S. adults consume a sugary beverage daily.^14^ The recommended DRI for children is 4–8 cups of fluids daily.^15^ The average U.S. child aged 2–19 years consumes over 5 cups (8  oz/240 ml) of fluids daily and more than half is from water, followed by sugary beverages and milk.^16^ Fruit drinks, sweetened juices, and flavored or sweetened skim milk are the leading sources of added sugars in young children's diet,^16^ and adolescents overconsume soft drinks, energy drinks, and sports drinks.^2^, ^17^

Nestlé, The Coca‐Cola Company, and PepsiCo Inc. dominate the U.S. and global beverage brand market and sales for sparkling soft drinks and sugary beverages, water, sweetened and unsweetened juices, dairy and plant‐based drinks, coffees, and teas.^18^ These transnational companies have operated in self‐regulated markets where national governments have allowed sugary beverage products to be extensively marketed to drive consumer demand and brand loyalty through integrated marketing communications (IMC) strategies * to encourage sugary beverages linked to obesity and noncommunicable diseases (NCDs).^2^, ^3^, ^4^, ^5^, ^6^, ^7^

Between 2003 and 2016, per capita sugary beverage intake and heavy sugary beverage consumption (≥500 calories daily) declined among U.S. children and adults.^2^, ^3^, ^19^ Sugary beverage intake remained highest among Black, Mexican American, and non‐Mexican Latinx consumers during this period.^2^, ^3^, ^19^ A significant increase in heavy sugary beverage intake was observed among older adults over 60 years^19^ with no change in heavy sugary beverage consumption among adults aged 40–59 years and non‐Mexican Latinx adults (2003–2016).^19^

The U.S. development, marketing, and sales of functional beverages increased between 2013 and 2021.^20^ Functional beverages are a nonalcoholic beverage category that promotes their health‐enhancing benefits attributed to herbs, vitamins, minerals, amino acids, and prebiotics or probiotics. Functional beverages include energy drinks, sports drinks, enhanced waters and juices, and nondairy plant‐based beverages (i.e., almond, cashew, coconut, oat, rice, soy, and blended nut milks)^20^ that may contain excessive added sugars.^2^, ^3^, ^6^

The volume and U.S. per capita purchasing of beverages with nonnutritive sweeteners (sucralose and stevia) and/or caloric sweeteners (sucrose) increased (2002–2018).^21^ A concurrent trend has been the increased sales of bottled water from supermarkets among high‐income populations (2011–2016) and increased intake of both bottled and tap water within the southern and midwestern U.S. states.^22^, ^23^ Only a small proportion of Americans consume the DRI‐recommended daily water intake.^22^

U.S. beverage trends have been driven by the extensive marketing of sugary beverage brands using IMC strategies to build brand loyalty and revenue^17^; permissive government policies that have supported industry self‐regulatory programs^17^; industry marketing of functional beverages with added sugars^20^ that compete with unbranded free tap water; and industry lobbying of legislators that has preempted and opposed state and local laws for pro‐sugary beverage taxes and warning labels.^24^

## 3 METHODOLOGY

### 3.1 Research questions

This study used a systematic scoping review guided by three research questions (RQ).

What specific media campaigns were used to encourage Americans to purchase and/or consume healthy beverages (i.e., water, unflavored low‐fat and fat‐free milk, or 100% juice) recommended by U.S. expert bodies and to promote or discourage unhealthy sugary beverages through 2021?

How are the U.S. beverage campaigns categorized into the media campaign typology?

How can the collective findings from this study inform strategies to promote healthy hydration behaviors and reduce sugary beverage health risks for Americans?

### 3.2 Search strategy, evidence selection, and extraction

We conducted a systematic scoping review due to the exploratory nature of the study. The scoping review used five steps described by Arksey and O'Malley^25^ to identify the research question, identify relevant studies that meet inclusion criteria, select and document the evidence, and summarize the results. We examined the peer‐reviewed literature, gray literature sources, and media or press releases relevant to the research questions within the U.S. context. The search strategy was aligned with RQ1 and RQ2 and was guided by the Preferred Reporting Items for Systematic Review and Meta‐Analysis Protocol (PRISMA‐P)^26^ and PRISMA Extension for Scoping Reviews (PRISMA‐ScR) checklists.^27^ We did not assess the study quality or risk of bias due to the broad study purpose to apply the media campaign typology to the U.S. context only. Beverage media campaigns implemented in other countries were excluded from the review process for this paper.

To address RQ1, a coinvestigator (K. C. S.) worked with two university research librarians to design a comprehensive search strategy to identify media campaigns that promoted unsweetened water, low‐fat and fat‐free milk, coffee, tea, or 100% juice and promoted or discouraged branded, nonalcoholic beverages. We used several published review papers to select the search terms and guide the search strategy for beverage campaigns that included Palmedo et al.,^28^ Freudenberg et al.,^29^ Huang et al.,^30^ and Te et al.^31^ Table 1 summarizes the search strategy, including the search terms and inclusion and exclusion criteria used to identify relevant evidence.

**TABLE 1:** Search strategy for the systematic scoping review to examine U.S. media campaigns used to promote or discourage nonalcoholic beverages to Americans

Search strategy	
Inclusion criteria	Exclusion criteria
Articles or gray literature sources available in the English language that mention one or more specific U.S. media campaign and describe at least one other component of the campaign (e.g., location, length [time frame], developer(s), budget, target audience(s), key message(s), and/or intended outcome(s)) Articles or gray literature sources that describe campaigns that ○ Have a clear name and/or slogan assigned to them (e.g., *Got Milk?* campaign, *Kick the Can* campaign); ○ Promote or discourage specific branded sugary beverage products (i.e., carbonated soft drinks; sports and energy drinks; fruit drinks; and sweetened teas, coffees, and milks) from Nestlé, The Coca‐Cola Company, or PepsiCo, Inc^a^; ○ Promote or discourage the purchase and/or consumption of water, cow's milk, plant‐based milk, or 100% juice, including campaigns for branded water, milk, and juice products; ○ Promote or discourage the purchase and consumption of broad beverage categories (e.g., sugary beverages and milk); and ○ Are community, city‐wide, regional, or national in scope.	Articles or gray literature sources that are not available in the English language Articles or gray literature sources describing media campaigns that ○ Do not promote or discourage sugary beverages, milk, juice, and/or water purchase or consumption (e.g., alcoholic beverage campaigns and Human Immunodeficiency Virus (HIV) awareness campaigns); ○ Promote or discourage a branded sugary beverage product that is not one of Nestle, Coca‐Cola Company, or PepsiCo, Inc's products; ○ Are based in a country other than the United States; ○ Have no name or slogan attached to it (i.e., are “unnamed” or “unbranded”); ○ Are focused at the school level or in one establishment (e.g., a specific hospital or workplace); ○ Promote or discourage breast milk, formula, or other products intended to replace breast milk (e.g., “toddler milks”); and ○ Include changes in beverage behavior as just one component of a multipronged behavior change campaign or intervention (e.g., healthy eating, healthy weight, or obesity prevention campaigns) Articles that mention media campaign(s) but do not describe at least one other component of the campaign Theses, dissertations, patents, and conference or poster abstracts
Search platforms	Search terms
Four electronic databases (i.e., CINAHL, Communication & Mass Media Complete, PubMed, and Web of Science)	(campaign) AND (beverage* OR soda* OR cola OR “energy drink” OR “sports drink” OR Pepsi OR Coke OR “Coca‐Cola” OR Nestlé OR (water AND (consum* OR drink* OR tap)) OR “carbonated water” OR “bottled water” OR juice OR milk OR tea OR coffee) AND (health OR promot* OR advocacy OR policy OR political OR tax OR media OR communication* OR advertis* OR information* OR aware* OR behavior* OR “public relations” OR marketing OR countermarketing OR counter‐marketing OR education* OR entertainment OR advocacy OR advocat*) MeSH terms used where applicable: health; “health promotion”; “mass media”; “social marketing”; “health communication”; “public relations”; beverages; “sugar‐sweetened beverage”; “artificially sweetened beverage”; “carbonated beverages”; “carbonated water”; “drinking water”; “energy drinks”; coffee; milk
Google and Google Scholar	(campaign) AND (beverage* OR soda* OR water OR juice OR milk OR tea OR coffee) AND (health OR promot* OR advoc* OR policy OR politic* OR tax OR media OR communic* OR advertis* OR inform* OR aware* OR behav* OR “public relations” OR market* OR educ*)

^a^ Search was limited to three largest global branded beverage manufacturers given the large number of advertising campaigns for beverages and three manufacturers that collectively spent the most on advertising and marketing for beverage products in the United States and globally.

K. C. S. used PRISMA guidelines to search four electronic databases (i.e., CINAHL, Communication & Mass Media Complete, PubMed, and Web of Science) from journal inception through May 31, 2021, and the first 300 hits within Google Scholar.^32^ The search identified peer‐reviewed articles, gray literature, and media sources that described various media campaigns. Backward searches and additional targeted Google searches were conducted to fill known gaps in the campaign results and to identify evaluations not captured by the scoping review. Finally, we searched the websites of nongovernmental organizations (i.e., Center for Science for the Public Interest [CSPI], Healthy Food America, and Berkeley Media Studies Group) and limited the search for campaigns to three firms with the largest U.S. beverage market share that included The Coca‐Cola Company, PepsiCo, Inc., and Nestlé.

K. C. S. screened the titles and abstracts of each article, gray literature source, and media or press release using Covidence, a web‐based software for systematic reviews. K. C. S. and P. B. H. then screened the full‐text articles to determine whether they qualified for inclusion. The coinvestigators resolved disagreements about article inclusion.

To address RQ2, V. I. K. compiled all included campaigns into an evidence table using the categories of a published media campaign typology,^7^ and K. C. S. organized the campaigns and references chronologically. V. I. K. conducted iterative Internet searches to identify images and text used in identified campaigns.

To address RQ3, two coinvestigators (P. B. H. and M. Z.) searched the master list of articles included in Step 1 to identify campaign evaluations that provided outcomes data. Process evaluations were not included that reported only outputs (e.g., social media impressions generated by a campaign) but not outcomes. Evaluations for sugary beverage taxes were excluded if these did not discuss the media campaign's contribution to the tax outcome.

Figure 1 shows the conceptual model used to summarize evidence from the search for media campaign outputs and outcomes measured.^7^ P. B. H. and M. Z. constructed an evidence table that summarized the first author's last name and year published; name, location, and time frame for each campaign; target populations, communication strategies, and whether a theory or conceptual framework was used to design the campaign evaluation; and the short‐term outcomes (i.e., cognitive), midterm outcomes (i.e., individual behavior and retail), and long‐term outcomes (i.e., social norm, policy, and population health). All coinvestigators independently reviewed the evidence table for accuracy and resolved different interpretations before synthesizing in the results.

## 4 RESULTS

**FIGURE 2:**
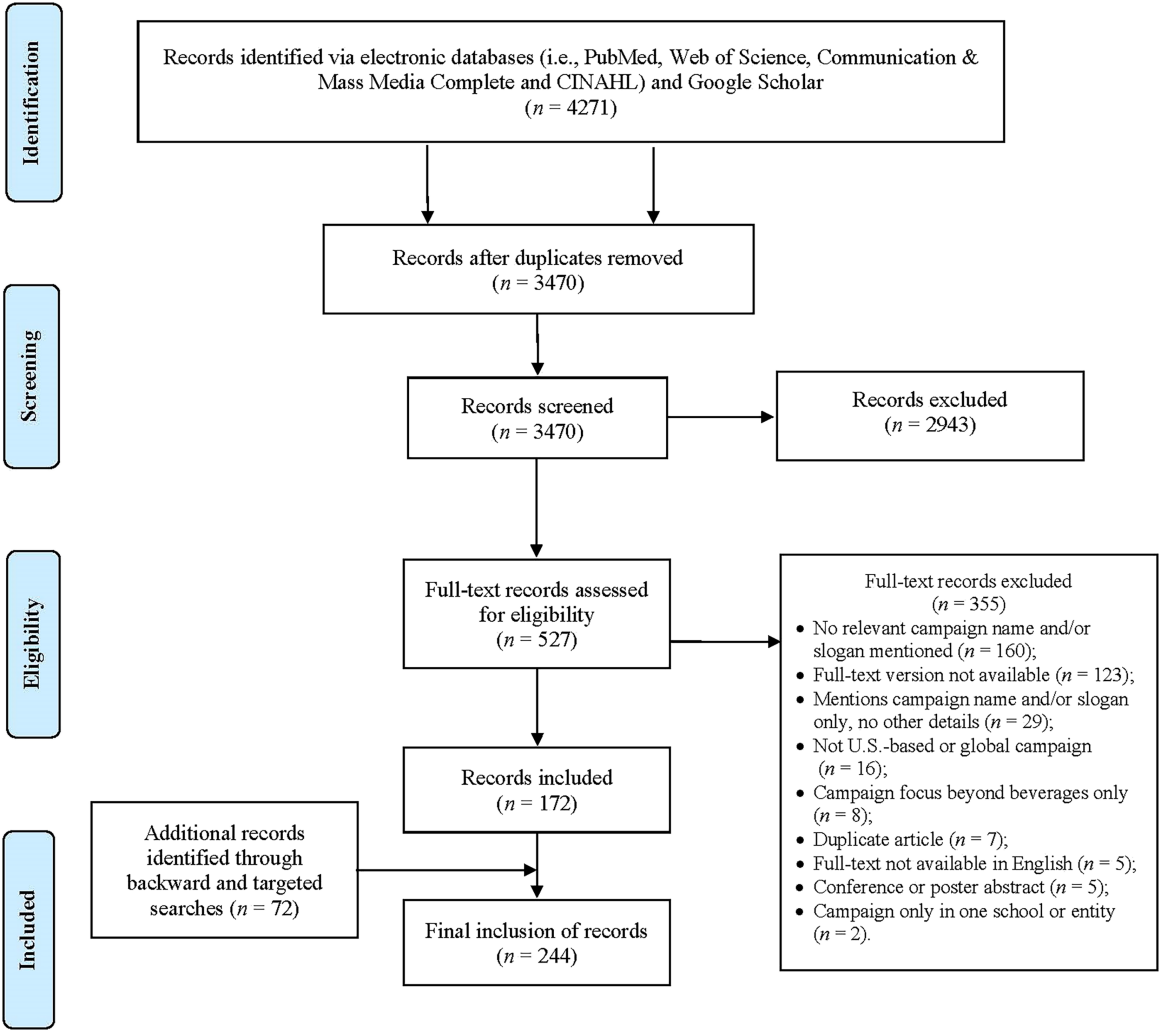
Preferred Reporting Items for Systematic Review and Meta‐Analysis flow diagram for the systematic scoping review of evidence for U.S. media campaigns used to promote or discourage nonalcoholic beverages to Americans

Figure 2 displays the PRISMA flow diagram for the systematic scoping review of evidence for U.S. media campaigns that promote or discourage beverages used to address RQ1. We identified 4271 records from the search. After removing the duplicate records, we screened the titles and abstracts for 3470 records, and we reviewed 527 full‐text records. Of the records screened, we identified 172 records that described U.S. beverage media campaigns, and we found 72 additional sources that led to 244 records for 280 unique U.S. media campaigns.

Table 2 shows 280 campaigns organized chronologically into six typology categories. Table S1 provides a comprehensive list with references for the U.S. media campaigns that either encouraged or discouraged sugary beverages or encouraged unsweetened water, milk, coffee, tea, or 100% juice, organized by the media campaign typology. More than half (66%; *n* = 184) were corporate marketing campaigns that promoted primarily branded sugary beverage products by The Coca‐Cola Company and PepsiCo.

**TABLE 2:** Evidence for 280 U.S. media campaigns organized by the typology categories and used to encourage or discourage nonalcoholic sugary beverages or encourage unsweetened water, milk, coffee, tea, or 100% juice to Americans, 1886–2021

Campaign typology category time frame (# campaigns)	Funders or supporters	Media campaign examples (campaign start date, year)
1. Corporate advertising, marketing, and entertainment campaigns (1886–2021 [*n* = 184])	Beverage firms	***Branded soda and sugary beverage campaigns*** The Coca‐Cola Company (*n* = 81): Drink Coca‐Cola and Enjoy It (1886); Delicious and Refreshing (1904); Coca‐Cola Revives and Sustains (1905); The Great National Temperance Beverage (1906); Three Million a Day (1917); Thirst Knows No Season (1922); Enjoy Thirst (1923); Refresh Yourself (1924); Six Million a Day (1925); It Had to Be Good to Get Where It Is (1926); Pure as Sunlight (1927); Around the Corner from Everywhere (1927); The Pause that Refreshes (1929); Ice Cold Sunshine (1932); The Best Friend Thirst Ever Had (1938); Thirst Asks Nothing More (1939); Whoever You Are, Whatever You Do, Wherever You May Be, When You Think Refreshment Think of Ice Cold Coca‐Cola (1939); The Only Thing Like Coca‐Cola is Coca‐Cola Itself (1942); Where There's Coke There's Hospitality (1948); Along the Highway to Anywhere (1949); What You Want is a Coke (1952); Coca‐Cola… Makes Good Things Taste Better (1956); Sign of Good Taste (1957); The Cold, Crisp Taste of Coke (1958); Be Really Refreshed (1959); Things Go Better with Coke (1963); It's the Real Thing (1969); Look Up America (1975); Coke Adds Life (1976); Have a Coke and a Smile (1976); Coke Is It! (1982); We've Got a Taste for You (1985); America's Real Choice (1985); Red, White & You (1986); Catch the Wave (1986); When Coca‐Cola is a Part of Your Life, You Can't Beat the Feeling (1987); You Can't Beat the Feeling (1988); Official Soft Drink of Summer (1989); You Can't Beat the Real Thing (1990); Always Coca‐Cola (1993); Taste It All (1993); This is Refreshment (1994); Obey Your Thirst (Sprite, 1994); The World Together, Always (1994); Just for the Taste of It (1996); Get Caught Red Handed (1997); Coca‐Cola Incredible Summer (1997); Feed the Rush (Surge, 1997); Fully Loaded Summer (Surge, 1997); You are What You Drink (1998); Surge Around the World (Surge, 1998); Coke Card (1998); No Thirst is Safe (Citra, 1998); Live Your Life (1999); Coca‐Cola. Enjoy (2000). It Could Be Your Next Coke (2000); Drink Sprite. Get Rocketcash. Buy What You Want (2000); Life Tastes Good (2001); Nature's a Mother. Drink to It (Mad River Teas, 2002); Coca‐Cola … Real (2003); Real, Make It Real (2005); The Coke Side of Life (2006); Sip Stealing. Not a Felony in All 50 States (2006); Real Coca‐Cola Taste with Zero Calories (2007); Great Taste Has Its Benefits (2007); Open Happiness (2009); Don't Settle for an Incomplete Sports Drink (Powerade, 2009); Don't Dew It (Vault, 2009); Polar Bears Catch (2012); Liquid and Linked (2012); Move to the Beat (2012); Movement is Happiness (2013); Mirage (2013); The Ahh Effect (2013); Get a Taste (2014); Share a Coke (2014); It's Beautiful (2014); Taste the Feeling (2016); #ThatsGold Rio campaign (2016); The Letter (2020); Real Magic (2021) PepsiCo, Inc. (*n* = 84): Exhilarating, Invigorating, Aids Digestion (1903); Original Pure Food Drink (1907); Delicious and Healthful (1908); Drink Pepsi‐Cola. It Will Satisfy You (1913); For All Thirsts ‐ Pepsi‐Cola (1915); Pepsi‐Cola – It Makes You Scintillate (1919); Peps You Up! (1928); Here's Health! (1929); Sparkling, Delicious (1932); It's the Best Cola Drink (1933); Double Size (1934); Refreshing and Healthful (1934); Bigger Drink, Better Taste (1934); Join the Swing to Pepsi‐Cola (1938); Twice as Much for a Nickel (1939); It's a Great American Custom (1947); Why Take Less When Pepsi's Best (1949); More Bounce to the Ounce (1950); Any Weather is Pepsi Weather (1950); The Light Refreshment (1954); Refreshing Without Filling (1955); Say Pepsi, Please (1957); Be Sociable/ The Sociables/Be Sociable, Have a Pepsi (1958); Now It's Pepsi for Those Who Think Young (1961); Come Alive! You're in the Pepsi Generation! (1963); (Taste that Beats the Others Cold) Pepsi Pours It On (1967); You've Got a Lot to Live. Pepsi's Got a Lot to Give (1969); Join the Pepsi People Feelin' Free (1973); Lipsmackin Thirst Quenchin Pepsi (1974); Have a Pepsi Day (1976); Catch the Pepsi Spirit (1979); Pepsi's Got Your Taste for Life (1981); Pepsi Now! Take the Challenge! (1983); Pepsi. The Choice of a New Generation (1984); Pepsi. A Generation Ahead (1989); Gotta Have It/Chill Out (1991); The Choice is Yours (1992); Be Young, Have Fun, Drink Pepsi (1993); Right Now (1993); Double Dutch Bus (1994); Do the Dew (1995); Nothing Else is a Pepsi (1995); Drink Pepsi. Get Stuff (1995); Change the Script (1996); Generation Next (1997); This is Diet? (1997); It's the Cola (1998); I Love My Mug (Mug, 1999); For Those Who Think Young/The Joy of Pepsi‐Cola (1999); Ask for More (1999); Too Good to Be One Calorie. But It Is. (2000); Choose Your Music (2000); Share the Joy with Music (2000); Mountain Dew Pirate Radio (2001); The Joy of Pepsi (2001); Think Young Drink Young (2002); Pepsi – It's the Cola (2003); Dare for More (2003); Catch that Pepsi Spirit (2006); Why You Doggin' Me/Taste the One That's Forever Young (2006); More Happy (2007); More Cola Taste (2007); Wake Up People! (2007); Pepsi is #1 (2008); Something for Everyone (2008); Every Sip Brings You Closer (2008); Yes You Can (2009); Zero Calories, Maximum Taste (Pepsi Max, 2009); Refresh Everything/Every Generation Refreshes the World (2009); Every Pepsi Refreshes the World (2010); Summer Time is Pepsi Time (2011); Born in the Carolinas (2011); Where There's Pepsi, There's Music (2012); Live for Now (2012); Change the Game (2012); This is How We Dew (2012); Win from Within (2012); Polar Bowl (2012); The Best Drink Created Worldwide (2012); Make Interesting Happen (2014); Out of the Blue (2015); Pepsi Generations (2018); That's What I Like (2020); Greatest of All Time (GOAT) Camp (2020) ***Branded juice campaigns (n = 10) (1994–2018)*** Memories (Welch's, 1994**);** Bite Into It (Minute Maid, Coca‐Cola, 1997); 100% Juice (Northland cranberry juice, 1997); We Only Pick the Best Fruit (Ocean Spray, 1998); Squeeze the Day (Minute Maid, Coca‐Cola, 1999); Introducing Simply Orange … 100% Unfooled Around With (Minute Maid, Coca‐Cola, 2001); Squeeze, It's a Natural (Tropicana, PepsiCo, 2009); Wake Up Your MMOJO (Minute Maid, Coca‐Cola, 2011); Tap Into Nature (Tropicana, PepsiCo, 2012); and Sip Smarter—Every Day Begins With a Sip (national, 2018) ***Branded coffee campaigns (n = 2) (1990)*** Sophisticated Taste (Taster's Choice, Nestlé, 1990); The Coffee for Intense Taste (Nescafe, Nestlé) ***Branded water campaigns (n = 5) (2000–2018)*** It's a Brett Favre Thing (Real Pure water & sports drink, 2000); L'Original (Evian, 2000–2002); We Promise Nothing (Aquafina, PepsiCo, 2002); Evian: Your Natural Source of Youth (Evian, 2004); I Wanna #Liveyoung (Danone, 2018) ***Branded nondairy, plant‐based milk campaigns (n = 3) (2000–2021)*** Get Your Soy with Silk (Danone, 2000); Silk: Milk of the Land (Danone, 2021); Wow No Cow (Oatly, 2021)
2. Corporate social repsonsibility, public relations, and cause marketing campaigns **(**2007–2021 [*n* = 16])	Industry trade associations and beverage firms	***Branded soda and sugary beverages and water campaigns*** The American Beverage Association: Balance Calories Initiative and Mixify Campaign (national, 2014) The Coca‐Cola Company: Live Positively (2010); Coca‐Cola Every Bottle has a Story (2011); The Great Meal and Together Tastes Better (2020); Together We Must (2020); Refreshing the World and Making a Difference (2021) PepsiCo. Inc.: Helping Children Get Clean Water (Ethos, 2007); Pepsi Refresh Project (2010); Pepsi We Inspire (2010); Black Lives Matter (2017); LIFEWTR, Black Art Rising (2020); Food for Good (2020); Life Unseen (2021) ***Other water campaigns*** Danone North America: Drink 1, Give 10/1 L = 10 L for Africa (Volvic water, 2008) Nestlé North America: Nestlé Waters Challenge (2019); Nestlé Pure Life (2019)
3. Social marketing campaigns **(**1970–2021 [*n* = 20])	Municipal, state, and federal government agencies in partnership with public health agencies and coalitions	***Fluid cow's milk campaigns (n = 11)*** Every Body Needs Milk (California statewide and Oregon, mid‐1970s); Milk. It Does a Body Good (national, 1980s); Lowfat Milk Campaign (New York City, NY, 1990); **1% or Less (West Virginia, California, Hawaii and Oklahoma statewide, 1995);** Got Milk? (national, 1995); Milk Mustache (national, 1997); Milk Made Better (national, 2000); White Gold (California statewide, 2008); **Choose 1% Milk (Oklahoma statewide, 2014);** Milk Life (national, 2014); You're Gonna Need Milk with That. Got Milk? (national, relaunched 2020) ***Water and juice promotion campaigns (n = 9)*** Drink Up! (national, 2013); **Live Sugarfreed (Rural regions of Kentucky, Virginia and Tennessee, 2015);** One Less Challenge (Delaware statewide, 2015); Kim and Pura (New York City, NYC, 2016); **NJ Live Sugarfreed (New Jersey statewide, 2017);** Sip Smarter—Every Day Begins With a Sip (national, 2018); Choose Water Not Sugary Drinks! (Berkeley, CA, 2020); Skip the Sugar Choose Water (Albany, CA, 2020); Be Ready. Be Hydrated. Drink Water (Seattle, WA, 2020)
4. Public information, awareness, education, and health promotion campaigns **(**1996–2020 [*n* = 27])	Municipal, state, and federal government agencies in partnership with public health agencies and coalitions	Get Coke Out of Seattle Schools (Seattle, WA, 1996); Rethink Your Drink (San Francisco, CA, 2008); Are You Pouring on the Pounds? (New York City, NY, 2009); Drinks Destroy Teeth (Indiana statewide, 2010); Rethink Your Drink (Cook County, IL, 2010); **Are You Pouring on the Pounds? (San Francisco, CA, 2010);** FatSmack (Boston, MA, 2011); Life's Sweeter with Fewer Sugary Drinks (Boston, Los Angeles, Philadelphia, San Antonio and Seattle, 2011); **Sugar Pack (Los Angeles County, CA, 2011); Sugar Bites (Contra Costa County, CA, 2011); It Starts Here (Portland & Multnomah County, OR, 2011); Get Healthy Philly (Philadelphia, PA, 2011); Howard County Unsweetened (Howard County, MD, 2012);** Rev Your Bev (Virginia statewide, 2013); Sugar Smarts/Azucar Sabia (Boston, MA, 2013); Your Kids Could Be Drinking Themselves Sick/Drink Yourself Sick (New York City, NY, 2013); **Rethink Your Drink (San Diego, CA, 2012);** Sounds Healthy (New York City, NY, 2013); Cavities Get Around (Colorado statewide, 2014); Rethink Your Drink (cities nationwide 2015); **Choose Water (Los Angeles, CA, 2015);** Drink NYC Tap Water (New York City, NY, 2016); Sour Side of Sweet (New York City, NY, 2017); Hidden Sugar (Denver, CO, 2017); Rethink Your Drink (Arkansas statewide, 2017); Healthy for Good Sip Smarter (national, 2018); Healthy Drinks Healthy Kids (national, 2020)
5. Media advocacy and countermarketing campaigns **(**2007–2015 [*n* = 10])	Municipal, state, and national government agencies; public health advocacy organizations and coalitions	Global Dump Soft Drinks (national and international, 2007); Dunk the Junk (San Francisco, CA, 2011); The Real Bears (national, 2012); Kick the Can (California statewide and national, 2012); Soda Sucks (California statewide, 2012); **The Bigger Picture (San Francisco, CA, 2013)**; Coming Together: Translated (national, 2013); Open Truth Now (San Francisco, CA, 2015); ‘Share a Coke’ with Obesity (national, 2015); Change the Tune (national, 2015)
6. Political and public policy campaigns **(**2012–2018 [*n* = 23])	Municipal and state government agencies; public health advocacy organizations and coalitions; and industry trade organizations	***Pro‐sugary beverage tax campaigns (n = 11)*** Richmond Fit For Life (Richmond, CA, 2012); Choose Health SF (San Francisco, CA, 2014); Healthy Diné Nation Act (Navajo Nation, 2014); Vote Yes on Measure D/Berkeley vs. Big Soda (Berkeley, CA, 2014); Vote Yes on Proposition V (San Francisco, CA, 2016); Vote Yes on Measure HH to Protect our Children's Health/Oakland vs. Big Soda (Oakland, CA, 2016); Yes on O1 (Albany, CA, 2016); Vote Yes on Soda Tax Because Our Kids Are Worth It! (Philadelphia, PA, 2016); Healthy Boulder Kids Campaign (Boulder, CO, 2016); Pre‐K for Santa Fe (Santa Fe, NM, 2017); Seattle Healthy Kids Coalition (Seattle, WA, 2017) ***Anti‐sugary beverage tax and preemption campaigns (n = 12)*** No on N campaign (Richmond, CA, 2012); No SF Beverage Tax/Vote No on E (San Francisco, CA 2014); No Berkeley Beverage Tax (Berkeley, CA, 2014); No on V/Enough is Enough: Do not Tax Our Groceries (San Francisco, CA, 2016); No Oakland Grocery Tax/No on HH (Oakland, CA, 2016); No on O1 (Albany, CA, 2016); No Philly Grocery Tax (Philadelphia, PA, 2016); Better Way for Santa Fe & Pre‐K (Santa Fe, NM, 2017); Yes! To Affordable Groceries (Washington statewide, 2017); Vote Yes on Measure 103 to Keep Our Groceries Tax Free (Oregon, 2017); Keep Seattle Liveable for All Coalition (Seattle, WA, 2017); Keep Groceries Affordable Act (California, 2018)

*Note*: The 24 bolded campaigns in Table 2 were evaluated and the outcomes are described in Table 3.

The RQ1 and RQ2 evidence for selected campaigns is synthesized below for each media campaign typology category including corporate advertising, marketing, and entertainment campaigns (Section 4.1); corporate social responsibility, public relations, and cause marketing campaigns (Section 4.2); social marketing campaigns (Section 4.3); public information, awareness, education, and health promotion campaigns (Section 4.4); media advocacy and countermarketing campaigns (Section 4.5); and public policy and political campaigns (Section 4.6). Section 4.7 describes RQ3 evidence from 24 evaluations for 20 unique campaigns across the typology.

The RQ1 results showed a U.S. media landscape dominated by corporate marketing campaigns for branded sugary beverages (65.8%; *n* = 184) followed by public awareness (9.6%; *n* = 27), public policy (8.2%; *n* = 23), social marketing (7.1%; *n* = 20), corporate social responsibility (5.7%; *n* = 16), and countermarketing (3.6%; *n* = 10) campaigns.

### 4.1 Corporate advertising, marketing, and entertainment campaigns

Corporate advertising, marketing, and entertainment campaigns are used to promote a specific brand or branded product to increase sales, purchase, and consumption.^7^ The scoping review identified 184 corporate advertising, marketing, and entertainment campaigns. The majority of these campaigns were used to promote sugary beverage brands by The Coca‐Cola Company (*n* = 81) that ranged from Drink Coca‐Cola and Enjoy It (1886) to Real Magic (2021)^33^ and PepsiCo (*n* = 84) that spanned from Exhilarating, Invigorating, Aids Digestion (1903)^34^ to Greatest of All Time Camp (2020).^35^ We did not include Pepsi or Coke campaigns relaunched with the same campaign slogan at a later time period. No Nestlé campaigns were identified that promoted sugary beverage products in the U.S. context.

We identified branded juice campaigns (*n* = 9) including six for The Coca‐Cola Company's Minute Maid and PepsiCo's Tropicana brands. The earliest juice campaign was Welch's Memories (1994),^36^ and the most recent was PepsiCo's Tropicana brand's Tap into Nature (2012).^37^ Five branded water campaigns (2000–2018) included PepsiCo's Aquafina (2002) and several Danone campaigns (Table 2).^38^ We found no corporate campaigns that promoted The Coca‐Cola Company's Dasani brand or Nestlé's Pure Life water brand. We identified two coffee campaigns for Nestlé's brand Nescafé. Three branded nondairy, plant‐based milk campaigns were identified including Danone's Silk brand Get Your Soy with Silk (2000)^39^ for soy milk and Silk: Milk of the Land for almond milk (2021)^40^ and Oatley's Wow No Cow oat milk campaign (2021).^41^ No branded cow's milk or tea campaigns were identified. We found no evaluations that reported corporate marketing campaign outcomes.

### 4.2 Corporate social responsibility, public relations, and cause marketing campaigns

Corporate social responsibility, public relations, and cause marketing campaigns are used by companies to promote their brand or products with a social or environmental cause, present the firm in a positive way, or defend the company's reputation.^7^, ^42^ We identified 16 campaigns for this category implemented by the American Beverage Association (ABA) (*n* = 1), The Coca‐Cola Company (*n* = 5), PepsiCo Inc. (*n* = 7), Nestlé (*n* = 2), and Danone (*n* = 1). Five of 16 campaigns promoted branded water products, which differed from the corporate advertising campaigns because the sale of the water was tied to a social or environmental cause, such as providing clean drinking water for people in Africa.

These campaigns have promoted healthy beverage behaviors; supported social, racial, and ethnic justice issues; and promoted environmental sustainability.^43^, ^44^, ^45^, ^46^, ^47^ The earliest campaign identified was Danone's Drink 1, Give 10 or 1 L = 10 L for Africa campaign (2008) for Volvic branded water.^48^ Recent campaigns were The Coca‐Cola Company's Refreshing the World and Making a Difference (2021)^49^ and PepsiCo's Life Unseen for LIFEWTR (2021).^50^ Section 4.7 describes two evaluations for the ABA's Balance Calories Initiative (BCI)^51^, ^52^ that was launched in 2014 with the Alliance for a Healthier Generation and The Coca‐Cola Company, Dr. Pepper Snapple Group (now Keurig Dr. Pepper), and PepsiCo Inc. The BCI evolved out of the Mixify campaign (2014) and aimed to reduce the beverage calories consumed by Americans by 20% by 2025.^53^

### 4.3 Social marketing campaigns

Social marketing campaigns apply commercial marketing principles and techniques to plan, implement, and evaluate media campaigns to influence the voluntary behaviors of target audiences or populations that achieve health or social goals.^7^ Several social marketing campaigns were described in the literature. For this study, we categorized campaigns as social marketing if they used marketing principles to promote positive behavior change to increase consumption of unsweetened fluid milk, coffee, tea, or 100% unsweetened juice recommended by the DGA as healthy beverages, rather than to encourage Americans to reduce or stop sugary beverage consumption. We separated the corporate branded water and juice campaigns from social marketing campaigns because the latter promote unbranded water, juice, or milk, and many of the campaigns spanned years or even decades. These campaigns were also supported by or implemented in partnership with government, civil society organizations, or industry trade organizations, which we found to be unique from traditional corporate marketing campaigns that were solely industry funded.

Table 2 presents the results for 20 social marketing campaigns, of which 11 were for low‐fat or fat‐free fluid cow's milk^54^, ^55^, ^56^, ^57^, ^58^, ^59^, ^60^, ^61^, ^62^; nine that promoted water (two that were evaluated)^63^, ^64^; and the Juice Products Association's Sip Smarter campaign (2018) that promoted 100% juice.^65^ Eight were national campaigns, and 12 were implemented in a city, county, or state.

The earliest fluid cow's milk social marketing campaign was the Every Body Needs Milk (1970s)^54^ that promoted milk in California (CA). The most recent fluid cow's milk campaign was the relaunch of Got Milk? (2020), which was implemented nationwide from 1995 to 2014.^66^ Drink Up! (2013–2016)^67^ was a water‐promotion campaign launched by the Partnership for a Healthier America. In 2020, several water‐promotion campaigns were launched including Choose Water Not Sugary Drinks! in Berkeley, CA,^68^ Skip the Sugar, Choose Water in Albany, CA,^69^ and Be Ready. Be Hydrated. Drink Water in Seattle, Washington (WA).^70^ Section 4.7 describes 10 evaluations for seven social marketing campaigns.

### 4.4 Public information, awareness, education, or health promotion campaigns

Public information, awareness, education, or health promotion campaigns educate individuals or populations about the harms or benefits of a certain behavior, product, or health‐related issue.^7^ The search produced 27 campaigns: two national and 25 city or county campaigns implemented in 13 states. The earliest campaign was Get Coke Out of Seattle Schools (1996) implemented in Seattle, WA, that encouraged parents and school boards to revoke The Coca‐Cola Company's pouring rights in the school district.^30^ The Healthy Drinks Healthy Kids campaign (2020),^71^ led by the U.S. Healthy Eating Research National Program and four organizations, was based on the 2019 healthy beverage consensus guidelines for children birth to age 5 years.^12^ Section 4.7 describes 12 evaluations for 11 campaigns.^72^, ^73^, ^74^, ^75^, ^76^, ^77^, ^78^, ^79^, ^80^, ^81^, ^82^, ^83^

### 4.5 Media advocacy and countermarketing campaigns

Media advocacy or countermarketing campaigns catalyze community support to change corporate marketing practices and are often led by advocacy or social justice organizations to address a public health or policy issue.^7^ This scoping review identified 10 campaigns, of which six were implemented nationally. The Global Dump Soft Drinks (2007) was implemented in the United States and internationally and spearheaded by the CSPI in partnership with other international organizations.^84^ Five countermarketing campaigns were implemented in California including Dunk the Junk (2011), Soda Sucks (2012), Kick the Can (2012), The Bigger Picture (2013), and Open Truth Now (2015) (Table S2). The CSPI's Change the Tune (2015) national campaign aimed to shift the pro‐sugary beverage narrative.^85^ Section 4.7 and Table 3 summarize the evaluation results for The Bigger Picture.^86^

**TABLE 3:** Concise evidence summary of 24 evaluations for 20 unique U.S. beverage media campaigns organized by author, year, campaign name, typology category, and outcomes, 1992–2021

First author's last name, year published, and campaign name	Typology	Short‐term outcomes and cognitive outcomes			Midterm outcomes, behavioral outcomes, and retail outcomes			Long‐term outcomes social norm, policy, and population health outcomes		
		Preferences, beliefs, attitudes, awareness, and support of HB policy	HB knowledge	HB intention to consume	HB intake	HB retail sales	Communicated campaign message to another	Social norm	Institutional policy	Population health
Bogart et al. (2019)^51^; *Balance Calories Initiative*	2	+	−				+			
Cohen et al. (2018)^52^; *Balance Calories Initiative*	2					No change				
Bonnevie et al. (2020)^63^; *NJ Live Sugarfreed*	3	+	+			+				
Farley et al. (2017)^64^; *Live Sugarfreed*	3	+	+			+				
Hinckle et al. (2008)^55^; *Adelante Con Leche Semi‐descremada 1%*	3					+				
John et al. (2019)^56^; *1% Low‐Fat Milk has Perks! Choose 1%* *Milk: A Health Family Choice*	3				+	+				
Maddock et al. (2007)^57^; *1% or Less*	3	+	+	+	+	+		No change	+	
Reger et al. (1998)^58^; *1% or Less*	3	+			+	+				
Reger et al. (1999)^59^; *1% or Less*	3				+	+				
Reger et al. (2000)^60^; *1% or Less*	3				+	+				
Wechsler and Wernick (1992)^61^; *Low‐fat Milk Campaign*	3					+			+	
Wootan et al. (2005)^62^; *1% or Less* ^a^	3				+	+ and no change				
Barragan et al. (2014)^72^; *Choose Health LA Sugar Pack*	4		+	+						
Bleakley et al. (2018)^73^; *Get Healthy Philly*	4	+	+	+						
Boehm et al. (2021)^74^; *Howard County Unsweetened*	4				+					
Boles et al. (2014)^75^; *It Starts Here*	4	+	+	+	No change					
Caldwell et al. (2020)^76^; *Choose Water*	4	+		+			+			
Hartigan et al. (2017)^77^; *Rethink Your Drink*	4					+				
Hornsby et al. (2017)^78^; *Cavities Get Around*	4		+		+				+	
James et al. (2020)^79^; *Shape Your Future –Rethink Your Drink*	4		+		+					
Maghrabi et al. (2021)^80^; *Rethink Your Drink*	4				+					
Robles et al. (2015)^81^; *Choose Health LA Sugar Pack*	4	+		+						
Samuels et al. (2010)^82^; *Are You Pouring on the Pounds?* ^b^	4	+/−	+	+/−	No change					
Schwartz et al. (2017)^83^; *Howard County Unsweetened*	4				+					
Schillinger et al. (2018)^86^; *The Bigger Picture*	5	+	+							
Total studies that measured outcomes		14			21			3		

*Note*: The evaluations for 20 unique campaigns were implemented over 30 years (1992–2021) across 14 states including Alabama, California, Colorado, Hawaii, Kentucky, New Jersey, New York, Maryland, Mississippi, Oklahoma, Oregon, Pennsylvania, Virginia, and West Virginia. See Table S2 for details for each campaign. The most frequent response categories for short‐, middle‐, and long‐term outcomes are reflected by a slightly darker color (i.e., HB knowledge, HB intake, and institutional policy). Healthy beverage (HB); (+) = positive healthy beverage change (e.g., increased water intake and/or reduced sugary beverage or juice intake) and (−) = negative healthy beverage change.

^a^ Wootan et al.^62^evaluated milk sales in four different communities. Two of the four communities found a positive change in the sale healthier milks promoted, and there was no significant change found in the remaining two communities.

^b^ Samuels et al.^82^ did not conduct a baseline assessment, so it is not possible to report changes in the postcampaign evaluation. Some findings were mixed, for example, respondents were in favor of taxation of sugary beverages (e.g., for generating funds) but also reported that taxation may have limited effectiveness to reduce sugary beverage consumption.

### 4.6 Public policy and political campaigns

Public policy or political campaigns catalyze public support or opposition for legislation and laws to restrict sales or access to products or discourage behaviors that harm human health or the environment.^7^ We identified 23 relevant campaigns, including pro‐sugary beverage tax campaigns (*n* = 11) and anti‐sugary beverage tax and state preemption campaigns (*n* = 12). Twenty campaigns used print, broadcast, and/or social media to support or oppose a proposed sugary beverage tax in nine cities and the Navajo Nation.^87^ The earliest pro‐ and anti‐tax campaigns were in Richmond, CA, called Richmond Fit for Life (pro‐tax) and No On N (anti‐tax) (2012)^88^ where a sugary beverage tax was not enacted.

The Navajo Nation, which covers northeastern Arizona, southeastern Utah, and northwestern New Mexico, successfully enacted the Healthy Diné Nation Act (2014)^89^ that was renewed and reauthorized in 2020.^90^ Seven cities successfully implemented a sugary beverage tax including Berkeley, CA (2014)^91^; Albany, CA (2016)^92^; Oakland, CA (2016)^93^; San Francisco, CA (2016)^94^; Boulder, CO (2016)^95^; Philadelphia, PA (2016)^96^; and Seattle, WA (2018)^97^ (Table 2). The most recent pro‐ and anti‐tax campaigns were in Seattle, WA (2018). The ABA‐funded state preemption campaigns in Washington (2017), Oregon (2017), and California (2018) used anti‐tax media messages such as “keep groceries tax free and affordable” to gain public support to prevent future sugary beverage tax laws.^24^ We found no published evaluations of outcomes for these public policy campaigns.

### 4.7 U.S. beverage media campaign evaluations

Table 3 provides a concise summary of 24 evaluations for 20 unique U.S. beverage media campaigns. The evidence is organized by author, year, campaign name, typology category, and reported outcomes (1992–2021). Table S2 provides a detailed summary of each campaign's goal, time frame, target audiences, and media strategies; the theory, model, or conceptual framework reported; and short‐term cognitive outcomes (i.e., awareness, knowledge, attitudes, beliefs, and intentions), midterm outcomes (i.e., retail and behaviors), and long‐term outcomes (i.e., social norms, policies, and population health).

**FIGURE 3:**
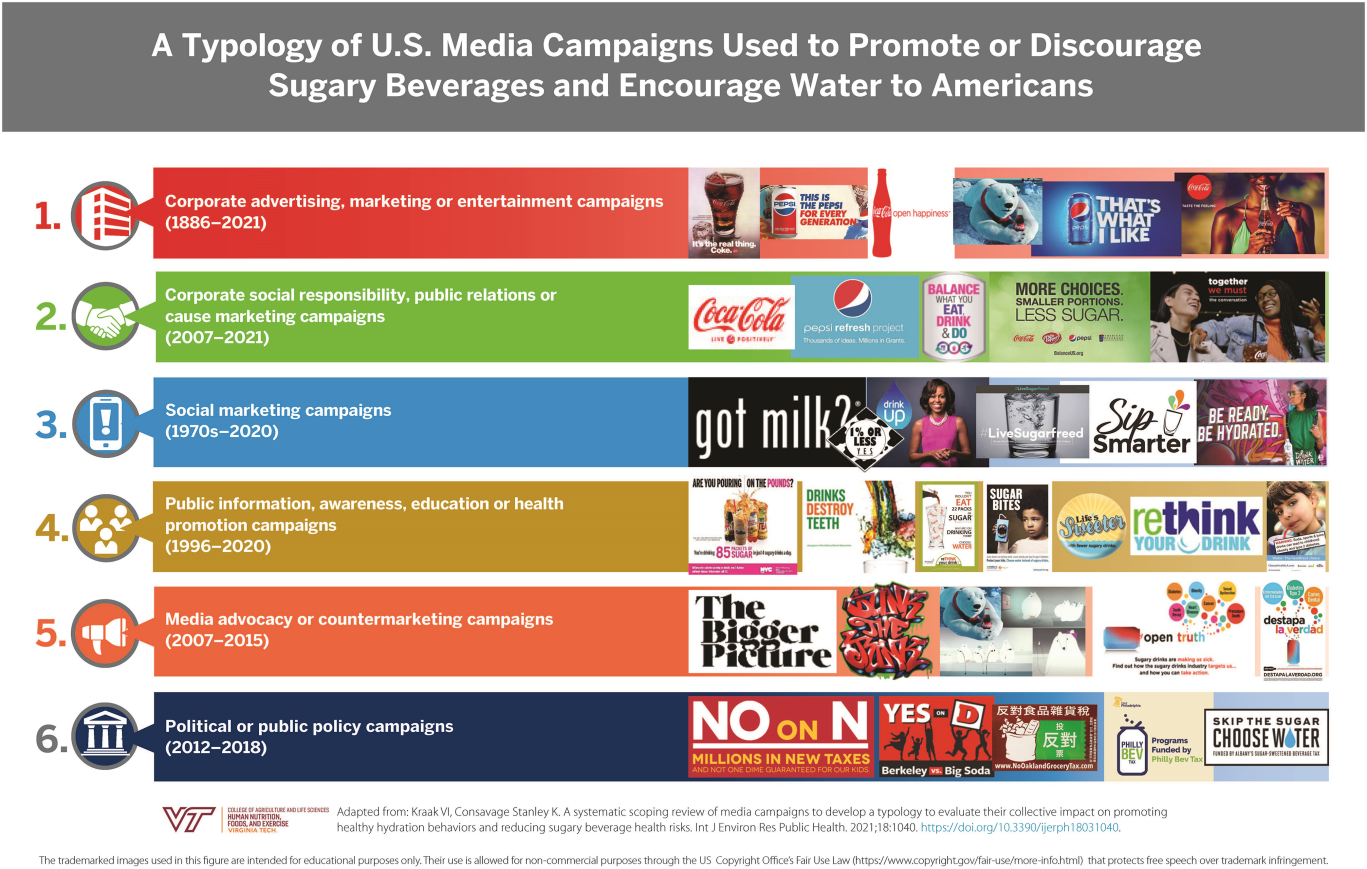
Typology of U.S. media campaigns used to promote or discourage sugary beverages and encourage water, milk, or 100% juice to Americans

**FIGURE 4:**
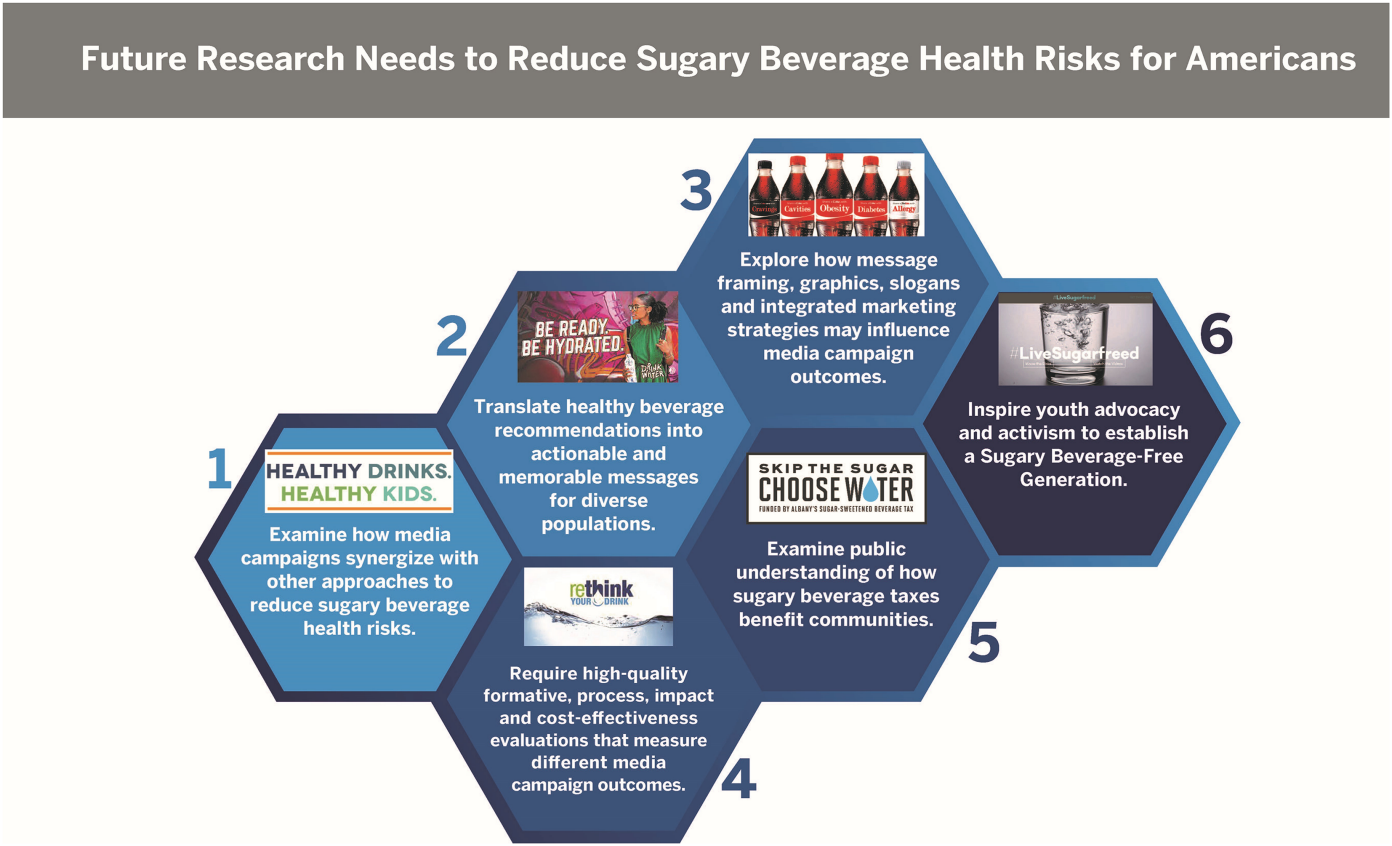
Future research needs to reduce sugary beverage health risks for Americans

Figure 3 shows images of selected examples across the six typology categories. Table S3 provides the fair use evaluation documentation for the media campaign images used in Table S2 and Figures 3 and 4. The U.S. “nominative fair use” doctrine protects free speech over trademark infringement when images are used for noncommercial educational purposes.^98^

Of the 20 unique campaigns evaluated across 14 states, a theory, model, or conceptual framework was reported for nine campaigns, including 1% Low‐Fat Milk has Perks! and Choose 1% Milk: A Health Family Choice,^56^ 1% or Less,^57^ NJ Live Sugarfreed,^63^ Get Healthy Philly,^73^ Howard County Unsweetened,^74^, ^83^ It Starts Here,^75^ Cavities Get Around,^78^ and Choose Health LA Sugar Pack^81^ (Table S2). Eleven of the 20 campaigns were evaluated over 4 to 15 weeks, and two campaigns were evaluated over 2 to 4 years.

Six evaluations reported racial or ethnic minorities as the target populations^55^, ^57^, ^61^, ^63^, ^74^, ^86^; and five evaluations reported on low‐income populations.^51^, ^52^, ^55^, ^56^, ^63^ Only two evaluations^74^, ^86^ had involved ethnically diverse youth who are disproportionately impacted by sugary beverage marketing.^17^

The evaluations for 14 studies^51^, ^52^, ^57^, ^58^, ^63^, ^64^, ^72^, ^73^, ^75^, ^76^, ^78^, ^79^, ^81^, ^82^, ^86^ reported positive changes to short‐term cognitive outcomes, especially knowledge about the benefits of water or milk and the consequences of drinking sugary beverages. Two independent evaluations^51^, ^52^ were identified for the ABA‐funded BCI campaign that aimed to decrease per capita intake of energy from beverages by 20% by 2025. Bogart et al.^51^ found a decrease in healthy beverage knowledge among 8–10 low‐income intervention communities in the Mississippi (MS) Delta region that includes Alabama and MS and CA. This study also documented an increase in parents and youth who reported that drinking more sugary drinks was acceptable or that they needed to balance healthy and unhealthy beverage intake.^51^ Cohen et al.^52^ found no change in sugary beverage sales in the intervention communities after two years. The two BCI evaluations^51^, ^52^ reported a decrease in healthy beverage knowledge of targeted populations.^51^ In contrast, evaluations for 10 unique campaigns found a positive knowledge change attributed to the media campaign.^57^, ^63^, ^64^, ^72^, ^73^, ^75^, ^78^, ^79^, ^82^, ^86^

Twenty‐one studies measured midterm behavioral outcomes including healthy beverage intake, change in retail sales of healthy beverage, or whether a respondent communicated with another person(s) about the campaign or messages. Eleven of 13 studies that reported on sugary beverage consumption found decreased intake^56^, ^57^, ^58^, ^59^, ^60^, ^62^, ^74^, ^78^, ^79^, ^80^, ^83^ for six unique campaigns. The Cavities Get Around campaign evaluation was the only one that reported changes in healthy beverage intake among young children.^73^ No changes were reported for San Francisco's Pouring on the Pounds (2010) and Oregon's It Starts Here (2011) campaigns^75^, ^82^ (Table 3). Eleven of 12 studies that assessed retail outcomes reported an increase in healthy beverage sales.^55^, ^56^, ^57^, ^58^, ^59^, ^60^, ^61^, ^62^, ^63^, ^64^, ^77^

Three studies^57^, ^61^, ^78^ reported having measured long‐term outcomes for institutional policy changes including public schools that provided low‐fat milk in a statewide 1% or Less Campaign in Hawaii,^57^ preschool and child‐care settings that established beverage policies for the Low‐Fat Milk Campaign in New York City (NYC),^61^ and a statewide policy change attributed to the Cavities Get Around campaign that prohibited child‐care centers in Colorado from serving sugary beverages and that capped 100% juice to two servings weekly.^78^ Maddock et al.^57^ found no change in social norms at the end of Hawaii's 6‐week statewide 1% or Less campaign. No study reported individual or population health outcomes for the 20 campaigns reviewed (Table S2).

## 5 DISCUSSION

This is the first systematic scoping review of U.S. media campaigns to use a comprehensive media campaign typology to categorize various campaigns implemented over decades to promote or discourage nonalcoholic beverages to Americans. The scoping review found that the corporate advertising, marketing, and entertainment campaigns funded and implemented by The Coca‐Cola Company and PepsiCo dominated the beverage media campaign landscape for more than 130 years (1895 to 2021) to promote branded sugary beverage products. More recently, advertising campaigns for branded juice, water, coffee, and plant‐based milk campaigns used IMC strategies to appeal to customers but were less prevalent.

Only 20 of the 280 campaigns (1992–2021) were evaluated across four typology categories: corporate social responsibility (*n* = 1), social marketing (*n* = 7), public information (*n* = 11), and media advocacy (*n* = 1). These campaigns were used to reduce sugary beverage sales and intake or to increase unsweetened water, 100% juice, or low‐fat milk sales and intake. Despite extensive descriptive evidence, we found no published evaluations for the corporate advertising and marketing or public policy campaigns.

The short‐term outcomes reported for the 20 campaigns indicated that public health campaigns positively affected short‐term healthy beverage behaviors and sales. Only three campaigns^57^, ^61^, ^79^ reported institutional policy changes to promote low‐fat milk or water, and Maddock et al.^57^ found no change in social norms for the *1% or Less* campaign. No campaign reported population health outcomes.

Social change is a process that occurs over time to influence social norms, attitudes, and behaviors of populations.^7^, ^99^ Media campaigns may contribute to social change movements, and their influence may be enhanced by PSE strategies to achieve desirable outcomes.^7^ Corporate advertising and marketing campaigns (typology category 1) were used to promote sugary beverages, and the corporate social responsibility and public relations campaigns (typology category 2) were generously funded to build trust and brand loyalty among customers. These campaigns undermine a social change movement to support a Sugary Beverage‐Free Generation.^7^ In contrast, the social marketing, public health, and countermarketing campaigns (typology categories 3–5) that could support a sugary beverage social change movement had modest investments. Evaluations showed an increase in awareness and knowledge about healthy and unhealthy beverages and influence individual behaviors; few campaigns influenced policies; and none influenced long‐term population behaviors.

Although certain campaigns could have been classified in different typology categories, we used the campaign typology goal^7^ to guide our decision to place several campaigns into the social marketing category 3. These include the Got Milk?, Milk Mustache, and Milk Life advertising campaigns (1995–2021); the Juice Products Association's Sip Smarter—Every Day Begins With a Sip campaign (2018); and Seattle's Be Ready. Be Hydrated. Drink Water (2020) public awareness and countermarketing campaign. The campaign messages aligned with the U.S. expert recommendations for Americans to drink milk, 100% juice, and water. Therefore, all of the milk‐promotion campaigns were placed together in the social marketing category rather than to divide the milk industry‐funded campaigns from the community‐based *1% or Less* milk campaigns.

An extensive published literature has reported the impact of sugary beverage taxes on purchasing behaviors for healthy beverages but was beyond the scope of this study. The sugary beverage tax revenue generated for seven U.S. jurisdictions (2018–2021) approximated U.S. $134 million dollars and was allocated for health, social, and community capital investments, infrastructure improvements, and workforce development.^100^ The Skip the Sugar, Choose Water campaign explicitly stated that it was funded by the sugary beverage tax in Albany, CA^69^ (Figure 3). However, we found no evaluation for a media campaign that had communicated the benefits of sugary beverage taxes for communities. This is a major knowledge gap to understand how to influence Americans' beverage perceptions and behaviors. Therefore, media campaign planners must use insights from previous campaigns to inform future campaigns.

### 5.1 Strengths and limitations

A strength of this study was the use of a media campaign typology that had a defined goal for each of the six distinct campaign categories, which enabled us to understand the complex U.S. beverage campaign landscape using a systematic evidence review process. This study had several limitations. The study purpose was to apply the typology to examine U.S. examples for the six typology categories and was not intended to be an exhaustive search. Another limitation was that we searched the English language literature through May 2021 and did not capture evidence published after that date or in other languages. The 20 evaluations did not provide sufficient details to understand how the campaigns were designed based on factors such as race, ethnicity, culture, age, or literacy capacity.

### 5.2 Future research

This section builds on the study findings and combines these findings with additional literature to suggest future research needs for media campaigns to reduce sugary beverage health risks for Americans and to support a social change movement to establish a Sugary Beverage‐Free Generation (Figure 4).

First, future research should examine how media campaigns may synergize with other PSE approaches to influence diverse populations, age groups, and settings.^101^, ^102^, ^103^, ^105^, ^106^, ^107^, ^108^ Second, research is needed to translate healthy beverage recommendations^9^, ^10^, ^11^, ^71^, ^109^ into actionable and memorable messages for diverse populations.

Third, future research could explore how message framing, graphics, slogans, and IMC strategies may influence media campaign outcomes using the typology. Fourth, funders should provide multiyear grants that will enable campaign planners to adequately fund high‐quality evaluations and use conceptual frameworks or theories to plan and evaluate campaigns. Many costs are associated with the packaged strategies for media campaigns that vary depending on context; therefore, cost‐effectiveness evaluations are also needed to inform future efforts.

Fifth, future research should examine the public's understanding of how sugary beverage taxes benefit communities, including Black and Latinx youth and parents who may support pro‐tax policies when the benefits are communicated effectively.^110^, ^111^ A study published after we completed our review reported that culturally tailored countermarketing messages delivered to Latinx parents for 6 weeks in an online simulated store on Facebook, either alone or combined with water promotion messages, had influenced parents to select fewer sweetened fruit drinks and increase water for their children.^112^ Whether these behaviors are sustained over time requires further investigation given the challenge for public health messages to compete with highly funded corporate marketing campaigns for sugary beverages.^113^ Sixth, future research should apply tobacco control campaign insights^114^ to engage and inspire youth activism^115^, ^116^ to support a social change movement for a Sugary Beverage‐Free Generation.

## 6 CONCLUSION

Public health media campaigns may influence the awareness, attitudes, preferences, behaviors, and health outcomes of individuals and populations. However, these campaigns must be examined within the broader environment. This systematic scoping review of U.S. beverage media campaigns categorized evidence into a proof‐of‐concept typology. As the corporate marketing of sugary beverage products targeted to Americans increases across digital platforms, media campaigns that influence awareness and attitudes about the harms of sugary beverages but do not address equity, health disparities, and the commercial determinants of health are unlikely to produce sustainable changes. Six future research needs are described to understand how media campaigns may support a social change movement to promote healthy hydration behaviors and reduce sugary beverage health risks for populations.

The copyright and trademarked images used in this paper are intended for noncommercial educational purposes only and allowed by the U.S. “nominative fair use” doctrine. A fair use evaluation was completed for using these images as illustrative examples.^97^

## INSTITUTIONAL REVIEW BOARD STATEMENT

Not applicable.

## CONFLICT OF INTEREST

The authors declare no conflicts of interest related to this paper.

## AUTHOR CONTRIBUTIONS

V. I. K. and K. C. S. conceptualized the research questions and study design; K. C. S. designed the search strategy in consultation with V. I. K. and conducted the systematic scoping review with P. B. H.; V. I. K. wrote the first draft manuscript and led the submission process. K. C. S., P. B. H., and M. Z. compiled the evidence tables and provided input into subsequent manuscript drafts. All authors read and approved the final submission.

## DISCLOSURES

V. I. K., K. C. S., P. B. H., and M. Z. did not receive any funding from the commercial or private‐sector entities for research or consulting and have no conflicts of interest related to the content of this manuscript. This research did not involve human subjects; therefore, it was exempt from institutional review board requirements.

## Supporting information


**Table S1:** Comprehensive list of U.S. media campaigns (n = 280) used to encourage or discourage non‐alcoholic sugary beverages or encourage unsweetened water, milk, coffee, tea or 100% juice, organized chronologically by the media campaign typology.

**Table S2:** Comprehensive evidence summary of 24 evaluations for 20 unique U.S. beverage media campaigns organized by the typology category, goal, target population and outcomes, 1992–2021.

**Table S3:** Fair use evaluation documentation for the images used in Figures 3 and 4 and Supplemental Table 2 to illustrate beverage campaigns used to promote or discourage sugary beverages and encourage water, milk or 100% juice to Americans, 1886–2021.

## Footnotes

*
**Integrated marketing communications** (IMC) strategies include advertising, personal selling, public relations, and sales promotion combined with marketing techniques (i.e., branding, celebrity endorsement, premiums, product placement, point of sale, and sponsorship) delivered across media platforms and diverse settings to influence the diet‐related cognitive, behavioral, and health outcomes of individuals and populations.
