# Supplementary material for: How have media campaigns been used to promote and discourage healthy and unhealthy beverages in the United States? A systematic scoping review to inform future research to reduce sugary beverage health risks
Source: Obes Rev. 2022 Feb 9;23(5):e13425. doi: 10.1111/obr.13425 (PMC9286342; doi:10.1111/obr.13425)
Supplement: Supplementary file 1 — Table S1: Comprehensive list of U.S. media campaigns (n = 280) used to encourage or discourage non‐alcoholic sugary beverages or encourage unsweetened water, milk, coffee, tea or 100% juice, organized chronologically by the media campaign typology. [file OBR-23-0-s002.pdf]

How Have Media Campaigns Been Used to Promote and Discourage Healthy and Unhealthy Beverages in the United States? A Systematic Scoping Review to Inform Future Research to Reduce Sugary Beverage Health Risks

Vivica I. Kraak[1,*], Katherine Consavage Stanley,[2] Paige B. Harrigan[3] and Mi Zhou[4]

Author contact information and affiliations
[§] *Corresponding author*
[1] Associate Professor of Food and Nutrition Policy, Department of Human Nutrition, Foods, and Exercise, Virginia Tech, 257 Wallace Hall, 295 West Campus Drive, Blacksburg, Virginia 24061 USA; phone: (440) 985-8048; email: vivica51@vt.edu; ORCID: https://orcid.org/0000-0002-9303-5530
[2] Graduate Teaching Assistant and PhD Student, Department of Human Nutrition, Foods, and Exercise, Virginia Tech, Wallace Hall, 295 West Campus Drive, Blacksburg, VA 24061 USA; email: kconsavage@vt.edu; ORCID: https://orcid.org/0000-0001-6022-8697
[3] Graduate Research Assistant and PhD Student, Department of Human Nutrition, Foods, and Exercise, Virginia Tech, Wallace Hall, 295 West Campus Drive, Blacksburg, VA 24061 USA; email: paigeharrigan@vt.edu; ORCID: https://orcid.org/0000-0001-6647-9593
[4] Post-doctoral Research Fellow, Department of Public Health, University of California Merced 95344, USA; email: mzhou30@ucmerced.edu; ORCID: https://orcid.org/0000-0001-8822-4188

Supplemental Table 1: Comprehensive list of U.S. media campaigns (n=280) used to encourage or discourage non-alcoholic sugary beverages or encourage unsweetened water, milk, coffee, tea or 100 percent juice, organized chronologically by the media campaign typology.

Supplemental Table 2: Comprehensive evidence summary of 24 evaluations for 20 unique U.S. beverage media campaigns organized by the typology category, goal, target population and outcomes, 1992-2021.

Supplemental Table 3: Fair use evaluation documentation for the images used in Figures 3 and 4 and Supplemental Table 2 to illustrate beverage campaigns used to promote or discourage sugary beverages and encourage water, milk or 100 percent juice to Americans, 1886-2021.

Supplemental Table 1

Comprehensive list of U.S. media campaigns (n=280) used to encourage or discourage non-alcoholic sugary beverages or encourage unsweetened water, milk, coffee, tea or 100 percent juice, organized chronologically by the media campaign typology.

**Campaign Typology Category**<br>Campaign Name (national, city, state)	**Date Launched**	**References**
**1. Corporate advertising, marketing or entertainment campaigns** (*n* = 184)		
The Coca-Cola Company (national) *(n = 81)*		
Drink Coca-Cola and Enjoy It	1886	The Coca-Cola Company 2020[1]
Delicious and Refreshing	1904	Zmuda 2009[2]; The Coca-Cola Company 2020[1]
Coca-Cola Revives and Sustains	1905	The Coca-Cola Company 2020[1]
The Great National Temperance Beverage	1906	The Coca-Cola Company 2020[1]
Three Million a Day	1917	The Coca-Cola Company 2020[1]
Thirst Knows No Season	1922	The Coca-Cola Company 2020[1]
Enjoy Thirst	1923	The Coca-Cola Company 2020[1]
Refresh Yourself	1924	The Coca-Cola Company 2020[1]
Six Million A Day	1925	The Coca-Cola Company 2020[1]
It Had to Be Good to Get Where It Is	1926	The Coca-Cola Company 2020[1]
Pure as Sunlight	1927	The Coca-Cola Company 2020[1]
Around the Corner from Everywhere	1927	The Coca-Cola Company 2020[1]
The Pause that Refreshes	1929	Zmuda 2009[2]
Ice Cold Sunshine	1932	The Coca-Cola Company 2020[1]
The Best Friend Thirst Ever Had	1938	The Coca-Cola Company 2020[1]
Thirst Asks Nothing More	1939	The Coca-Cola Company 2020[1]
Whoever You Are, Whatever You Do, Wherever You May Be, When You Think Refreshment Think of Ice Cold Coca-Cola	1939	The Coca-Cola Company 2020[1]

The Only Thing Like Coca-Cola is Coca-Cola Itself	1942	The Coca-Cola Company 2020[1]
Where There's Coke, There's Hospitality	1948	The Coca-Cola Company 2020[1]
Along the Highway to Anywhere	1949	The Coca-Cola Company 2020[1]
What You Want is a Coke	1952	The Coca-Cola Company 2020[1]
Coca-Cola… Makes Good Things Taste Better	1956	The Coca-Cola Company 2020[1]
Sign of Good Taste	1957	The Coca-Cola Company 2020[1]
The Cold, Crisp Taste of Coke	1958	The Coca-Cola Company 2020[1]
Be Really Refreshed	1959	The Coca-Cola Company 2020[1]
Things Go Better with Coke	1963	The Coca-Cola Company 2020[1]; Zmuda 2009[2]; Hanas et al. 2005[3]
It's the Real Thing	1969	The Coca-Cola Company 2020[1]; Zmuda 2009[2]
Look Up America	1975	The Coca-Cola Company 2020[1]
Coke Adds Life	1976	The Coca-Cola Company 2020[1]; Zmuda 2009[2]
Have a Coke and a Smile	1976	The Coca-Cola Company 2020[1]
Coke Is It!	1982	The Coca-Cola Company 2020[1]; Zmuda 2009[2]
We've Got the Taste for You	1985	The Coca-Cola Company 2020[1]
America's Real Choice	1985	The Coca-Cola Company 2020[1]
Red, White & You	1986	The Coca-Cola Company 2020[1]
Catch the Wave	1986	The Coca-Cola Company 2020[1]
When Coca-Cola is a Part of Your Life, You Can't Beat the Feeling	1987	The Coca-Cola Company 2020[1]
You Can't Beat the Feeling	1988	The Coca-Cola Company 2020[1]
Official Soft Drink of Summer	1989	The Coca-Cola Company 2020[1]
You Can't Beat the Real Thing	1990	The Coca-Cola Company 2020[1]
Always Coca-Cola	1993	The Coca-Cola Company 2020[1]; Zmuda 2009[2]; Garfield 1997[4]; Meyers 1998[5]; Kramer 1999[6]
Taste It All (Diet Coke)	1993	Sloan & Pollack 1997[7]; Kramer & Snyder 1999[8]
This is Refreshment (Diet Coke)	1994	Sloan & Pollack 1997[7]; Kramer & Snyder 1999[8]
Obey Your Thirst (Sprite)	1994	Gleason 1996[9]; Gleason 1996[10]; Garfield 2002[11]

The World Together. Always. (Olympic campaign)	1994	Davis & Magiera 1994[12]
Just for the Taste of It	1996	Sloan & Pollack 1997[7]; Kramer & Snyder 1999[8]; Kramer 1998[13]
Coca-Cola Incredible Summer	1997	Pollack 1997[14]; Zbar 1997[15]; Pollack 1997[16]
Get Caught Red Handed	1997	Gleason 1997[17]
Feed the Rush (Surge)	1997	Zbar 1997[15]
Fully Loaded Summer (Surge)	1997	Zbar 1997[15]
You are What You Drink (Diet Coke)	1998	Kramer[13]; Kramer & Snyder[8]; Pollack 1997[16]
Coke Card	1998	Fitzgerald 1998[18]; Kramer 1998[19]
Surge Around the World (Surge)	1998	Kramer 1998[20]
No Thirst is Safe (Citra grapefruit soda)	1998	Kramer 1998[21]; AdAge 1999[22]
Live Your Life (Diet Coke)	1999	Kramer & Snyder 1999[8]
Coca-Cola. Enjoy.	2000	The Coca-Cola Company 2020[1]; Snyder & Kramer 1999[21]; Garfield 2000[24]
It Could Be Your Next Coke	2000	MacArthur 2000[25]
Drink Sprite. Buy Rocketcash. Get What You Want.	2000	Chura & Petrecca 2000[26]
Life Tastes Good	2001	The Coca-Cola Company 2020[1]; AdAge 2001[27]; Chura et al. 2001[28]; Garfield 2001[29]; Chura & MacArthur 2001[30]; Chura 2003[31]; Case 2003[32]
Nature's A Mother. Drink to It (Mad River Teas, Coca-Cola & Nestle joint venture)	2002	Chura & Sanders 2002[33]
Coca-Cola…. Real	2003	The Coca-Cola Company 2020[1]; Chura 2003[31]; Case 2003[32]; Chura et al. 2002[34]; Chura 2003[35]; Chura 2003[36]; Garfield 2004[37]
Real, Make it Real	2005	The Coca-Cola Company 2020[1]
The Coke Side of Life / Welcome to the Coke Side of Life	2006	The Coca-Cola Company 2020[1]; Zmuda 2009[2]; Hein 2007[38]; Hein 2008[39]; Garfield 2009[40]; Diaz 2010[41]
Sip Stealing. Not a Felony in All 50 States.	2006	Garfield 2006[42]
Real Coca-Cola Taste with Zero Calories (Coke Zero)	2007	Hein 2008[39]
Great Taste Has its Benefits (Coke Zero Plus)	2007	Hein 2007[38]

Don't Dew It (Vault)	2009	Zmuda 2009[43]
Don't Settle for An Incomplete Sports Drink (Powerade)	2009	Zmuda 2009[44]
Open Happiness	2009	The Coca-Cola Company 2020[1]; Zmuda 2009[2]; Garfield 2009[40]; Hein 2009[45]; Hein 2009[46]; Diaz 2010[41]; Dorfman et al. 2012[47]; Schultz 2016[48]; Wheaton 2016[49]; Casaqui & Riegel 2016[50]; Gertner & Rifkin 2018[51]
Polar Bears Catch	2012	Econsultancy 2018[52]; The Coca-Cola Company 2012[53]
Liquid and Linked	2012	Econsultancy 2018[52]
Move to the Beat (London Olympics teen campaign)	2012	AdAge 2012[54]
Movement is Happiness	2013	Wood et al. 2020[55]
Mirage (2013 Super Bowl campaign)	2013	Zmuda 2014[56]
The Ahh Effect	2013	Econsultancy 2018[52]; Zmuda 2014[56]
Share a Coke	2014	Econsultancy 2018[52]; Zmuda 2014[56]; Gertner & Rifkin 2018[51]
It's Beautiful (2014 Super Bowl campaign)	2014	Zmuda 2014[56]
Get a Taste (Diet Coke)	2014	AdAge 2014[57]
Taste the Feeling	2016	The Coca-Cola Company 2020[1]; Schultz 2016[48]; Wheaton 2016[49]; Econsultancy 2018[52]; Schultz 2017[58]; Schultz 2018[59]; The Coca-Cola Company 2016[60]
#ThatsGold Rio campaign	2016	Econsultancy 2018[52]; Wood et al. 2020[55]
The Letter	2020	The Coca-Cola Company 2020[61]
Real Magic	2021	The Coca-Cola Company 2020[1]
PepsiCo Inc. (national) *(n = 84)*		
Exhilarating, Invigorating, Aids Digestion	1903	Zmuda 2009[2]; Sarosh n.d.[62]
Original Pure Food Drink	1907	Sarosh n.d.[62]
Delicious and Healthful	1908	Sarosh n.d.[62]
Drink Pepsi-Cola. It Will Satisfy You.	1913	Zmuda 2009[2]; Sarosh n.d.[62]
For All Thirsts – Pepsi-Cola	1915	Sarosh n.d.[62]
Pepsi-Cola – It Makes You Scintillate	1919	Sarosh n.d.[62]
Peps You Up!	1928	Sarosh n.d.[62]
Here's Health!	1929	Sarosh n.d.[62]

Sparkling, Delicious	1932	Sarosh n.d.[62]
It's the Best Cola Drink	1933	Sarosh n.d.[62]
Double Size	1934	Sarosh n.d.[62]
Refreshing and Healthful	1934	Sarosh n.d.[62]
Bigger Drink, Better Taste	1934	Sarosh n.d.[62]
Join the Swing to Pepsi-Cola	1938	Sarosh n.d.[62]
Twice as Much for a Nickel	1939	Zmuda 2009[2]; Sarosh n.d.[62]; G&M Distributors 2012[63]
It's A Great American Custom	1947	Sarosh n.d.[62]
Why Take Less When Pepsi's Best	1949	Sarosh n.d.[62]
More Bounce to the Ounce	1950	Sarosh n.d.[62]; G&M Distributors 2012[63]
Any Weather is Pepsi Weather	1950	G&M Distributors 2012[63]
The Light Refreshment	1954	Sarosh n.d.[62]
Refreshing Without Filling	1955	Sarosh n.d.[62]
Say Pepsi, Please	1957	G&M Distributors 2012[63]
Be Sociable / Be Sociable, Have a Pepsi / The Sociables	1958	Sarosh n.d.[62]; AdAge 1998[64]; Rosenshine 2008[65]
Now it's Pepsi for Those Who Think Young	1961	Zmuda 2009[2]; Hanas et al. 2005[3]; Sarosh n.d.[62]; G&M Distributors 2012[63]
Come Alive! You're in the Pepsi Generation.	1963	Zmuda 2009[2]; Hanas et al. 2005[3]; Sarosh n.d.[62]; G&M Distributors 2012[63]; AdAge 2008[66]
Taste that Beats the Others Cold. Pepsi Pours It On	1967	Sarosh n.d.[62]; G&M Distributors 2012[63]
You've Got a Lot to Live. Pepsi's Got a Lot to Give.	1969	Zmuda 2009[2]; Sarosh n.d.[62]; G&M Distributors 2012[63]; AdAge 1998[64]
Join the Pepsi People, Feelin' Free	1973	Zmuda 2009[2]; Sarosh n.d.[62]; G&M Distributors 2012[63]
Lipsmackin Thirst Quenchin Pepsi	1974	Sarosh n.d.[62]
Have a Pepsi Day	1976	Sarosh n.d.[62]
Catch that Pepsi Spirit	1979	Sarosh n.d.[62]; G&M Distributors 2012[63]
Pepsi's Got Your Taste for Life	1981	Sarosh n.d.[62]; G&M Distributors 2012[63]
Pepsi Now! Take the Challenge	1983	Sarosh n.d.[62]; G&M Distributors 2012[63]
Pepsi. The Choice of a New Generation	1984	Zmuda 2009[2]; Sarosh n.d.[62]; G&M Distributors 2012[63]; McDonough 1998[67]; Davisson & Booth 2010[68]; Love 2005[69]

Pepsi. A Generation Ahead	1989	G&M Distributors 2012[63]
Gotta Have It / Chill Out	1991	Sarosh n.d.[62]; G&M Distributors 2012[63]
The Choice is Yours	1992	Sarosh n.d.[62]; G&M Distributors 2012[63]
Be Young, Have Fun, Drink Pepsi	1993	Sarosh n.d.[62]; G&M Distributors 2012[63]
Right Now	1993	G&M Distributors 2012[63]
Double Dutch Bus	1994	G&M Distributors 2012[63]
Do the Dew (Mountain Dew)	1995	Howard 2000[70]
Nothing Else is a Pepsi	1995	Zmuda 2009[2]; Sarosh n.d.[62]; G&M Distributors 2012[63]
Drink Pepsi. Get Stuff.	1995	Pollack 1997[14]; Gleason 1997[17]; Sarosh n.d.[62]; G&M Distributors 2012[63]; Fitzgerald 1997[71]; Morales et al. 2000[72]; Zmuda 2008[73]
Change the Script	1996	Sarosh n.d.[62]; G&M Distributors 2012[63]
Generation Next	1997	Kramer 1999[6]; Sarosh n.d.[62]; G&M Distributors 2012[63]
This is Diet? (Diet Pepsi)	1997	Sloan & Pollack 1997[7]; Kramer 1998[13]; AdAge 1997[74]
It's the Cola	1998	Sarosh n.d.[62]; G&M Distributors 2012[63]
For Those Who Think Young / The Joy of Pepsi-Cola	1999	Kramer 1999[6]; Sarosh n.d.[62]; G&M Distributors 2012[63]; Garfield 1999[75]
I Love My Mug (Mug root beer)	1999	Kramer 1999[76]
Ask for More	1999	Sarosh n.d.[62]
Too Good to Be One Calorie. But It Is. (Pepsi One)	2000	Howard 2000[70]
Choose Your Music	2000	MacArthur 2000[25]
Share the Joy with Music	2000	Thompson 2000[77]
The Joy of Pepsi	2001	Zmuda 2009[2]; Sarosh n.d.[62]
Mountain Dew Pirate Radio	2001	Fitzgerald 2001[78]
Think Young Drink Young	2002	Sarosh n.d.[62]
Pepsi – It's the Cola	2003	Sarosh n.d.[62]; G&M Distributors 2012[63]; Garfield 2003[79]; Geier et al. 2004[80]; MacArthur 2006[81]
Dare for More	2003	Sarosh n.d.[62]; G&M Distributors 2012[63]
Why You Doggin' Me / Taste the One That's Forever Young	2006	Sarosh n.d.[62]; G&M Distributors 2012[63]
Catch that Pepsi Spirit	2006	MacArthur 2006[81]
More Happy	2007	Hein 2007[38]; Sarosh n.d.[62]; G&M Distributors 2012[63]; Garfield 2007[82]

More Cola Taste (Diet Pepsi)	2007	Garfield 2007[83]
Wake Up People! (Diet Pepsi Max)	2007	MacArthur 2007[84]; Zmuda 2010[85]
Pepsi is #1	2008	Sarosh n.d.[62]; G&M Distributors 2012[63]
Something for Everyone	2008	Sarosh n.d.[62]; G&M Distributors 2012[63]
Every Sip Brings You Closer (campaign for PepsiStuff.com)	2008	Hein 2008[39]
Yes You Can	2009	Davisson & Booth 2010[68]
Zero Calories, Maximum Taste (Pepsi Max)	2009	Zmuda 2010[85]
Refresh Everything / Every Generation Refreshes the World	2009	Hein 2009[45]; Sarosh n.d.[62]; G&M Distributors 2012[63]
Every Pepsi Refreshes the World	2010	Zmuda 2009[2]; Sarosh n.d.[62]; G&M Distributors 2012[63]
Summer Time is Pepsi Time	2011	Sarosh n.d.[62]; G&M Distributors 2012[63]
Born in the Carolinas	2011	Sarosh n.d.[62]; G&M Distributors 2012[63]
Where There’s Pepsi, There’s Music	2012	Sarosh n.d.[62]; G&M Distributors 2012[63]
Live for Now	2012	Sarosh n.d.[62]; G&M Distributors 2012[63]; Lafayette 2013[86]
Change the Game	2012	Sarosh n.d.[62]; G&M Distributors 2012[63]
The Best Drink Created Worldwide	2012	Sarosh n.d.[62]; G&M Distributors 2012[63]
This is How We Dew (Mountain Dew)	2012	Zmuda 2012[87]
Win from Within (Gatorade)	2012	Zmuda 2012[88]
Polar Bowl	2012	Zmuda 2014[52]
Make Interesting Happen (Sierra Mist)	2014	AdAge 2014[57]
Out of the Blue	2015	Sarosh n.d.[62]
Pepsi Generations	2018	BevNet 2018[89]
That’s What I Like	2020	Steinberg 2020[90]
Greatest of All Time (GOAT) Camp	2020	PRNewswire 2020[91]
Branded Juice Campaigns (national) *(n = 9)*		
Memories (Welch’s)	1994	Hughes 2000[92]
Bite into It (Minute Maid orange juice, Coca-Cola)	1997	Pollack 1996[93]; Pollack & Petrecca 1997[94]
100% Juice (Northland cranberry juice)	1997	Schwirtz 1999[95]

We Only Pick the Best Fruit (Ocean Spray)	1998	Kramer 1998[96]
Squeeze the Day (Minute Maid orange juice, Coca-Cola)	1999	Kramer 1999[97]
Introducing Simply Orange…100% Unfooled Around With (Minute Maid orange juice, Coca-Cola)	2001	AdAge 2001[98]
Squeeze, It's a Natural (Tropicana, PepsiCo)	2009	Lee et al. 2010[99]
Wake Up Your MMOJO (Minute Maid orange juice, Coca-Cola)	2011	Zmuda 2011[100]
Tap into Nature (Tropicana, PepsiCo)	2012	Zmuda 2012[101]
Branded Coffee Campaigns (national) *(n = 2)*		
Sophisticated Taste (Taster's Choice instant coffee, Nescafe, Nestle)	1990	Kretchmer 2004[102]
The Coffee for Intense Taste (Nescafe coffee, Nestle)	1997	Kramer & Cuneo 1997[103]
Branded Water Campaigns (national) *(n = 5)*		
It's a Brett Favre Thing (Real Pure water & sports drinks)	2000	Thompson 2000[104]
L'Original (Evian)	2000	Ad Age 2004[105]
We Promise Nothing (Aquafina, PepsiCo)	2002	Ad Age 2002[106]
Evian: Your Natural Source of Youth	2004	Ad Age 2004[105]
I Wanna Hashtag #Liveyoung	2018	Beverage Industry 2018[107]
Branded Non-Dairy, Plant-Based Milk Campaigns (national) *(n = 3)*		
Get Your Soy with Silk	2000	Chura 2000[108]
Silk: Milk of the Land (Danone North America, almond milk)	2021	Cooper 2021[109]; Hiebert 2021[110]
Wow No Cow (Oatly, oat milk)	2021	Wohl 2021[111]
**2. CSR, public relations and cause marketing campaigns** (*n* = 16)		

The American Beverage Association national) *(n = 1)*		
The Mixify Campaign / The Balance Calorie Initiative (Los Angeles, CA; Little Rock, AR; Mississippi Delta; Montgomery, AL; and NYC, NY)	2014	Cohen et al. 2018[112]; Cohen et al. 2020[113]
The Coca-Cola Company national) *(n = 5)*		
Live Positively (CSR campaign)	2010	Dorfman et al. 2012[47]
Coca-Cola Every Bottle has a Story (sustainability campaign)	2011	Casaqui & Riegel 2016[50]
The Great Meal/Together Tastes Better	2020	The Coca-Cola Company 2020[61]; The Coca-Cola Company 2020[114]
Together We Must	2020	The Coca-Cola Company 2020[115]
Refreshing the World and Making a Difference	2021	The Coca-Cola Company 2021[116]
PepsiCo, Inc. national) *(n = 7)*		
Helping Children Get Clean Water (Ethos water, Starbucks/PepsiCo)	2007	Brei & Boehm 2011[117]
Pepsi Refresh Project (CSR)	2010	Dorfman et al. 2012[47]; Schultz 2016[48]; Steinberg & Zmuda 2009[118]; Zmuda & Patel 2010[119]; Iezzi 2010[120]; Zmuda 2010[121]; Crain 2010[122]
Pepsi We Inspire	2010	Applebaum 2010[123]
Black Lives Matter	2017	Victor 2017[124]
Black Art Rising (LIFEWTR)	2020	LIFEWTR n.d.[125]
Food for Good	2020	PepsiCo 2021[126]
Life Unseen	2021	Yodice 2021[127]
Danone North America national) *(n = 1)*		
Drink 1, Give 10 / 1L=10L for Africa (Volvic water, Danone)	2008	Brei & Boehm 2011[117]
Nestlé North America national) *(n = 2)*		
Nestlé Waters Challenge	2019	Nestle 2021[128]
Nestlé Pure Life	2019	NewsDesk 2017[129]
**3. Social marketing campaigns** (national) (*n* = 20)		

Fluid Cow's Milk Campaigns *(n = 11)*		
Every Body Needs Milk (California Milk Producers Advisory Board)	1970s	Werner & Cohen 1980[130]
Milk. It Does a Body Good (America's Dairy Farmers & National Dairy Board)	Late 1980s	Keegan 2016[131]
Lowfat Milk Campaign (New York City, NY)	1990	Wechsler & Wernick 1992[132]
1% or Less (West Virginia, California, Hawaii and Oklahoma)	1995	Reger et al. 1998[133]; Reger et al. 1999[134]; Reger et al. 2000[135]; Booth-Butterfield, et al. 2004[136]; Wootan et al. 2005[137]; Maddock et al. 2007[138]; Hinkle et al. 2008[139]
Got Milk? / Toma Leche? (Milk Processor Education Program, nationwide)	1995	Cuneo 1995[140]; Gleason & Kelly 1996[141]; Pollack 1996[142]; Pollack et al. 1998[143]; Pollack & Cuneo 1999[144]; Thompson 2000[145]; Thompson 2000[146]; Fitzgerald 2002[147]; Pszczola 2003[148]; Manning & Keller 2004[149]; Van der Waldt et a. 2007[150]; AdAge 2007[151]; Miles 2007[152]; AdAge 2015[153]; Zhou et al. 2019[154]; Zhou et al. 2020[155]; Zhou & Kraak 2021[156]; Holt 2002[157]; Manning 2016[158]; Wheaton 2014[159]
Milk Mustache (Milk Processor Education Program, nationwide)	1995	Gleason & Kelly 1996[141]; Pollack 1996[142]; Pollack et al. 1998[143]; Pollack & Cuneo 1999[144]; Thompson 2000[145]; Zhou et al. 2019[154]; Zhou et al. 2020[155]; Ad Age 1997[160]; Pollack 1997[161]; Pollack & Kerwin 1997[162]; Pollack 1998[163]; Fitzgerald 1998[164]; Pollack & Teinowitz 1998[165]; Schulberg 1998[166]; Chung-kue & McDonald 2002[167]
Milk Made Better (Suiza Foods – dairy processor & distributor)	2000	Thompson 2000[145]
White Gold (California Milk Processor Board)	2008	Stanley 2009[168]
Milk Life (Milk Processor Education Program, National)	2014	Wheaton 2014[159]
Choose 1% Milk (Oklahoma)	2014	John et al. 2019[169]; Robert et al. 2017[170]
You're Gonna Need Milk with That. Got Milk? (Milk Processor Education Program, Re-launched)	2020	America's Milk Companies 2021[171]
Water and Juice Promotion Campaigns (national) *(n = 9)*		

Drink Up! (Partnership for Healthier America)	2013-2016	Partnership for a Healthier America 2021[172]; Gertner & Rifkin 2018[51]; Zhou et al. 2019[154]; Zhou et al. 2020[155]; Zhou & Kraak 2021[156]
Live Sugarfreed (rural KY, TN, VA)	2015	Farley et al. 2017[173]; Falbe & Madsen 2017[174]
One Less Challenge (Delaware)	2015	Peterson 2016[175]
Kim and Pura (New York City, NY)	2016	Sisti et al. 2021[176]
NJ Live Sugarfreed	2017	Bonnevie et al. 2020[177]
Sip Smarter – Every Day Begins with a Sip (Juice Products Association, national)	2018	Juice Products Association 2020[178]
Choose Water Not Sugary Drinks! (Healthy Berkeley Partnership, CA)	2020	Healthy Berkeley Partnerships 2021[179]
Skip the Sugar, Choose Water (Albany, CA)	2020	City of Albany California 2020[180]
Be Ready. Be Hydrated. Drink Water (Seattle, WA)	2020	Be Ready Be Hydrated n.d.[181]
**4. Public information, awareness, education or health promotion campaigns** (*n* = 27)		
Get Coke Out of Seattle Schools (Seattle, WA)	1996	Freudenberg et al. 2009[182]
Rethink Your Drink (San Francisco, CA)	2008	CDC 2020[183]
Are You Pouring on the Pounds? (New York City, NY)	2009	Bleakley et al. 2015[184]; Dillard et al. 2018[185]; James et al. 2020[186]; Kansagra et al. 2015[187]; Popkin & Hawkes 2016[188]; Sisti et al. 2021[176]; Garfield 2009[189]
Drinks Destroy Teeth (Indiana Dental Association, statewide)	2010	Solana 2019[190]
Rethink Your Drink (Cook County, IL)	2010	Bleakley et al. 2015[184]; Cook County Government 2012[191]
Are You Pouring on the Pounds (San Francisco, CA)	2010	Samuels & Associates 2010[192]
FatSmack (Boston, MA)	2011	Te et al. 2019[193]; Boston Public Health Commission 2011[194]
Life's Sweeter with Fewer Sugary Drinks (Boston, MA; Los Angeles, CA;	2011	CSPI 2011[195]

Philadelphia, PA; San Antonio, TX and Seattle, WA)		
Sugar Pack (Los Angeles County, CA)	2011	Popkin & Hawkes 2016[188]; Barragan et al. 2014[196]; Robles et al. 2015[197]; Gase et al. 2014[198]
Sugar Bites (Contra Costa County, CA)	2011	Te et al. 2019[193]; First Five Contra n.d.[199]
It Starts Here (Portland & Multnomah County, OR)	2011	Boles et al. 2014[200]; Bleakley et al. 2015[184]
Get Healthy Philly (Philadelphia, PA)	2011	Bleakley et al. 2018[201]
Howard County Unsweetened (Howard County, MD)	2012	Falbe & Madsen 2017[174]; Farley et al. 2017[173]; Schwartz et al. 2017[202]
Rev Your Bev (Virginia statewide)	2013	Te et al. 2019[193]
Sugar Smarts / Azucar Sabia (Boston, MA)	2013	Aguilar-Santos 2015[203]
Drink Yourself Sick / Your Kids Could Be Drinking Themselves Sick (New York City, NY)	2013	Sisti et al. 2021[176]
Rethink your Drink (San Diego, CA)	2012	Te et al. 2019[193]; Hartigan et al. 2017[204]
Sounds Healthy (New York City, NY)	2013	Sisti et al. 2021[176]
Cavities Get Around (Delta Dental of Colorado Foundation, CO)	2014	Chavez 2014[205]; Hornsby et al. 2017[206]
Rethink Your Drink (CDC, in cities nationwide)	2015	James et al. 2020[186]; Minneapolis Health Department 2020[207]; California Department of Health 2020[208]; Maghrabi & Terry 2021[209]
Choose Water (Los Angeles, CA)	2015	Caldwell et al. 2020[210]
Drink NYC Tap Water (New York City, NY)	2016	Sisti et al. 2021[176]
Sour Side of Sweet (New York City, NY)	2017	NYC Department of Health 2017[211]
Hidden Sugar (Denver, CO)	2017	Healthy Beverage Partnership 2017[212]
Rethink Your Drink (Arkansas statewide)	2017	Healthy Active Arkansas 2017[213]

Healthy for Good Sip Smarter (American Heart Association)	2018	American Heart Association 2018[214]
Healthy Drinks Healthy Kids (RWJF Heathy Eating Research, national)	2020	Robert Wood Johnson Foundation n.d.[215]
**5. Media advocacy or countermarketing campaigns** (*n* = 10)		
Global Dump Soft Drinks (national and international)	2007	Global Dump Soft Drinks Campaign 2008[216]; Global Dump Soft Drinks Campaign 2008[217]; CSPI 2008[218]
Dunk the Junk (San Francisco, CA)	2011	Te et al. 2019[193]; Dunk the Junk n.d.[219]
The Real Bears (CSPI, national)	2012	Te et al. 2019[193]; CSPI 2012[220]
Soda Sucks (California)	2012	Te et al. 2019[193]; Allen Meyer Design n.d.[221]
Kick the Can (California and national)	2012	Te et al. 2019[193]; Marriott & Dillard 2020[222]
Coming Together: Translated (CSPI)	2013	CSPI n.d.[223]
The Bigger Picture (San Francisco, CA)	2013	Te et al. 2019[193]; University of California San Francisco n.d.[224]
'Share a Coke' With Obesity (Center for Science in the Public Interest)	2015	Gertner & Rifkin 2018[51]; CSPI n.d.[223]
Change the Tune (CSPI)	2015	CSPI n.d.[223]
Open Truth Now (San Francisco, CA)	2015	Te et al. 2019[193]; Open Truth n.d.[225]
**6. Political or public policy campaigns** (*n* = 23)		
Richmond Fit for Life (Richmond, CA)	2012	Marriott & Dillard 2020[222]
No on N (Richmond, CA)	2012	Marriott & Dillard 2020[222]
Healthy Diné Nation Act (Navajo Nation)	2014	Yazzie et al. 2020[226]
Choose Health SF (San Francisco, CA)	2014	Somji et al. 2016[227]
No SF Beverage Tax / No on E (San Francisco, CA)	2014	Marriott & Dillard 2020[222]; Somji et al. 2016[227]
No Berkeley Beverage Tax (ABA anti-tax, Berkeley, CA)	2014	Marriott & Dillard 2020[222]; Somji et al. 2016[227]; Healthy Food America 2018[230]
Vote Yes on Measure D / Berkeley vs. Big Soda (pro-tax, Berkeley, CA)	2014	Marriott & Dillard 2020[222]; Somji et al. 2016[227]; Altman et al. 2021[228]; Falbe et al. 2016[229]; Healthy Food America 2018[230]

Vote Yes on Measure HH to Protect Our Children's Health / Oakland vs. Big Soda (pro-tax, Oakland, CA)	2016	Falbe et al. 2020[231]; Healthy Food America 2018[232]
No Oakland Grocery Tax and No on HH (Oakland, CA, ABA-supported, anti-tax)	2016	Marriott & Dillard 2020[222]; Healthy Food America[232]; Asada et al. 2021[233]; Asada et al. 2021[234]
Vote Yes on Proposition V (pro-tax, San Francisco, CA)	2016	Healthy Food America 2018[235]
No on V / Enough is Enough: Keep Our Groceries Tax Free (anti-tax, San Francisco, CA)	2016	Marriott & Dillard 2020[222]; Healthy Food America 2018[235]
Yes on O1 (Albany, CA)	2016	Healthy Food America 2018[236]
No on O1 (Albany, CA)	2016	Healthy Food America 2018[236]
Vote Yes on Soda Because Our Kids are Worth It! (Philadelphia, PA)	2016	Healthy Food America 2018[237]; Lawman et al. 2020[238]; Bleich et al. 2020[239]
No Philly Grocery Tax	2016	Healthy Food America 2018[237]; Lawman et al. 2020[238]; Bleich et al. 2020[239]
Healthy Boulder Kids Campaign (Boulder, CO)	2016	Healthy Food America 2018[240]
Pre-K for Santa Fe (pro-tax, Santa Fe, NM)	2017	Marriott & Dillard 2020[222]
Better Way for Santa Fe & Pre-K (anti-tax, Santa Fe, NM)	2017	Marriott & Dillard 2020[222]
Seattle Healthy Kids Coalition (Seattle, WA)	2018	Healthy Food America 2018[241]; Krieger et al. 2021[242]
Keep Seattle Liveable for All (Seattle, WA)	2017	Healthy Food America[241]
Yes! to Affordable Groceries (anti-tax, Washington, WA)	2017	Falbe et al. 2021[243]; Crosbie et al. 2021[244]
Vote Yes on Measure 103 to Keep Our Groceries Tax Free (anti-tax, Oregon)	2017	Falbe et al. 2021[243]; Crosbie et al. 2021[244]
Keep Groceries Affordable Act of 2018	2018	Crosbie et al. 2021[244]

References

1. The Coca-Cola Company. *History of Coca-Cola Advertising Slogans.* 2020. Available online: https://www.coca-colacompany.com/news/history-of-coca-cola-advertising-slogans (accessed on 1 October 2021).
2. Zmuda N. Pepsi, Coke try to out-do each other with rays of sunshine. *AdAge*. 2009;80(2):6. Available online: https://adage.com/article/news/pepsi-coke-outdo-rays-sunshine/133859 (accessed on 1 October 2021).
3. Hanas J, MacArthur K, Thomaselli R, Thompson S. 75 years of ideas. *AdAge*. 21 February 2005.
4. Garfield B. Coca-Cola Classic loses its former edge. *AdAge*. 1997;68(18):69.
5. Meyers CB. Global marketing and the New Hollywood: the making of the 'Always Coca-Cola' campaign. *Media International Australia*. 1998;86:27-37. doi: 10.1177/1329878X9808600105.
6. Kramer L. New Pepsi-cola spots a big hit with bottlers. *AdAge*. 8 March 1999;70(10):4–59.
7. Sloan P, Pollack J. Diet Coke works up new theme: $40 mil effort uses humor to show how brand boosts sex appeal. *AdAge*. 12 May 1997;68(19):1-86. Available online: https://adage.com/article/news/diet-coke-works-theme-40-mil-effort-humor-show-brand-boosts-sex-appeal/72135 (accessed on 1 October 2021).
8. Kramer L, Snyder B. Low-calorie colas to heavy up on ads. *AdAge*. 4 January 1999;70(1):3–28. Available online: https://adage.com/article/news/low-calorie-colas-heavy-ads/31295 (accessed on 1 October 2021).
9. Gleason M. Sprite Chris Lowe. *AdAge*. 24 June 1996;67(26):S28.
10. Gleason M. Sprite is riding global ad effort to no. 4 status. *AdAge*. 18 November 1996;67(47):30. Available online: https://adage.com/article/news/sprite-riding-global-ad-effort-4-status-simple-concept-lampoons-hype-strikes-chord-kids-world/75591 (accessed on 1 October 2021).
11. Garfield B. Sprite's latest won't help it escape confines of Dorkville. *AgAge*. 5 August 2002;73(31):41. Available online: https://adage.com/article/ad-review/sprite-s-latest-escape-confines-dorkville/51620 (accessed on 1 October 2021).
12. Davis RA, Magiera M. Coke takes bottle outdoors in big way. *AdAge*. 2 May 1994;65(19):14. Available online: https://adage.com/article/news/coke-takes-bottle-outdoors-big/87335 (accessed on 1 October 2021).
13. Kramer L. Diet Coke steers down 2 paths with dueling ad campaigns. *AdAge*. 26 January 1998;69(4):3–51. Available online: https://adage.com/article/news/diet-coke-steers-2-paths-dueling-ad-campaigns-wieden-ads-offer-real-life-scenarios-tagline/67018 (accessed on 1 October 2021).
14. Pollack J. Huge promotions key as soft drinks enter summer. *AdAge*. 12 May 1997;68(19):89. Available online: https://adage.com/article/news/huge-promotions-key-soft-drinks-enter-summer-pepsi-teams-sports-stars-coke-s-effort-offers-debit-cards/72090 (accessed on 1 October 2021).
15. Zbar JD. Surge. AdAge. 30 June 1997;68(26)s35-s35.
16. Pollack J. Burnett gets new boost from Coca-Cola. *AdAge.* 1997;68(20):8. Available online: https://adage.com/article/news/burnett-boost-coca-cola-incredible-summer-promo-15-mil-ads/72033 (accessed on 1 October 2021).

17. Gleason M. Coke creates prize-laden ‘Red Crew’. *AdAge*. 1997;68(1):2-2. Available online: https://adage.com/article/news/coke-creates-prize-laden-red-crew/75741 (accessed on 1 October 2021).
18. Fitzgerald K. Coke scans campus. *AdAge*. 1998;69(13):46-48. Available online: https://adage.com/article/news/coke-scans-campus-big-card-promo-instant-prizes-bar-code-device/66396 (accessed on 1 October 2021).
19. Kramer L. Coca-Cola to boost local efforts for Classic via revamp. *AdAge*. 1998;69(14):3–47. Available online: https://adage.com/article/news/coca-cola-boost-local-efforts-classic-revamp/31070 (accessed on 1 October 2021).
20. Kramer L. Coca-Cola backing surge with promo offering trips. *AdAge*. 1998;69(15):38. Available online: https://adage.com/article/news/coca-cola-backing-surge-promo-offering-trips-mountain-dew-links-pepsi-s-pop-culture-push/66183 (accessed on 1 October 2021).
21. Kramer L. Coke’s Citra sets ambitious rollout plan. *AdAge*. 1998;69(4):1-52. Available online: https://adage.com/article/news/coke-s-citra-sets-ambitious-rollout-plan/31004 (accessed on 1 October 2021).
22. Gangster with a thirst. *AdAge*. 8 March 1999;70(10):58. Available online: https://adage.com/article/news/gangster-a-thirst/63284 (accessed on 1 October 2021).
23. Snyder B, Kramer L. Burnett, Edge craft Coke creative. *AdAge*. 1999;70(51):1–92. Available online: https://adage.com/article/news/burnett-edge-craft-coke-creative/31582 (accessed on 1 October 2021).
24. Garfield B. Coke’s giant step backwards cans superior ‘Always’ theme. *AdAge*. 2000;71(3):53–53. Available online: https://adage.com/article/snapshot/coke-s-giant-step-backwards-cans-superior-theme/59781 (accessed on 1 October 2021).
25. MacArthur K. Topless summer. *AdAge*. 8 May 2000;71(20):36-36. Available online: https://adage.com/node/1686541/printable/print (accessed on 1 October 2021).
26. Chura H, Petrecca L. Coca-Cola heads online to win teens for Sprite. *AdAge.* 2000;71(36):8. Available online: https://adage.com/article/news/coca-cola-heads-online-win-teens-sprite/57161 (accessed on 1 October 2021).
27. For the record. AdAge. 23 April 2001;72(17):36.
28. Chura H, Linnett R, Hughes LQ. Coca-Cola readies global assault. *AdAge.* 2 April 2001;72(14):1-34. Available online: https://adage.com/article/news/coca-cola-readies-global-assault/55142 (accessed on 1 October 2021).
29. Garfield B. World’s best-known product goes with generic campaign. *AdAge.* 23 April 2001;72(17):41. Available online: https://adage.com/article/news/world-s-product-generic-campaign/54932 (accessed on 1 October 2021).
30. Chura H, MacArthur K. Life doesn’t taste good at Coca-Cola. *AdAge.* 16 July 2001;72(29):1-44.
31. Chura H. Coke strategy shift bad news for Big 3. *AdAge.* 13 January 2003;74(2):1–53. Available online: https://adage.com/article/news/coke-strategy-shift-bad-news-big-3/50548 (accessed on 1 October 2021).
32. Case T. Universal McCann. Mediaweek. 23 June 2003;13(25):SR28.
33. Chura H, Sanders L. Coke/Nestle venture tests Mad River ads. *AdAge.* 3 June 2002;73(22):4–54. Available online: https://adage.com/article/news/alternative-beverage-battle-coke-nestle-venture-tests-mad-river-ads/52071 (accessed on 1 October 2021).

34. Chura H, MacArthur K, Sanders L, Thomaselli R. WPP takes lead on new Coke effort. *AdAge.* 2002;73(45):3–45. Available online: https://adage.com/article/news/wpp-takes-lead-coke-effort/50892 (accessed on 1 October 2021).
35. Chura H. Coke cites IPG but Berlin is real thing. *AdAge.* 2003;74(2):52–52. Available online: https://adage.com/article/news/coke-cites-ipg-berlin-real-thing/50487 (accessed on 1 October 2021).
36. Chura H. Coke Classic to anoint Berlin. *AdAge.* 2003;74(5):1–29. Available online: https://adage.com/article/news/coke-classic-anoint-berlin/50329 (accessed on 1 October 2021).
37. Garfield B. Coke finally gets Real right: cool kids deliver the message. *AdAge*. 6 September 2004;75(36):25.
38. Hein K. Beverages. *MediaWeek*. 30 April 2007;17(18):SR12.
39. Hein K. Beverages. *MediaWeek*. 28 April 2008;18(17):SR10.
40. Garfield B. Coke's 'Open Happiness' narrowly misses the mark. *AdAge*. 26 January 2009;80(3):39. Available online: https://adage.com/article/ad-review/garfield-coke-s-open-happiness-narrowly-misses-mark/134084 (accessed on 1 October 2021).
41. Diaz AC. Definition 6. *AdAge.* 26 July 2010;81(28):9-9.
42. Garfield B. Coca-Cola gets its ads right—finally. *AdAge.* 2006;77(2):1–37. Available online: https://adage.com/node/2055601/printable/print (accessed on 1 October 2021).
43. Zmuda N. Coke: buy 1 rival, get our brand free. *AdAge*. 2009;80(9):1–19. Available online: https://adage.com/article/news/coke-s-vault-takes-pepsi-s-mtn-dew-coupon-giveaway/135104 (accessed on 1 October 2021).
44. Zmuda N. Gator baiter: Powerade jabs at powerhouse. *AdAge.* 2009;80(10):3–29. Available online: https://adage.com/article/news/gator-baiter-powerade-jabs-powerhouse-gatorade/135436 (accessed on 1 October 2021).
45. Hein K. Beverages. *MediaWeek*. 2009;19(17):AM5-AM5.
46. Hein K. Noncarbonated losing fizz. *MediaWeek.* 2009;19(23):4.
47. Dorfman L, Cheyne A, Friedman LC, Wadud A, Gottlieb M. Soda and tobacco industry corporate social responsibility campaigns: how do they compare? *PloS Med.* 2012;9:e1001241. https://doi.org/10.1371/journal.pmed.1001241.
48. Schultz EJ. Coke could lead the way to more product-focused ads. *AdAge*. 2016;87(2):0002. Available online: https://adage.com/article/print-edition/coke-lead-product-focused-ads/302311 (accessed on 1 October 2021).
49. Wheaton K. Why I'm looking forward to having a Coke and a smile and some product-centric marketing. *AdAge*. 25 January 2016;87(2):0064-0064. Available online: https://adage.com/article/ken-wheaton/a-coke-a-smile-product-centric-marketing/302312 (accessed on 1 October 2021).
50. Casaqui V, Riegel V. Management of happiness, production of affects and the spirit of capitalism: international narratives of transformation from Coca-Cola brand. *J Int Commun*. August 2016;22(2):293–314. doi: 10.1080/13216597.2016.1194304
51. Gertner D, Rifkin L. Coca-Cola and the fight against the global obesity epidemic. *Thunderbird International Business Review*. 2018;60(2):161–173. doi: 10.1002/tie.21888.
52. 10 inspiring digital marketing campaigns from Coca-Cola. *Econsultancy.* 23 November 2018. Available online: https://econsultancy.com/digital-marketing-campaigns-coca-cola/(accessed on 1 October 2021).

53. The Coca-Cola Company. Live from the artic: Coca-Cola animated polar bears to “catch” the big game on Feb. 5. 26 January 2012. Available online: https://www.coca-colacompany.com/press-releases/coca-cola-animated-polar-bears-to-catch-the-big-game (accessed on 1 October 2021).
54. Marketers bow Olympic-size pushes. *AdAge.* 2012;83(27):3. Available online: https://adage.com/article/global-news/expect-major-marketers-olympic-pushes/235872 (accessed on 1 October 2021).
55. Wood B, Ruskin G, Sacks G. Targeting children and their mothers, building allies and marginalising opposition: an analysis of two Coca-Cola public relations requests for proposals. *Int J Environ Res Public Health*. 2020;17(1).https://doi.org/10.3390/ijerph17010012.
56. Zmuda N. Top digital marketers. *AdAge*. 2014;85(19):32.
57. Marketing digest. *AdAge*. 2014;85(20):4.
58. Schultz EJ. The soft sell gets harder. *AdAge*. 2017;88(12):48.
59. Schultz EJ. Marketer a-list 2018: marketing makeover puts Diet Coke back on growth track. *AdAge.* 2018;89(24):18. Available online: https://adage.com/article/cmo-strategy/marketer-a-list-coca-cola/315796 (accessed on 1 October 2021).
60. The Coca-Cola Company. “Taste the Feeling” by Conrad Sewell [video]. 19 January 2016. Available online: https://www.youtube.com/watch?v=xa6mLZf5HVw (accessed on 1 October 2021).
61. The Coca-Cola Company. Coca-Cola invites world to ‘give something only you can give’ in 2020 holiday campaign. 11 November 2020. Available online: https://www.coca-colacompany.com/news/2020-holiday-campaign (accessed on 1 October 2021).
62. Sarosh S. How Pepsi slogans connect with generations over the years. *Advergize*. n.d. Available online: https://www.advergize.com/marketing/history-of-pepsi-slogans-connect-generations-years/ (accessed on 1 October 2021).
63. G&M Distributors. Pepsi slogans and logos throughout the years. 11 December 2012. Available online: https://gmdist.com/blog/pepsi-slogans-and-logos-throughout-the-years (accessed on 1 October 2021).
64. A selection of Pepsi creative work. *AdAge*. 20 July 1998;69(29):c16–c18. Available online: https://adage.com/article/news/a-selection-pepsi-creative-work/65032 (accessed on 1 October 2021).
65. Rosenshine A. Evolution of a revolution. *AdAge*. 23 June 2008;79(25):c10.
66. Launching a legend: ‘Pepsi Generation’. *AdAge*. 26 July 1999;70(31):47. Available online: https://adage.com/article/news/launching-a-legend-pepsi-generation/61675 (accessed on 1 October 2021).
67. McDonough J. Pepsi advertising, viewed from agency perspective. *AdAge*. 1998;69(29):c10–c14.
68. Davisson A, Booth P. Intertextuality, parody, and polyphony in Pepsi’s 2009 presidential inauguration campaign. *J Visual Literacy*. Spring 2010;29(1):68-87. https://doi.org/10.1080/23796529.2010.11674674.
69. Love J. From cautionary chart-topper to friendly beverage anthem: Michael Jackson’s “Billie Jean” and Pepsi’s “Choice of a New Generation” television campaign. *Journal Society for American Music*. 2005;9(2):178–203. https://doi.org/10.1017/S175219631500005X.
70. Howard T. Soft drinks. *MediaWeek*. 2000;10(17):U38.

71. Fitzgerald K. Pepsi stuff. *AdAge*. 1997;68(26):s22. Available online: https://adage.com/article/news/marketing-100-pepsi-stuff-brian-swette/71524 (accessed on 1 October 2021).
72. Morales AC, Cava A, Sacasas R, Eds. Marketing and the law. *J Acad Mark Sci*. 2000;28(2):316–320. https://doi.org/10.1177/0092070300282012.
73. Zmuda N. Pepsi hopes to make killing with monster promo. *AdAge*. 2008;79(3):3–33. Available online: https://adage.com/article/news/pepsi-hopes-make-killing-monster-promo/123223 (accessed on 1 October 2021).
74. Low-cal relief for stressful situations. *AdAge*. 1997;68(16):3. Available online: https://adage.com/article/news/hot-spot-diet-pepsi-low-cal-relief-stressful-situations/68540 (accessed on 1 October 2021).
75. Garfield B. Pepsi has 'Joy' but no strategy; Lite has 'Taste' but little charm. *AdAge*. 1999;70(10):59. Available online: https://adage.com/article/news/pepsi-joy-strategy-lite-taste-charm/15672 (accessed on 1 October 2021).
76. Kramer L. Mug pours irreverent humor for older target. *AdAge*. 1999;70(23):71. Available online: https://adage.com/article/news/mug-pours-irreverent-humor-older-target-root-beer-ads-bow-simpsons/62264 (accessed on 1 October 2021).
77. Thompson S. Pepsi hits high note with schools. *AdAge*. 2000;71(42):30. Available online: https://adage.com/article/news/pepsi-hits-high-note-schools/56668 (accessed on 1 October 2021).
78. Fitzgerald K. Viral marketing breaks through. *AdAge*. 2001;72(26):S-10. Available online: https://adage.com/article/special-report-wtw99/viral-marketing-breaks/54417 (accessed on 1 October 2021).
79. Garfield B. Pepsi finally acknowledges real point of cola in new ads. *AdAge*. 2003;74(48):37. Available online: https://adage.com/article/news/garfield-s-adreview-pepsi-finally-acknowledges-real-point-cola-ads/96980 (accessed on 1 October 2021).
80. Geier JR, Philip H, Spillman M, Mathieu C, Grillo G. Letters. *AdAge*. 2004;75(1):12.
81. MacArthur K. Pepsi primes brand overhaul. *AdAge*. 2006;77(42):1-49. Available online: https://adage.com/article/print-edition/pepsi-primes-brand-overhaul/112499 (accessed on 1 October 2021).
82. Garfield B. Plato's logos may reject emotion, but he didn't work on Pepsi ads. *AdAge*. 2007;78(7):29.
83. Garfield B. Diet Pepsi spot is cute and quirky but muddles ultimate message. *AdAge*. 2007;78(21):53. Available online: https://adage.com/article/ad-review/diet-pepsi-spot-cute-quirky/116791 (accessed on 1 October 2021).
84. MacArthur K. Pepsi goes on $55 mil binge for Diet Max. *AdAge*. 2007;78(26):1–50. Available online: https://adage.com/article/news/pepsi-55-million-binge-diet-max/118744 (accessed on 1 October 2021).
85. Zmuda N. Pepsi Max drops the diet, aims to rekindle the cola wars. *AdAge*. 2010;81(30):1–20. Available online: https://adage.com/article/news/pepsi-max-drops-diet-aims-rekindle-cola-war/145505 (accessed on 1 October 2021).
86. Lafayette J. Research helps marketers make millennial connections. *Broadcasting & Cable*. 2013;143(10):15.
87. Zmuda N. Mtn Dew does national TV as it hunts for upside in key regions. *AdAge*. 2012;83(16):22. Available online: https://adage.com/article/cmo-strategy/mtn-dew-national-tv-hunts-upside-key-regions/234133 (accessed on 1 October 2021).

88. Zmuda N. Gatorade's new selling point: we're necessary performance gear. *AdAge*. 2012;83(1):2–21. Available online: https://adage.com/article/news/gatorade-s-performance-gear/231858 (accessed on 1 October 2021).
89. PepsiCo launches 'Pepsi Generations' campaign. *BevNet.* 11 May 2018. Available online: https://www.bevnet.com/news/2018/pepsico-launches-pepsi-generations-campaign/ (accessed on 1 October 2021).
90. Steinberg B. Pepsi's latest ad slogan promotes many drinks, not just one. *Variety.* 2 January 2020. Available online: https://variety.com/2020/biz/news/pepsi-advertising-slogan-thats-what-i-like-1203454944 (accessed on 1 October 2021).
91. Gatorade premieres 2020 international campaign with new ad featuring the greatest of all time: Leo Messi, Serena Williams, Usain Bolt and Michael Jordan. *PRNewswire*. 24 February 2020. Available online: https://markets.businessinsider.com/news/stocks/gatorade-unveils-goat-camp-where-athletes-go-to-become-greats-1028932325 (accessed on 1 October 2021).
92. Hughes LQ. Welch's picks another cute face for TV spots. *AdAge*. 2000;71(49):6. Available online: https://adage.com/article/news/welch-s-picks-cute-face-tv-spots/56068 (accessed on 1 October 2021).
93. Pollack J. Minute Maid ad budget will triple to boost brand. *AdAge*. 25 November 1996;67(48):29.
94. Pollack J, Petrecca L. V8, Tropicana pump spending for juices. *AdAge.* 1997;68(33):30. Available online: https://adage.com/article/news/v8-tropicana-pump-spending-juices-40-mil-allotted-fall-ads-marketers/71037 (accessed on 1 October 2021).
95. Schwirtz, M. Cramer-Krasselt. Best campaign spending between $1 million and $10 million. *MediaWeek.* 24 May 1999;9(21):12. https://worldradiohistory.com/Archive-Mediaweek/1999/Mediaweek-1999-05-24.pdf.
96. Kramer L. Ocean Spray sets $20 mil for Wellfleet Farms juices. *AdAge*. 1998;69(35):4. Available online: https://adage.com/article/news/ocean-spray-sets-20-mil-wellfleet-farms-juices/64671 (accessed on 1 October 2021).
97. Kramer L. Minute Maid puts OJ back in mornings. *AdAge.* 1999;70(10):3–61. Available online: https://adage.com/article/news/minute-maid-puts-oj-back-mornings-coca-cola-unit-shifts-trend-touting-drink-times-day/62536 (accessed on 1 October 2021).
98. Breaking: Simply Orange. *AdAge*. 2001;72(23):44. Available online: https://adage.com/article/news/breaking-simply-orange/54601 (accessed on 1 October 2021).
99. Lee J, Gao Z, Brown MG. A study on the impact of package changes on orange juice demand. *J of Retailing Consumer Services.* 2010;17(6):487–491. https://dx.doi.org/10.1016/j.jretconser.2010.08.003.
100. Zmuda N. OJ ditches beauty shots, goes for 'double rainbow'. *AdAge*. 2011;82(5):2–8. Available online: https://adage.com/article/news/minute-maid-taps-quirky-campaign-mom-buyers/148560 (accessed on 1 October 2021).
101. Zmuda N. Tropicana goes back to nature in new global pitch. *AdAge.* 2012;83(8):3–19. Available online: https://adage.com/article/global-news/tropicana-back-nature-global-pitch/232819 (accessed on 1 October 2021).
102. Kretchmer SB. Advertainment: the evolution of product placement as a mass media marketing strategy. *J Promot Manag*. 2004;10(1/2):37–54. https://doi.org/10.1300/J057v10n01_04.
103. Kramer L, Cuneo A. Nestle touting its instant coffees. *AdAge*. 1997;68(44):4. Available online: https://adage.com/article/news/nestle-touting-instant-coffees/70233 (accessed on 1 October 2021).

104. Thompson S. Favre signs as pitchman for Real Pure drink lines. *AdAge.* 2000;71(18):18. Available online: https://adage.com/article/news/favre-signs-pitchman-real-pure-drink-lines/58621 (accessed on 1 October 2021).
105. Latest News. *AdAge.* 2004;75(13):1-2.
106. Non-alcoholic beverages. *AdAge.* 2002;73(18):S-10.
107. Evian launches new creative ad campaign. Bottled water brand invites consumers to hashtag #Liveyoung. *Beverage Industry.* 10 July 2018. Available online: https://www.bevindustry.com/articles/91271-evian-launches-new-creative-ad-campaign (accessed on 3 May 2021).
108. Chura H. Soy milk goes mainstream with ads. *AdAge*. 2000;71(20):52. Available online: https://adage.com/article/news/soy-milk-mainstream-ads/58479 (accessed on 1 October 2021).
109. Cooper M. Silk shows consumers how almond milk is grown, not made. Campaign Live U.S. Haymarket Media Group Ltd. 14 April 2021. Available online: https://www.campaignlive.com/article/silk-shows-consumers-almond-milk-grown-not-made/1712905 (accessed on 1 October 2021).
110. Hiebert P. With sales up, silk looks to turn more shoppers to plant-based milk. *AdWeek.* 14 April 2021. Available online: https://www.adweek.com/performance-marketing/with-sales-up-silk-looks-to-turn-more-shoppers-to-plant-based-milk/ (accessed on 3 May 2021).
111. Wohl J. 'It's like milk, but made for humans,' CEO Toni Petersson sings while standing in an oat field. *AdAge.* 7 February 2021. Available online: https://adage.com/article/special-report-super-bowl/see-oatly-bring-2014-ad-banned-sweden-us-its-super-bowl-debut/2312321 (accessed on 1 October 2021).
112. Cohen DA, Bogart L, Castro G, Rossi AD, Williamson S, Han B. Beverage marketing in retail outlets and The Balance Calories Initiative. *Prev Med.* 2018;115:1–7. https://doi.org/10.1016/j.ypmed.2018.07.014.
113. Cohen DA, Bogart LM, Han B, Williamson S, Castro G. High consumption of energy-dense nutrient-poor foods among low-income groups in the Mississippi Delta and Alabama. *Public Health Nutr.* 2020;23(6):1067–1075. https://doi.org/10.1017/S1368980019002817.
114. The Coca-Cola Company. Coca-Cola The Great Meal #togethertastesbetter. 30 June 2020. Available online: https://www.youtube.com/watch?v=vUMQeNw2QDA&feature=youtu.be (accessed 1 October 2021).
115. The Coca-Cola Company. Together We Must. 2020. Available online: https://us.coca-cola.com/together/ (accessed on 1 October 2021).
116. The Coca-Cola Company. Refreshing the World and Making a Difference. 2021. Available online: https://www.coca-colacompany.com/home (accessed on 1 October 2021).
117. Brei V, Böhm S. Corporate social responsibility as cultural meaning management: a critique of the marketing of 'ethical' bottled water. *Business Ethics, Environ & Responsibility.*2011;20(3):233–252. https://doi.org/10.1111/j.1467-8608.2011.01626.x.
118. Steinberg B, Zmuda N. Pepsi may play second string in Super Bowl. *AdAge.* 2009;80(42):3. Available online: https://adage.com/article/special-report-super-bowl/pepsi-play-string-super-bowl/141063 (accessed on 1 October 2021).
119. Zmuda N, Patel K. Pass or fail, Pepsi's Refresh will be case for marketing textbooks. *AdAge.* 2010;81(6):1-18. Available online: https://adage.com/article/digital/marketing-pepsi-refresh-case-marketing-textbooks/141973 (accessed on 1 October 2021).

120. Iezzi T. Post-digital era brings traits of web to real world. *AdAge.* 2010;81(21):1–29. Available online: https://adage.com/article/digital/post-digital-era-brings-traits-web-real-world/144042 (accessed on 1 October 2021).
121. Zmuda N. Who are the big Pepsi Refresh winners? Local bottlers and community groups. *AdAge.* 1 November 2010. Available online: https://adage.com/article/news/cola-bottlers-community-groups-pepsi-refresh-s-big-winners/146814 (accessed on 1 October 2021).
122. Crain R. Why I think Pepsi Refresh needed the Super Bowl. *AdAge.* 2010;81(39):2-22. Available online: https://adage.com/article/rance-crain/i-pepsi-refresh-needed-super-bowl/146815 (accessed on 1 October 2021).
123. Applebaum M. RPM Group and Pepsi deliver. *MediaWeek.* 2010;20(39):A8.
124. Victor D. Pepsi pulls ad accused of trivializing Black Lives Matter. *The New York Times*. 5 April 2017. https://www.nytimes.com/2017/04/05/business/kendall-jenner-pepsi-ad.html.
125. Black art rising. n.d. LIFEWTR. Available online: https://www.theblackartrising.com (accessed on 1 October 2021).
126. PepsiCo. Food for Good; PepsiCo.: Harrison, NY, USA, 2021; Available online: https://www.pepsicofoodforgood.com/ (accessed on 1 October 2021).
127. Yodice, A. LIFEWTR® Partners with Issa Rae to Launch "Life Unseen™," a New Platform for Fair Representation in the Arts. *PRN Newswire.* 14 April 2021. Available online: https://www.prnewswire.com/news-releases/lifewtr-partners-with-issa-rae-to-launch-life-unseen-a-new-platform-for-fair-representation-in-the-arts-301268725.html (accessed on 1 October 2021).
128. Nestle Waters pledge. Nestle. 2021. Available online: https://www.nestle.com/csv/impact/water/nestle-waters-pledge (accessed on 1 October 2021).
129. NewsDesk. Nestlé Pure Life concentrates on hydration in global ad campaign. *FoodBevMedia.* 31 July 2017. Available online: https://www.foodbev.com/news/nestle-pure-life-concentrates-on-hydration-in-global-ad-campaign/ (accessed on 1 October 2021).
130. Werner RO, Cohen D. Regulation of unfair competition. *J Mark*. 1980;44(2):99–100. https://doi.org/10.2307/1249981.
131. Keegan CM. Revisitation. *MedieKultur: J Media & Commun Res*. 2016;32(61):26-41. ISSN 1901-9726.
132. Wechsler H, Wernick SM. A social marketing campaign to promote low-fat milk consumption in an inner-city Latino community. *Public Health Rep.* 1992; 107: 202–207. https://www.ncbi.nlm.nih.gov/pmc/articles/PMC1403632/.
133. Reger B, Wootan MG, Booth-Butterfield S, Smith H. 1% or less: a community-based nutrition campaign. *Public Health Rep.* 1998;113(5):410–419. https://pubmed.ncbi.nlm.nih.gov/9769765.
134. Reger B, Wootan MG, Booth-Butterfield S. Using mass media to promote healthy eating: a community-based demonstration project. *Prev Med.* 1999;29(5):414–421. https://doi.org/10.1006/pmed.1998.0570.
135. Reger B, Wootan MG, Booth-Butterfield S. A comparison of different approaches to promote community-wide dietary change. *Am J Prev Med*. 2000;18(4):271–275. https://doi.org/10.1016/S0749-3797(00)00118-5.
136. Booth-Butterfield S, Reger B. The message changes belief and the rest is theory: the "1% or less" milk campaign and reasoned action. *Prev Med.* 2004;39:581–588. https://doi.org/10.1016/j.ypmed.2004.02.013.
137. Wootan MG, Reger-Nash B, Booth-Butterfield S, Cooper L. The cost-effectiveness of 1% or less media campaigns promoting low-fat milk consumption. *Prev Chronic Dis.* 2005;2:A05. http://www.ncbi.nlm.nih.gov/pmc/articles/PMC1435702/.

138. Maddock J, Maglione C, Barnett JD, Cabot C, Jackson S, Reger-Nash B. Statewide implementation of the 1% or Less Campaign. *Health Educ Behav.* 2007;34(6):953–963. http://dx.doi.org/10.1177/1090198106290621.
139. Hinkle AJ, Mistry R, McCarthy WJ, Yancey AK. Adapting a 1% or less milk campaign for a Hispanic/Latino population: the Adelante Con Leche Semi-descremada 1% experience. *Am J Health Promot.* 2008;23(2):108–111. http://dx.doi.org/10.4278/ajhp.07080780.
140. Cuneo AZ. Jeff Manning Got Milk? *AdAge.* 26 June 1995;66(26):54. Available online: https://adage.com/article/news/marketing-100-jeff-manning-milk/80716 (accessed on 1 October 2021).
141. Gleason M, Kelly KJ. Men are newest target for 'milk mustache' ads. *AdAge*. 1996;67(27):10. Available online: https://adage.com/article/news/men-newest-target-milk-mustache-ads-travolta-schwarzenegger/78519 (accessed on 1 October 2021).
142. Pollack J. Who's got milk? In marketing, two execs do. *AdAge*. 1996;67(41):s4. Available online: https://adage.com/article/news/power-50-milk-marketing-execs-beverages-growing-ad-budgets-bring-associations-spotlight/76841 (accessed on 1 October 2021).
143. Pollack J, Cuneo AZ, Petrecca L, Kerwin AM. 'Milk mustache' dollars shifting from mags to TV. *AdAge*. 1998;69(23):1–50. Available online: https://adage.com/article/news/milk-mustache-dollars-shifting-mags-tv/31130 (accessed on 1 October 2021).
144. Pollack J, Cuneo AZ. Milk groups add nutrition pitch in plan for 2000. *AdAge.* 1999;70(31):3–43. Available online: https://adage.com/article/news/milk-groups-add-nutrition-pitch-plan-2000-bozell-tv-work-expands-earlier-ads-fun-situations/61655 (accessed on 1 October 2021).
145. Thompson S. Suiza Foods extends test of 'Milk made better' ads. *AdAge.* 2000;71(2):4. Available online: https://adage.com/article/news/suiza-foods-extends-test-milk-made-ads/59816 (accessed on 1 October 2021).
146. Thompson S. Milk producers back Britney Spears tour. *AdAge.* 2000;71(10):68. Available online: https://adage.com/article/news/milk-producers-back-britney-spears-tour/59152 (accessed on 1 October 2021).
147. Fitzgerald K. Milk tailors effort to teens. *AdAge.* 18 February 2002;73(7):16.
148. Pszczola DE. Ingredients provide new levels of innovation in dairy products. *Food Tech.* 2003;57(8).
149. Manning J, Keller KL. Got advertising that works? How the "Got Milk?" campaign shook customers out of their milk malaise. *Mark Manag*. 2004;13(1):16–20. https://studylib.net/doc/8870338/got-advertising-that-works%3F.
150. Van der Waldt DLR, Schleritzko NE, Van Zyl K. Paid versus unpaid celebrity endorsement in advertising: an exploration. *African J Business Manag*. 2007;1(7):185-191. https://repository.up.ac.za/handle/2263/5163
151. Toma leche? Grupo Gallegos, Long Beach, Calif. *AdAge*. 5 February 2007;78(6):28.
152. Miles L. Best use of out of home. *MediaWeek*. 18 June 2007;17(25):SR28.
153. Marketing news. *AdAge*. 12 October 2015;86(17):0004.
154. Zhou M, Rajamohan S, Hedrick V, et al. Mapping the celebrity endorsement of branded food and beverage products and marketing campaigns in the United States, 1990-2017. *Int J Environ Res Public Health*. 2019;16(19). https://doi.org/10.3390/ijerph16193743.
155. Zhou M, Rincón-Gallardo Patiño S, Hedrick VE, Kraak VI. An accountability evaluation for the responsible use of celebrity endorsement by the food and beverage industry to promote healthy food environments for young Americans: a narrative reiew to inform obesity prevention policy. *Obes Rev*. 2020;21(12):e13094. https://doi.org/10.1111/obr.13094.

156. Zhou M, Kraak VI. A mixed-methods study of American Millennials' views about celebrity endorsement of foods and beverages. *Health Promot Int*. 2021. https://doi.org/10.1093/heapro/daab048.
157. Holt DB. Got Milk. *Advertising Educational Foundation*. 2002. Available online: https://aef.com/classroom-resources/case-histories/got-milk/ (accessed on 1 October 2021).
158. Manning J. Got Milk? Marketing by Association. *Associations Now*. The Center for Association Leadership, July 2016. Available online: https://www.asaecenter.org/resources/articles/an_plus/2016/january/got-milk-marketing-by-association (accessed on 1 October 2021).
159. Wheaton K. Milk's protein play could score. *AdAge*. 10 March 2014;85(5):30. Available online: https://adage.com/article/ad-review/milk-working-milk-anymore/292001 (accessed on 1 October 2021).
160. Super Bowl QBs don milk mustaches. *AdAge*. 20 January 1997;68(3):2. Available online: https://adage.com/article/news/super-bowl-qbs-don-milk-mustaches/20065 (accessed on 1 October 2021).
161. Pollack J. Milk. *AdAge*. 30 June 1997;68(26):s1.
162. Pollack J, Kerwin AM. MilkPEP crafts cross-promotions with magazine partners for 1998. *AdAge*. 1997;68(30):3-33. Available online: https://adage.com/article/news/milkpep-crafts-cross-promotions-magazine-partners-1998-homes-life-tie-plans/71226 (accessed on 1 October 2021).
163. Pollack J. Milk marches into new era with branded product push. *AdAge.* 1998;69(4):14. Available online: https://adage.com/article/news/milk-marches-era-branded-product-push/31002 (accessed on 1 October 2021).
164. Fitzgerald K. Tables turned: promos solicit creative spark. *AdAge.* 1998;69(41):50. Available online: https://adage.com/article/news/tables-turned-promos-solicit-creative-spark-goal-tactic-immerse-public-brand/64123 (accessed on 1 October 2021).
165. Pollack J, Teinowitz I. 'Milk mustache' ads, Bozell come under fire. *AdAge*. 1998;69(44):2.
166. Schulberg J. *The Milk Mustache Book: A Behind-the-Scenes Look at America's Favorite Advertising Campaign.* New York, NY: Ballantine Books, 1998.
167. Hsu CK, McDonald D. An examination on multiple celebrity endorsers in advertising. *J Product & Brand Manag*. 2002;11(1):19. doi: 10.1108/10610420210419522.
168. Stanley TL. Goodby, Silverstein & Partners. *Mediaweek.* 15 June 2009;19(24):6.
169. John R, Finnell KJ, Scott-Kaliki MS, DeBerry S.M. A case study of two successful social marketing interventions to promote 1% low-fat milk consumption. *Soc Market Quarter.* 2019;25(2):137–159. https://doi.org/10.1177/1524500418824292.
170. Robert J, Finnell KJ, Kerby DS, Owen J, Hansen K. Reactions to a low-fat milk social media intervention in the US: the Choose 1% Milk campaign. *Beverages.* 2017;3(4):47. https://doi.org/10.3390/beverages3040047
171. America's Milk Companies. Got milk? Team milk. Meet the athletes. 2021. Available online: https://gonnaneedmilk.com/athletes/ (accessed on 1 October 2021).
172. Partnership for a Healthier America. About Drink Up. 2021. https://www.ahealthieramerica.org/articles/about-drink-up-260 (accessed on 1 October 2021).

173. Farley T, Halper HS, Carlin AM, Emmerson KM, Foster KN, Fertig AR. Mass media campaign to reduce consumption of sugar-sweetened beverages in a rural area of the United States. *Am J Public Health*. 2017;107(6):989–995. https://doi.org/10.2105/AJPH.2017.303750.
174. Falbe J, Madsen K. Growing momentum for sugar-sweetened beverage campaigns and policies: costs and considerations. *Am J Public Health*. 2017;107(6):835–838. doi: 10.2105/AJPH.2017.303805
175. Peterson M. Social marketing food and beverage choices: it's how you say it. *Delaware Academy of Medicine*. 2016. doi: 10.32481/djph.2016.06.007.
176. Sisti JS, Mezzacca TA, Anekwe A, Farley SM. Examining trends in beverage sales in New York City during comprehensive efforts to reduce sugary drink consumption, 2010-2015. *J Comm Health*. 2021, 46, 609–617. https://doi.org/10.1007/s10900-020-00911-y.
177. Bonnevie E, Morales O, Rosenberg SD, Goldbarg J, Silver M, Wartella E, Smyser J. Evaluation of a campaign to reduce consumption of sugar-sweetened beverages in New Jersey. *Prev Med.* 2020;136:106062. https://doi.org/10.1016/j.ypmed.2020.106062.
178. Juice Products Association. Sip Smarter. *Juice Nutrition News*. 2020. Available online: https://sipsmarter.org/research/juice-nutrition/ (accessed on 1 October 2021).
179. Healthy Berkeley Partnerships. *Healthy Berkeley.* 2021. Available online: http://www.healthyberkeley.com/ (accessed on 1 October 2021).
180. City of Albany California. Sugar Sweetened Beverage Tax. 2020. Available online: https://www.albanyca.org/departments/finance/sugar-sweetened-beverage-tax (accessed on 1 October 2021).
181. Be Ready. Be Hydrated. Drink Water Campaign. n.d. Available online: https://bereadybehydrated.com/ (accessed on 1 October 2021).
182. Freudenberg N, Bradley SP, Serrano M. Public health campaigns to change industry practices that damage health: an analysis of 12 case studies. *Health Educ Behav.* 2009;36(2):230–249. https://doi.org/10.1177%2F1090198107301330.
183. Centers for Disease Control and Prevention. Rethink Your Drink/Choose Healthy Drinks. 2020. Available online: https://cdc.thehcn.net/promisepractice/index/view?pid=30147 (accessed on 1 October 2021).
184. Bleakley A, Jordan AB, Hennessy M, Glanz K, Strasser A, Vaala S. Do emotional appeals in public service advertisements influence adolescents' intention to reduce consumption of sugar-sweetened beverages? *J Health Commun*. 2015;20(8):938–948. https://doi.org/10.1080/10810730.2015.1018593.
185. Dillard JP, Kim J, Li SS. Anti-sugar-sweetened beverage messages elicit reactance: effects on attitudes and policy preferences. *J Health Commun*. 2018;23(8):703–711. https://doi.org/10.1080/10810730.2018.1511012.
186. James SA, White AH, Paulson SW, Beebe LA. Factors associated with sugar-sweetened beverage consumption in adults with children in the home after a statewide health communications program. *BMC Nutr*. 2020;6:23. https://doi.org/10.1186/s40795-020-00349-4.
187. Kansagra SM, Kennelly MO, Nonas CA, Curtis CJ, Van Wye G, Goodman A, Farley TA. Reducing sugary drink consumption: New York City's approach. *Am J Public Health*. 2015;105(4):361-e64. https://ajph.aphapublications.org/doi/full/10.2105/AJPH.2014.302497

188. Popkin BM, Hawkes C. Sweetening of the global diet, particularly beverages: patterns, trends, and policy responses. *Lancet Diabetes Endocrinol.* 2016;4(2):174–186. http://dx.doi.org/10.1016/S2213-8587(15)00419-2.
189. Garfield B. NYC's bottle-of-fat ads as nauseating as industry response. *AdAge.* 2009;80(29):26. Available online: https://adage.com/article/ad-review/nyc-s-soft-drink-psa-nauseating-cola-industry-response/138850 (accessed on 1 October 2021).
190. Solana K. Drinks Destroy Teeth reaches milestone. Indiana program tackles sugary, acidic drinks' effect on children's teeth. October 30, 2019. *American Dental Association (ADA) News*. Available online: https://www.ada.org/en/publications/ada-news/2019-archive/october/drinks-destroy-teeth-reaches-milestone (accessed on 1 October 2021).
191. Cook County Government. Cook county officials launch "Rethink Your Drink" media campaign. 9 January 2012. Available online: https://www.cookcountyil.gov/news/cook-county-officials-launch-"rethink-your-drink"-media-campaign (accessed on 1 October 2021).
192. Samuels & Associates. Evaluation of San Francisco's Social Marketing Campaign "Pouring on the Pounds." California Obesity Prevention Program. 2010. Available online: https://www.iccp-portal.org/sites/default/files/multimediaresources/San%20Francisco_Pouring_on_the_Pounds_Report.pdf (accessed on 1 October 2021).
193. Te V, Ford P, Schubert L. Exploring social media campaigns against sugar-sweetened beverage consumption: a systematic search. *Cogent Med*. 2019;6(1):1607432. http://dx.doi.org/10.1080/2331205X.2019.1607432.
194. Boston Public Health Commission. FatSmack. Campaign Information. 2011. Available online: https://www.bphc.org/whatwedo/healthy-eating-active-living/fatsmack/aboutthecampaign/Pages/aboutthecampaign.aspx (accessed on 3 May 2021).
195. Center for Science in the Public Interest. Cities, CSPI, & Health Groups Announce Major New Campaign to Reduce Soda Consumption. "Life's Sweeter with Fewer Sugary Drinks," Say Health Officials. August 31, 2011 [media release]. Available online: https://cspinet.org/new/201108311.html (accessed on 1 October 2021).
196. Barragan NC, Noller AJ, Robles B et al. The "sugar pack" health marketing campaign in Los Angeles County, 2011-2012. *Health Promot Pract*. 2014;15(2):208–216. https://doi.org/10.1177/1524839913507280.
197. Robles B, Blitstein JL, Lieberman AJ, Barragan NC, Gase LN, Kuo T. The relationship between amount of soda consumed and intention to reduce soda consumption among adults exposed to the Choose Health LA 'Sugar Pack' health marketing campaign. *Public Health Nutr*. 2015;18(14):2582–2591. https://doi.org/10.1017/S1368980014003097.
198. Gase LN, Robles B, Barragan NC, Kuo T. Relationship between nutritional knowledge and the amount of sugary-sweetened beverages consumed in Los Angeles County. *Health Educ Behav*. August 2014;41(4):431-439. https://doi.org/10.1177/1090198114529128.
199. First Five Contra Costa Children and Families Commission. About Sugar Bites. n.d. Available online: http://www.cutsugarydrinks.org/en/about (accessed on 1 October 2021).
200. Boles M, Adams A, Gredler A, Manhas S. Ability of a mass media campaign to influence knowledge, attitudes, and behaviors about sugary drinks and obesity. *Prev Med*. 2014;67(Suppl 1):S40–S45. https://doi.org/10.1016/j.ypmed.2014.07.023.

201. Bleakley A, Jordan, A, Mallya G, Hennessy M, Piotrowski JT. Do you know what your kids are drinking? Evaluation of a media campaign to reduce consumption of sugar-sweetened beverages. *Am J Health Promot*. 2018;32(6):1409–1416. https://doi.org/10.1177/0890117117721320.
202. Schwartz MB, Schneider GE, Choi YY et al. Association of a community campaign for better beverage choices with beverage purchases from supermarkets. *JAMA Intern Med*. 2017;177(5):666–674. https://doi.org/10.1001/jamainternmed.2016.9650.
203. Aguilar-Santos R. "Sugar Smarts" bilingual campaign in Boston against sugary drinks for kids. Salud America! 30 January 2015. Available online: https://salud-america.org/sugar-smarts-bilingual-campaign-in-boston-against-sugary-drinks-for-kids/ (accessed on 1 October 2021).
204. Hartigan P, Patton-Ku D, Fidler, C, Boutelle KN. Rethink Your Drink. *Health Promot Pract*. 2017;18(2):238–244. https://doi.org/10.1177/1524839915625215.
205. Chavez M. "Cavities Get Around" title night with the Colorado Rapids. 28 August 2014. Blog. Available online: https://blog.deltadentalco.com/2014/08/cavities-get-around-title-night-with-the-colorado-rapids/ (accessed on 1 October 2021).
206. Hornsby WC, Bailey W, Braun PA, Weiss K, Heichelbech J. Busting the baby teeth myth and increasing children's consumption of tap water: building public will for children's oral health in Colorado. *Front Public Health.* 2017;5:238. https://doi.org/10.3389/fpubh.2017.00238.
207. Minneapolis Health Department. Re-think Your Drink, Every Sip Counts! 2020. Available online: https://rethinkyourdrink.minneapolismn.gov/ (accessed on 1 October 2021).
208. California Department of Health. Rethink Your Drink. California Department of Public Health (CDPH) Nutrition Education and Obesity Prevention Branch (NEOPB). 21 October 2020. Available online: https://www.cdph.ca.gov/Programs/CCDPHP/DCDIC/NEOPB/Pages/RethinkYourDrink.aspx (accessed on 1 October 2021).
209. Maghrabi P, Terry M. Effectiveness of a community-based health promotion "Rethink Your Drink" on reducing sugary beverage consumption: a case study. *J Exercise Nutr*. 2021;1(5):1–4. https://www.journalofexerciseandnutrition.com/index.php/JEN/article/view/24.
210. Caldwell JI, Robles B, Tyree R, Fraser RW, Dumke KA, Kuo T. Does exposure to the Choose Water campaign increase parental intentions to promote more water and less sugar-sweetened beverage consumption? *Am J Health Promot*. 2020;34(5):555–558. https://doi.org/10.1177%2F0890117120908785.
211. New York City Department of Health. Health Department Launches "The Sour Side of Sweet" Media Campaign on the Health Hazards of Sugary Drinks. 31 July 2017 [press release]. Available online: https://www1.nyc.gov/site/doh/about/press/pr2017/pr065-17.page#:~:text=The%20new%20campaign%2C%20%E2%80%9CThe%20Sour,to%20give%20them%20to%20children.&text=In%202015%2C%20almost%20a%20quarter,or%20more%20sugary%20drinks%20daily (accessed on 1 October 2021).
212. Healthy Beverage Partnership. Hidden Sugar. Denver Public Health, Colorado. 2017. Available online: http://www.hidden-sugar.org/ (accessed on 1 October 2021).

213. Healthy Active Arkansas. Rethink your drink educational campaign toolkit. Revised September 2017. Available online: https://healthyactive.org/wp-content/uploads/2017/11/Rethink-Your-Drink-Toolkit.pdf (accessed on 1 October 2021).
214. American Heart Association. Healthy for Good. Sip Smarter. 2018. Available online: https://www.heart.org/en/healthy-living/healthy-eating/eat-smart/sugar/sip-smarter-infographic (accessed on 1 October 2021).
215. Robert Wood Johnson Foundation Healthy Eating Research Program. Healthy Drinks, Healthy Kids. n.d. Available online: https://healthydrinkshealthykids.org (accessed on 1 October 2021).
216. Global Dump Soft Drinks Campaign. Letter to Mr. E. Neville Isdell, Chief Executive Officer, Coca Cola Company. January 3, 2008. Center for Science in the Public Interest. Available online: https://cspinet.org/sites/default/files/attachment/cokeletter.pdf (accessed on 1 October 2021).
217. Global Dump Soft Drinks Campaign. Letter to Ms. Indra K. Nooyi, Chairman of the Board and Chief Executive Officer, PepsiCo Inc. January 3, 2008. Center for Science in the Public Interest. Available online: https://cspinet.org/sites/default/files/attachment/pepsiletter.pdf (accessed on 1 October 2021).
218. Center for Science in the Public Interest. Consumer groups in 20 countries urge Coke, Pepsi to limit soft drink marketing to children. 3 January 2008. Available online: https://cspinet.org/new/200801031.html (accessed on 1 October 2021).
219. Dunk the Junk. n.d. Available online: http://www.dunkthejunk.org/ (accessed on 1 October 2021).
220. Center for Science in the Public Interest. Introducing "The Real Bears" animated short film takes on Big Soda; features original song by Jason Mraz. October 10, 2012. [news release]. Available online: https://cspinet.org/new/201210101.html (accessed on 1 October 2021).
221. Allen Meyer Design. Soda Sucks outsmarts the advertisers. n.d. Available online: https://allenmeyerdesign.com/project/soda-sucks/ (accessed on 1 October 2021).
222. Marriott RW, Dillard JP. Sweet talk for voters: a survey of persuasive messaging in ten U.S. sugar-sweetened beverage tax referendums. *Crit Public Health*. 2020. https://doi.org/10.1080/09581596.2020.1724263.
223. Center for Science in the Public Interest. Sugary drinks. n.d. Available online: https://cspinet.org/eating-healthy/foods-avoid/sugary-drinks (accessed on 1 October 2021).
224. University of California San Francisco. Behind the Bigger Picture Campaign. n.d. Available online: https://charm.ucsf.edu/impact/behind-bigger-picture-campaign (accessed on 1 October 2021).
225. Open truth: sugary drinks are making us sick. n.d. Available online: http://www.opentruthnow.org (accessed on 1 October 2021).
226. Yazzie D, Tallis K, Curley C et al. The Navajo Nation Healthy Diné Nation Act: a two percent tax on foods of minimal-to-no nutritious value, 2015–2019. *Prev Chronic Dis*. 2020;17:200038. http://dx.doi.org/10.5888/pcd17.200038.
227. Somji A, Nixon L, Mejia P, Aziz A, Arbatman L, Dorfman L. Soda tax debates in Berkeley and San Francisco: an analysis of social media, campaign materials and news coverage. *Berkeley Media Studies Group.* January 2016. http://www.bmsg.org/wp-content/uploads/2016/01/bmsg_soda_tax_debates2016_web.pdf.

228. Altman EA, Madsen KA, Schmidt LA. Missed opportunities: the need to promote public knowledge and awareness of sugar-sweetened beverage taxes. *Int J Environ Res Public Health.* 2021;18(9):4607. https://doi.org/10.3390/ijerph18094607
229. Falbe J, Thompson HR, Becker CM, Rojas N, McCulloch CE, Madsen KA. Impact of the Berkeley excise tax on sugar-sweetened beverage consumption. *Am J Public Health*. 2016;106:1865–1871. https://doi.org/10.2105/AJPH.2016.303362.
230. Healthy Food America. Policy Profile: Berkeley, CA Sweetened Drink Tax. May 2018. Available online: http://www.healthyfoodamerica.org/policy_profile_berkeley_ca_sugary_drink_tax (accessed on 1 October 2021).
231. Falbe J, Lee MM, Kaplan S, Rojas NA, Ortega Hinojosa AM, Madsen KA. Higher sugar-sweetened beverage retail prices after excise taxes in Oakland and San Francisco. *Am J Public Health.* 2020;110:1017–1023. https://doi.org/10.2105/AJPH.2020.305602.
232. Healthy Food America. Policy Profile: Oakland, CA Sweetened Drink Tax. May 2018. Available online: http://www.healthyfoodamerica.org/policy_profile_oakland_ca_sweetened_drink_tax (accessed on 1 October 2021).
233. Asada Y, Taher S, Pipito A, Chriqui JF. Media coverage and framing of Oakland's sugar-sweetened beverage tax, 2016-2019. *Am J Health Promot.* 2021;35:698–702. https://doi.org/10.1177/0890117120986104.
234. Asada Y, Pipito AA, Chriqui JF, Taher S, Powell LM. Oakland's sugar-sweetened beverage tax: honoring the "spirit" of the ordinance toward equitable implementation. *Health Equity.* 2021;5(1):35-41. https://doi.org/10.1089/heq.2020.0079.
235. Healthy Food America. Policy Profile: San Francisco. Sweetened Drink Tax. May 2018. Available online: https://www.healthyfoodamerica.org/policy_profile_san_francisco_ca_sugary_drink_tax (accessed on 1 October 2021).
236. Healthy Food America. Policy Profile: Albany, CA Sweetened Drink Tax. May 2018. Available online: http://www.healthyfoodamerica.org/policy_profile_albany_ca_sugary_drink_tax (accessed on 1 October 2021).
237. Healthy Food America. Policy Profile: Philadelphia, PA Sweetened Drink Tax. May 2018. Available online: http://www.healthyfoodamerica.org/policy_profile_philadelphia_pa_sweetened_drink_tax (accessed on 1 October 2021).
238. Lawman HG, Bleich SN, Yan J. One-year changes in sugar-sweetened beverage consumers' purchases following implementation of a beverage tax: a longitudinal quasi-experiment. *Am J Clin Nutr.* 2020;112*:*644–651. https://doi.org/10.1093/ajcn/nqaa158.
239. Bleich SN, Lawman HG, LeVasseur MT, et al. The association of a sweetened beverage tax with changes in beverage prices and purchases at independent stores. *Health Aff (Millwood)*. 2020;39:1130–1139. https://doi.org/10.1377/hlthaff.2019.01058.
240. Healthy Food America. Policy Profile: Boulder, Colorado Sweetened Drink Tax. May 2018. Available online: https://www.healthyfoodamerica.org/policy_profile_boulder_co_sugary_drink_tax (accessed on 1 October 2021).
241. Healthy Food America. Policy Profile: Seattle, Washington Sweetened Drink Tax. May 2018. Available online: http://www.healthyfoodamerica.org/policy_profile_seattle_wa_sugary_drink_tax (accessed on 1 October 2021).
242. Krieger J, Magee K, Hennings T, Schoof J, Madsen KA. How sugar-sweetened beverage tax revenues are being used in the United States. *Prev Med Rep.* 2021;23:101388. https://doi.org/10.1016/j.pmedr.2021.101388.
243. Falbe J, Adler SS, Roberto CA. Sugar-sweetened beverage tax preemption and the urgency of unified mobilization. *Am J Public Health.* 2021;111(4):546–548. https://doi.org/10.2105/AJPH.2021.306163.

244. Crosbie E, Pomeranz JL, Wright KE, Hoeper S, Schmidt L. State preemption: an emerging threat to local sugar-sweetened beverage taxation. *Am J Public Health*. 2021;111(4):677–686. https://doi.org/10.2105/AJPH.2020.306062.

Supplemental Table 2

Comprehensive evidence summary of 24 evaluations for 20 unique U.S. beverage media campaigns organized by the typology category, goal, target population and outcomes, 1992-2021

Supplemental Table 2 provides detailed evidence for 24 published evaluations for 20 unique U.S. media campaigns oganized into four typology categories and describe the goal, objectives and target audiences; short-term outcomes (i.e., influence on cognitive outcomes including awareness, knowledge, attitudes and beliefs); mid-term outcomes (i.e., influence on retail policies, environments or individual behaviors); and long-term outcomes (i.e., influence on societal norms, values and population behaviors to educe obesity and diet-related non-communicable dieases including type 2 diabetes and cardiovascular diseases).

**Typology Category**	***Campaign name***<br>Location (city, state)<br>time frame<br><br>**Goal**	**Target population**<br><br>**Strategies**<br><br>**Theory or conceptual framework used to plan the campaign**	**Short-term outcomes**<br><br>**Cognitive outcomes**<br>(i.e., awareness, attitudes, beliefs, knowledge or preferences)	**Mid-term outcomes**<br><br>**Behavioral outcomes**<br>(i.e., reported or measured individual behaviors)<br><br>**Retail outcomes**<br>(revenue or sales)	**Long-term outcomes**<br><br>**Social norm, policy and population health outcomes**<br>(i.e., institutional policy change, weight, obesity or diabetes)
**1. Corporate advertising, marketing or entertainment branded campaigns** (n = 0 evaluations were identified for sugary beverage brands or products)					
**2. Corporate social responsibility, public relations, cause marketing campaigns** (*n* = 2 evaluations were identified for the Balance Calories Initiative campaign)					
Bogart et al. 2019[51]	***Balance Calories Initiative***<br>Montgomery, AL; North Mississippi Delta, MS; and Eastern Los Angeles, CA (2016-2017)	**Population:** Low-income communities (*n* = 8-10) with low access to reduced-calorie or no-calorie beverages.	**Cognitive outcomes**<br>Parents (*n* = 12) and youth (*n* = 24) saw the BCI messages. Many parents and youth misunderstood the BCI messages,	**Behavioral or retail outcomes**<br>Not reported	**Social norm, policy and population health outcomes**<br>Not reported

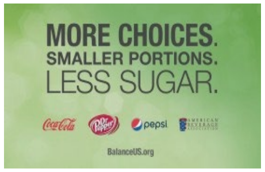	**Goal:** Decrease per capita intake of energy from beverages by 20% by 2025.	**Strategies:** The American Beverage Association's (ABA's) BCI introduced and expanded reduced-calorie products and smaller-portion packages; changed product placement (e.g., end-aisle and checkout displays featuring only reduced-calorie beverages, repositioning reduced calorie beverages on shelves); provided coupons and promotions for reduced- and no-calorie options; conducted taste tests; and promoted energy balance messages on beverage coolers and billboards.<br><br>**Theory or framework:**<br>Not reported	interpreted that they should drink *more* sugary beverages or that they needed to equalize healthy and unhealthy beverage intake.<br><br>Store managers (n = 4) were aware of BCI and (n = 1) had communicated with firms about BCI.		
Cohen et al. 2018[52]<br>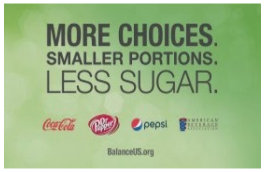	***Balance Calories Initiative***<br>Montgomery, AL; North Mississippi Delta, MS; and Eastern Los Angeles, CA (2016-2017)<br><br>**Goal:** Decrease per capita intake of energy from beverages by 20% by 2025.	**Population:** Low-income communities (n = 8-10) with low access to reduced-calorie or no-calorie beverages.<br><br>**Strategies:** Product placement in low-income communities featured merchandising, couponing and other incentives; and more intensive efforts to reduce SSB consumption in the selected communities.<br><br>**Theory or framework:**<br>Not reported	**Cognitive outcomes**<br>Not reported	**Behavioral outcomes**<br>Not reported<br><br>**Retail outcomes**<br>Sugary beverages were the most common beverages sold in all outlets for the BCI companies' brands placed in an average of 25 locations in grocer y stores versus 15 for low-or no-calorie beverages, and 11 locations for water.<br>No difference in control or intervention stores in AL, CA and MS after 2 years.	**Social norm, policy and population health outcomes**<br>Not reported
**3. Social marketing campaigns** (n = 10 evaluations were identified for seven campaigns that discouraged sugary beverages or promoted water, low-fat or non-fat milk).					
Bonnevie et al. 2020[63]	***NJ Live Sugarfreed***<br>New Jersey (NJ) statewide (2017-2018)	**Population**: Low-income, Medicaid-eligible residents, especially African American	**Cognitive outcomes**<br>Passaic NJ residents showed a statistically	**Behavioral outcomes**<br>Baseline and follow-up surveys (n = 800	**Social norm, policy and population**

#NJSugarfreed	**Goal:** Reduce sugary beverage intake among low-income residents; educate about the health effects of sugary beverages; encourage people to choose water instead; and offer tips to switch to healthier beverages.	and Hispanic mothers and caregivers.<br><br>**Strategies:** Passaic County,<br>NJ received a higher dose intervention. Messaging disseminated through digital channels (i.e., Facebook, Instagram and Twitter) and websites for NJ Sugarfreed, Natural Beauty Sugarfreed and Sugarfreed Belleza.<br><br>**Theory or framework:** Collective impact model	significant increase in those who agreed that sugary beverages can have long-term health consequences for children (61% baseline; 70% follow-up, $p = 0.04$).<br><br>Baseline and follow-up surveys ($n = 800$ baseline; $n = 782$ f/u) showed increased knowledge about SSBs.	baseline; $n = 782$ f/u) showed positive trends toward decreased soda consumption. Passaic NJ respondents showed a 5% decrease in those who consume 1+ soda/day compared to a 1% decrease among NJ respondents.<br><br>**Retail outcomes**<br>Total sugary beverage sales showed greatest decrease in Passaic, NJ (7% decrease) compared to NJ statewide (6%).	**health outcomes**<br>Not reported
Farley et al. 2017[64]<br>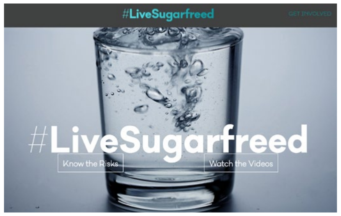 	***Live Sugarfreed***<br>Rural Kentucky (KY), Virginia (VA) and West Virginia (WV) for 15 weeks (2015-2016)<br><br>**Goal:** Reduce sugary beverage consumption.	**Population:** Adults aged 18-45 years, especially adults with highest reported sugary beverage consumption.<br><br>**Strategies:** Media messages through multiple channels. Video ads appeared on broadcast and cable television in the Tri-Cities designated market area and on the digital channels including YouTube and Hulu. Audio ads appeared on the Internet radio platform Pandora; and print ads were distributed via Hulu and Facebook.<br><br>**Theory or framework:**<br>Not reported	**Cognitive outcomes**<br>Post-campaign: 54% recalled seeing a campaign ad, and 53% believed sugary beverages caused heart disease and were more likely to view sugary beverages as a cause of diabetes (75% vs 60%; $p < .001$) after campaign.	**Behavioral outcomes**<br>Not reported<br><br>**Retail outcomes**<br>Compared with 12 months before and after the start of the campaign, sugary beverage sales decreased 3.4%, including a 4.1% decrease in soda sales in the intervention areas relative to comparison area ($p < .01$).	**Social norm, policy and population health outcomes**<br>Not reported
Hinckle et al. 2008[55]	***Adelante Con Leche Semi-descremada 1%***<br>Santa Paula (SP), CA rural 6 weeks (1998) and East Los Angeles (ELA), CA urban 8 weeks ((2000)	**Population:** Low-income Hispanic populations and milk vendors.<br><br>**Strategies:** Both SP and ELA used paid Spanish language	**Cognitive outcomes**<br>Formative research showed reported preference for whole milk (WM).	**Behavioral outcomes**<br>Not reported<br><br>**Retail outcomes**<br>Total milk sales increased ($p<0.001$) from baseline.	**Social norm, policy and population health outcomes**<br>Not reported

	**Goal:** Encourage 1% low-fat milk (LFM) or non-fat milk (NFM) sales and intake instead of whole milk (WM) in two Latino communities.	radio, newspaper and point of purchase ads; taste tests; community events and school-based programs. ELA same as SP plus paid television.<br><br>**Theory or framework:**<br>Not reported		SP found pre/post decrease in WM sales (p<.005) and increase in LFM sales (p<.001). In East LA, decrease in WM sales pre/post (p<0.001) but an increase six months later (p.<.013); and LFM sales increase not sustained.	
John et al. 2019[56]*<br><br>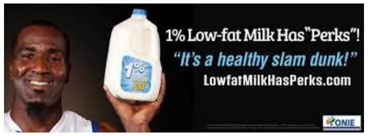<br>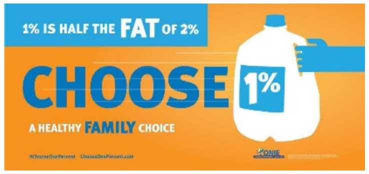	***1% Low-Fat Milk has Perks!***<br>Oklahoma City, OK<br>12 weeks (2012)<br>and<br>***Choose 1% Milk: A Health Family Choice***<br>Oklahoma statewide<br>5 weeks (2014)<br><br>**Goal:** Promote 1% LFM using two multi-level approaches.	**Population:** SNAP-eligible adults Oklahoma City and statewide.<br><br>**Strategies**: 4Ps (product, place, price, promotion) in both campaigns including English and Spanish TV commercials, radio and print ads; point-of-sale promotions; bus wraps, billboards and digital media.<br><br>Oklahoma City (2012) used Oklahoma NBA spokesperson Kendrick Perkins. Statewide (2014)<br><br>No spokesperson but female narrator and new social media used.<br><br>**Theory or framework:**<br>Social marketing framework	**Cognitive outcomes**<br>Formative research conducted on milk attitudes, knowledge and practices (not reported).<br><br>***1% Low-Fat Milk has Perks!*** (2012) campaign assessed by household telephone cross-sectional surveys among SNAP participants pre/post knowledge of LFM had improved.<br><br>No pre/post telephone survey for ***Choose 1% Milk*** (2014) campaign.	**Behavioral outcomes**<br>***1% Low-Fat Milk has Perks!*** Significant increase in self-reported consumption of 1% milk (from 4.1% to 7.9%), non-fat milk (from 3.6% to 4.7%) and NFM intake, coupled with a decrease in HFM intake (WM reduced by 10.2% and 2% milk reduced by 1.4% from baseline).<br><br>**Retail Outcomes**<br>**1% Low-Fat Milk has Perks!** Low-fat 1% milk sales increased from 10% to 11.5% of market share, translating to a relative change of a 15% increase vs no change in sales of low-fat milk comparison markets. Relative changes in WM sales: -4.6%, 2% milk +1.2% and NFM -2.0%.<br>**Choose 1% Milk** (2014). Milk sales data across OK state. Significant increase in market share of 1% milk sold from 7.1% to 10.1% or a 43% relative increase. Decreases in sales of whole milk (from 39.4% to 38.2%), 2% milk (from	**Social norm, policy and population health outcomes**<br>Not reported

				48.4% to 46.6%) and no change in NFM sales.	
Maddock et al. 2007[57]	***1% or Less** campaign*<br>Hawaii statewide<br>6 weeks (2004)<br><br>**Goal:** Encourage people to switch from high-fat to low-fat milk and maintain that behavior change over time in a culturally diverse state.	**Population:** Multi-ethnic Japanese, Native Hawaiians, Filipinos, Whites and Chinese.<br><br>**Strategies:** Paid radio and TV advertising; press conference launch and advisory commission; taste tests and community events; posters and website.<br><br>**Theory or framework:**<br>Theory of reasoned action	**Cognitive outcomes**<br>Among all taste-testers (*n*=323), 39.5% of correctly identified milk type.<br><br>*92*% reported liking low-fat 1% or skim milk.<br><br>87.1% of high-fat milk drinkers pledged to switch to low-fat milk after taste test.<br><br>Positive low-fat milk attitudes increased from baseline of 13.99 to 14.45 post-campaign (p<.01) and held at 3 months 14.42 (p<.01).<br><br>Non-significant trend (p=12) seen in precontemplation stage of change from 46% baseline to 41.6% at end and 3 months post campaign.	**Behavioral outcomes**<br>65.2% of taste-testers reported drinking high-fat milk at baseline.<br><br>Significant increase in reported consumption of LFM (1%) from 30% baseline to 41% after the campaign (p<0.001) and 36% at 3 months post campaign (p.<05).<br><br>Largest reduction in 2% milk consumption (45% at baseline, 37% immediate post-campaign, and 41% at 3-months post campaign).<br><br>**Retail outcomes**<br>Milk sales from largest milk distributor in Hawaii by region and ethnicity. Average LFM sales increased from 32.7% pre-campaign baseline in April/May to 39.9% at 3 months post campaign in Sept/Oct follow up. No sales data were collected in summer months.	**Social norm outcomes**<br>No changes in social norms were observed at the endpoint or 3 months after the campaign.<br><br>**Policy outcomes**<br>At the start of campaign, schools stopped offering 2% milk and replaced it with 1% milk.<br><br>**Population health outcomes**<br>Not reported
Reger et al. 1998[58]	***1% or Less***<br>Clarksburg and Bridgeport, WV 7 weeks from Feb–Apr (1995) compared to Wheeling, WV<br><br>**Goal:** Test the effectiveness of a community education "Media-Plus" campaign to encourage change from high-fat milk (2% or WM) to LFM.	**Population:** Residents in two WV cities (population 25,000 each) campaign reaching 280,000 people.<br><br>**Strategies:** Media-Plus pilot campaign of paid advertising (newspaper, TV, radio); public relations (press conferences and activities covered in press), and community education	**Cognitive outcomes**<br>94% of taste-test participants (*n* = 1910) reported liking either 1% LFM, NFM or both.	**Behavioral outcomes**<br>Pre (n=732)/ post (*n*=505) phone surveys showed 4 weeks post campaign, 38.2% of reported switching from HFM to LFM vs. 10.2% in the comparison (p.<0.00001).<br><br>48% of "2% only" milk drinkers reported switching to LFM as	**Social norm, policy and population health outcomes**<br>Not reported

		in schools, supermarkets, worksites and churches.<br><br>**Campaign costs:**<br>Costs for community education was $36,000 plus $24,000 for advertising averaged a cost per resident of $2.40 in intervention areas, of which. advertising was $0.96. Total persons reached cost was estimated at $0.22 per person.<br><br>**Theory or framework:**<br>Not reported		compared to 10.5% in the comparison city (p<0.00001).<br><br>36.4% of WM drinkers reported switching to LFM after campaign vs. 15.6% in the comparison (p<0.05).<br><br>**Retail outcomes**<br>In intervention cities, total volume of milk sold increased by 16% from baseline per supermarket per month in month following the campaign (p<0.05).<br><br>At six months, total volume increased by 25% from baseline to 9784 gallons per supermarket per month (p<0.01) vs. no significant changes in total milk sales in the comparison city.<br><br>Market share of LFM increased from 18% to 41% at end of the campaign and 35% six months later.	
Reger et al. 1999[59]	*1% or Less*<br>Wheeling, WV and comparison city Parkersburg, WV<br>6 weeks in Feb-Mar (1996)<br><br>**Goal:** Test a mass media campaign to produce a significant and sustained behavior change to replace drinking HFM with LFM.	Population: Residents in Wheeling (pop. 35,000) reaching 420,000 people.<br><br>**Strategies:** Mass media strategy of paid advertising (television, radio and newspaper) and public relations (press conferences, taste-tests and advisory board).	**Cognitive outcomes**<br>Not reported	**Behavioral outcomes**<br>34% of HFM drinkers reported switching to LFM in the intervention community vs. 3.6% in comparison (p<0.0001).<br><br>Most change occurred among those drinking 2% milk (44% of 2% milk drinkers in intervention city reported switching to	**Social norm, policy and population health outcomes**<br>Not reported

		(Note: same 1% or Less campaign as Bridgeport or Clarksberg pilot)/Telephone household panel surveys Pre- (n=740)/post (n=543) campaign.<br>**Costs** Total campaign $43, 000 translated into 10 cents/person).<br><br>**Theory or framework:**<br>Not reported		LFM as opposed to 3.2% (p. (p<0.0001).<br><br>**Retail outcomes**<br>LFM sales changed from 29% before campaign, to 46% in the month following the campaign, to 42% at 6-months following the campaign.<br>Volume of HFM sales decreased from 8135 gallons, to 6224, to 6134 in the month before the campaign, the month after and six months post campaign (p.<0.003), with no significant reductions in the comparison city (p<0.102).<br>No significant changes found (F<1.0) in total milk sales volume in gallons per supermarket per month between the intervention and comparison cities.	
Reger et al. 2000[60]	***1% or Less Campaign***<br>Parkersburg, WV<br>8 weeks in Feb-Mar (1997)<br>Beckeley, WV<br>6 weeks in Feb-Mar (1997)<br>Martinsburg, WV<br>Control in Feb-Mar (1997)<br><br>**Goal:** Promote switch from HFM to LFM intake and compare effectiveness of two different behavior change strategies with a control community.	**Population:** Middle-aged women residents (target) in rural communities.<br>Two intervention cities.<br>• Parkersburg WV Pop. 34,000<br>• Beckley, WV Pop. 18,000<br>One comparison city:<br>• Martinsburg, WV Pop. 14,000<br><br>**Strategies:**<br>Parkersburg: Public relations and community education<br>Beckley: Paid advertising only	**Cognitive outcomes**<br>Not reported	**Behavioral outcomes**<br>(Any) milk drinking at baseline; Parkersburg 87% Beckley 90% comparison 90% respondents who reported switching from HFM to LFM against comparison at endpoint.<br>Parkersburg: 19.6% (p<0.0001) and Beckley:12.8% (p<0.01)<br>Comparison: 6.8%<br><br>**Retail outcomes**<br>LFM sales in supermarkets (n = 21) in the month before/month after/ and six months after campaign.	**Social norm, policy and population health outcomes**<br>Not reported

		Parkersburg campaign cost $51,000 (~$1.50/person) and Beckley campaign cost $50,000 (~$2.70/person).<br><br>**Theory or framework:**<br>Not reported		No significant differences in overall milk sales or between intervention and comparison communities.<br>Parkersburg: 23%/28%/29%<br>Beckley: 28%/34%/27%<br>Comparison: 23%/22%/21%	
Wechsler and Wernick. 1992[61]	***Low-fat Milk Campaign***<br>Washington Heights-Inwood in New York City, NY<br>Phase 1: (Nov-Dec 1990)<br>Phase 2: (1991-1992)<br><br>**Goal:** Increase public awareness of and encourage institutional policies to promote LFM instead of HFM.	**Population:** Latina mothers with children aged 2-12 years.<br><br>**Strategies:**<br>***Phase 1:*** Distributed bilingual (English and Spanish) print (fliers, posters), community activities, and local media engagement.<br>***Phase 2:*** Persuaded local stores and institutions to promote LFM to residents and accept coupons.<br><br>**Theory or framework:**<br>Not reported	**Cognitive outcomes**<br>Not reported<br><br>Pre-campaign interviews with local store owners found preference for HFM. Pretesting of campaign messages and materials conducted with mothers. (Note not campaign outcomes)	**Behavioral outcomes**<br>Not reported<br><br>**Retail outcomes**<br>About 200 of 10,000 0.25 cent coupons to purchase low fat milk were redeemed in local supermarkets and bodegas (n=23). Note this rate is comparable to some commercial marketing campaigns.	**Social norm and population health outcomes**<br>Not reported<br><br>**Policy outcomes**<br>Day care and preschools (n=7) changed the institutional policy to offer only low LFM to children.
Wootan et al. 2005[62]	***1% or Less Campaign***<br>Clarksburg, WV (1996)<br>Wheeling, WV (1997)<br>Parkersburg, WV (1998)<br>Beckley, WV (1998)<br>6-8 week campaigns in Feb-Mar of respective years.<br><br>**Goal:** Promote behavior change to switch from HFM to LFM and compare cost-effectiveness of four different campaign strategies.	**Population:** Four WV communities.<br><br>**Strategies:** Clarksburg, WV: Paid ads, media relations and community education.<br>Wheeling, WV: Paid ads and media relations.<br>Parkersburg, WV: Paid ads and community education.<br>Beckley, WV: Paid ads only.<br>Cost/person for LFM switch:<br>Clarksburg: $0.73<br>Wheeling: $0.57<br>Parkersburg $11.85<br>Beckley: $1.56<br><br>**Theory or framework:**<br>Not reported	**Cognitive outcomes**<br>Not reported	**Behavioral outcomes**<br>Adults who reported switching from HFM to LFM at end of campaign based on household telephone survey pre (*n*=400)/post (*n*=280)<br>Clarksburg 38%/ 10%/p<.001<br>Wheeling 34%/ 4%/p<.001<br>Parkersburg 20%/ 7%/p<.001<br>Beckley 13%/ 7%/p=.01<br><br>**Retail outcomes**<br>LFM % of supermarket sales - (baseline/post campaign/6-month f/u	**Social norm, policy and population health outcomes**<br>Not reported

				/1-year f/u in all cities; 2-year f/u in Wheeling, WV only).<br>Clarksburg, WV (n=12)<br>• 18/41(p=0.003)/35 (p=0.017)/33<br>Wheeling, WV (n=12)<br>• 29/46 (p=0.013)/42/44/42<br>Parkersburg, WV (n= 12)<br>• 28/34/27/27 (NS)<br>Beckley, WV (n=7)<br>23/28/29/30 (NS)	
**4. Public information, awareness, education or health promotion campaigns** (*n* = 12 evaluations were identified for 11 campaigns used to discourage sugary beverage buying and intake and/or encourage water intake).					
Barragan et al. 2014[72]<br>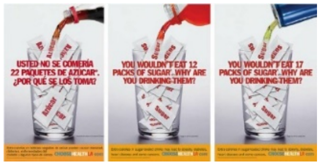	***Choose Health LA Sugar Pack***<br>Los Angeles County (LAC), CA<br>(2011-2012)<br><br>**Goal:** Change social norms to reduce consumer demand for sugary beverages.	**Population:** Adults were main target population and children were secondary target.<br><br>**Strategies:** Paid media placements on billboards, buses, railways and video on transit television (TV). Campaign was augmented using websites and social media platforms (i.e., Twitter, Facebook and YouTube).<br><br>**Theory or framework:**<br>Not reported	**Cognitive outcomes**<br><br>18.3% of 323 adults who did not see the campaign versus 38.8% of 596 adults who reported seeing the campaign accurately reported the number of sugar packets in a soda.<br><br>> 60% of 1,041 participants who completed the street survey reported likely or very likely to reduce their daily intake of sugary beverages due to seeing the campaign.	**Behavioral outcomes**<br>Not reported<br><br>**Retail outcomes**<br>Not reported	**Social norm, policy and population health outcomes**<br>Not reported
Bleakley et al. 2018[73]	***Get Healthy Philly***<br>Philadelphia, PA<br>(2011-2012)<br><br>**Goal:** Reduce sugary beverage intake as an obesity prevention strategy.	**Population:** Parents of children aged 3-16 years.<br><br>**Strategies:** Media (i.e., radio, transit, and web). Paper evaluated TV public service announcements (PSAs).<br><br>**Theory or framework:**<br>Theory of reasoned action	**Cognitive outcomes**<br>Exposure significantly associated with belief that reducing sugary beverage consumption decreased diabetes risk (P=.04) and was significantly negatively related to the belief that reducing sugary beverages would make meals less enjoyable (P=.04).	**Behavioral outcomes**<br>Not reported<br><br>**Retail outcomes**<br>Not reported	**Social norm, policy and population health outcomes**<br>Not reported

			Exposure to TV PSAs was significantly associated with intent to substitute non-sugary drinks for sugary drinks for parent (P=.04) and child (P=.02).		
Boehm et al. 2021[74]	***Howard County Unsweetened***<br>Howard County, MD (2012-2017)<br><br>**Goal:** Reduce consumption of sugary drinks of residents with a community-based campaign that used policy, systems, and environmental strategies	**Population:** Public school students in the sixth grade (n = 13,129) by race/ethnicity and/or community food environment.<br><br>**Strategies:** Community campaign of policy changes, media exposure, and community outreach. E.g., vending machine removal, strengthened nutrition standards for sales, serving only healthier beverages, digital marketing, cable TV commercials, direct mail, social media posts, and the "Better Beverage Finder" online tool. Event outreach by "Street teams" and healthcare providers encouraged to counsel patients on sugary drink consumption.<br><br>**Theory or framework:** Socio-ecological model	**Cognitive outcomes**<br>Not reported	**Behavioral outcomes**<br>For all students, the estimated daily calories from sugary drinks declined significantly from 220 at baseline in 2012/13 to 158 calories/day AT endpoint 2016/17, with a significant decline for reported daily sugary drink intake (49.4% to 6.9%).<br><br>Black (58.5%) and Hispanic (49.1%) youth students reported higher sugary drink intake in 2016/17 than Asian (22.5%), multiple/other race (37.7%) or white (33%) youth.	**Social norm, policy and population health outcomes**<br>Not reported
Boles et al. 2014[75]<br>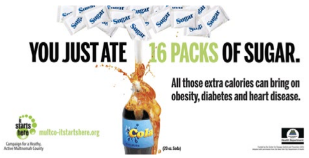 	***It Starts Here***<br>Multnomah County, Portland, OR (2011)<br><br>**Goal:** Educate about the sugar and calorie content of sugary beverages to raise awareness about how these products increase obesity.	**Population:** Women especially targeting mothers < 45 years.<br><br>**Strategies:** Paid and unpaid media on social media sites, TV, transit, billboards, one shopping mall, parks, recreation facilities, libraries and clinics, community advertising, and toolkits for use by community organizations.	**Cognitive outcomes**<br><br>85.9% of 125 respondents who were not aware of the campaign were more likely to agree that too much sugar caused health problems, compared with 97.3% of 277 respondents who were aware of the campaign.	**Behavioral outcomes**<br>No change in self-reported soda intake.<br><br>**Retail outcomes**<br>Not reported	**Social norm, policy and population health outcomes**<br>Not reported

		**Theory or framework:**<br>Behavior change theory	80% who were aware of the media campaign reported an intention to reduce the amount of sugary drinks they offered to a child due to the campaign ads.		
Caldwell et al. 2020[76]<br>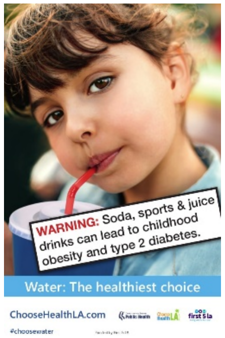	***Choose Water***<br>Los Angeles County, CA (LAC) (2015-2017)<br><br>**Goal:** Increase healthy beverage consumption in households.	**Population:** Parents of young children (*n* = 499).<br><br>**Strategies:** English and Spanish digital media, social media, radio, and out-of-home advertisements (in transit shelters and bus interiors).<br><br>**Theory or framework:**<br>Not reported	**Cognitive outcomes**<br>Parents who were exposed to and who discussed a campaign visual reported a greater intention to promote water intake and promote less sugary beverage intake than those who reported no campaign exposure.	**Behavioral outcomes**<br>A quarter (26%) of parents and 10.5% of low-education parents exposed to one or more campaign visual had discussed it with someone in their households.<br><br>**Retail outcomes**<br>Not reported	**Social norm, policy and population health outcomes**<br>Not reported
Hartigan et al. 2017[77]<br>rethink YOUR DRINK	***Rethink Your Drink***<br>San Diego, CA (2012-2013)<br><br>**Goal:** Reduce selection and sales of sugary beverages and increase non-sugary beverage choices in a children's hospital setting.	**Population:** Hospital employees, physicians, patients and visitors<br><br>**Strategies:** Multi-faceted interventions with print media (i.e., fliers, posters, handouts, table tents) that used a traffic light system (i.e., red, yellow and green).<br><br>Baseline (3 months Jan-Mar 2012); Intervention (12 months (April 2012-Mar 2013); Post-intervention (4 months: April-July 2013).<br><br>**Theory or framework:**<br>Not reported	**Cognitive outcomes**<br>Not reported	**Behavioral outcomes**<br>Not reported<br><br>**Retail outcomes**<br>Red beverage sales decreased ($p < 0.001$) from 56% at baseline to 32% at end; green beverage sales increased from 12.2% at baseline to 38%; and yellow beverages sales did not change ($p = 0.05$). Sales revenue for all drinks was constant.	**Social norm, policy and population health outcomes**<br>Not reported

Hornsby et al. 2017[78]	***Cavities Get Around***<br>Colorado statewide<br>Phase 1: 2011-2013<br>Phase II: 2014-present<br><br>**Goal:** Motivate families to limit children's fruit juice consumption and increase consumption of tap water to protect baby teeth from caries, while also building public will for children's oral health.	**Population:** All families (including low-income) with children aged 0–6 years.<br><br>**Strategies:** Targeted bilingual (English and Spanish) print and social media ads and education, community partnerships, policy engagement and educators.<br><br>**Theory or framework:**<br>Stages of change transtheoretical model	**Cognitive outcomes**<br>Decrease in percent of respondents who considered fruit juice consumption important to their child's health and nutritional needs (from 72% in 2014 to 43% in 2015) ($p < 0.01$).<br><br>Percent of parents considering baby teeth "less important" than adult teeth down from 21% in 2014 to 15% in 2015 ($p < 0.01$).	**Behavioral outcomes**<br>Percent of children regularly drinking tap water from up 41% in 2014 to 63% in 2015 ($p < 0.01$).<br><br>Reduced fruit juice consumption reported for young children from 66% in 2014 to 47% in 2015 ($p < 0.01$).<br><br>**Retail outcomes**<br>Not reported	**Social norm and population health outcomes**<br>Not reported<br><br>**Policy outcomes**<br>Campaign contributed to new state rules prohibiting childcare centers from serving sugary beverages and capping 100% juice to twice weekly.
James et al. 2020[79]<br>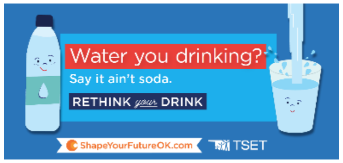 	***Shape Your Future – Rethink Your Drink***<br>Oklahoma statewide (2016-2017)<br><br>**Goal:** Educate people about the adverse health effects of sugary beverages and encourage them to limit sugary beverage intake.	**Population:** Parents and caregivers of children and adults with children living in the home.<br><br>**Strategies:** Combined print (posters), broadcast (i.e., radio, cable TV) and digital media.<br><br>**Theory or framework:**<br>Not reported	**Cognitive outcomes**<br>Among those with confirmed exposure, 76% reported sugary beverage consumption was linked to obesity, diabetes, and heart disease compared to 64% without confirmed exposure ($p = 0.0045$).	**Behavioral outcomes**<br>Sugary beverage intake decreased 18.6% ($p = 0.0232$) and heavy sugary beverage consumption (> 3/day) decreased 42.9% ($p = 0.0083$).<br>No differences in total sugary beverages by campaign exposure status.<br><br>**Retail outcomes**<br>Not reported	**Social norm, policy and population health outcomes**<br>Not reported
Maghrabi et al. 2021[80]	***Rethink Your Drink***<br>Rural City in SW Kentucky<br>4 weeks (2016)<br><br>**Goal:** Reduce sugary beverage consumption in the community to reduce obesity rates among adults.	**Population:** Adult volunteers (n=296) in *Rethink Your Drink* challenge.<br><br>**Strategies:** Campaign ran on local TV, radio and a website. Educational materials on beverage sugar content (categorized by red, yellow and green) and tracking tools provided to research partners in workplace human resource wellness initiatives. Volunteers filled in data sheets weekly.	**Cognitive outcomes**<br>Not reported	**Behavioral outcomes**<br>Adults reported sugary beverage intake decreased weeks 1-4<br>($p < 0.001$).<br>Average number of red (high sugar) beverages consumed weekly decreased (7 in week 1 to 3.2 drinks in week 4).<br>Average number of green (no sugar) beverages increased (21 in week 1 to 27 drinks in week 4).	**Social norm, policy and population health outcomes**<br>Not reported

		**Theory or framework:**<br>Not reported		**Retail outcomes**<br>Not reported	
Robles et al. 2015[81]<br>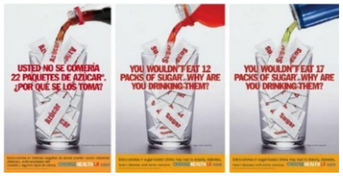	***Choose Health LA Sugar Pack***<br>Los Angeles County, CA (LAC) (2012)<br><br>**Goal:** Educate and encourage residents to reduce their sugary beverage consumption.	**Population:** LA County residents.<br><br>**Strategies:** English and Spanish ads in low-income areas with high obesity prevalence. (Metro) bus TVs, *Choose Health LA* website (www.choosehealthla.com) and social media (i.e., Twitter, Facebook, YouTube). Ads were disseminated in the bus and railway systems.<br><br>**Theory or framework:**<br>Theory of planned behavior	**Cognitive outcomes**<br><br>Respondents who were exposed to campaign significantly more likely to perceive harm from soda or other sugary drinks ($p<.01$).<br><br>Different patterns related to intention emerged by population sub-groups (i.e., whether moderate or heavy sugary beverage consumers, education levels and/or age).	**Behavioral outcomes**<br>Not reported<br><br>**Retail outcomes**<br>Not reported	**Social norm, policy and population health outcomes**<br>Not reported
Samuels & Associates 2010[82]<br>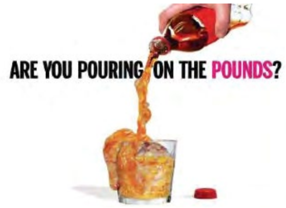<br>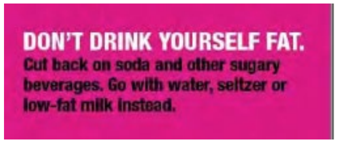	***Are You Pouring on the Pounds?***<br>San Francisco, CA (2010)<br><br>**Goal:** Motivate residents to eliminate or reduce sugary beverage consumption to prevent and reduce obesity.	**Population:** San Francisco residents.<br><br>**Strategies:** 400 ads were displayed on the interior of buses and 100 were posted on the exteriors during 2010 in Public Private Partnership.<br><br>(Blue Line Media donated 3 months of interior bus advertisement space to the San Francisco Department of Public Health that they purchased from the San Francisco Municipal Transit Agency. Additional advertising purchased by SFDPH to place the same posters on the exterior of buses for 6 weeks.)<br><br>No baseline. End-line Intercept Survey (n=318), two focus groups (n=17), and key informant interviews (n=7).	**Cognitive outcomes:**<br>No differences in beliefs about sugary beverages and obesity/health outcomes between respondents who saw the campaign and those who did not post-campaign.<br><br>Of the respondents who had seen the campaign, only 13% reported that they had intended to decrease their consumption of sugary beverages.<br><br>One-third of focus group of participants had seen the campaign. Many participants reported that the campaign message was not clear. Despite general support for a sugary beverage tax, some participants expressed that a	**Behavioral outcomes**<br>~60% of respondents reported infrequent intake of sugary beverages (across the three different data collection methods) post campaign.<br><br>No differences between survey respondents who saw the advertisement and those who did not in terms of sugary beverage consumption rates.<br><br>Some (2 out of 17 estimated) focus group participants said that the campaign made them think about sugary beverages and they then cut back on soda.<br><br>**Retail outcomes**<br>Not reported	**Social norm, policy and population health outcomes**<br>Not reported

		**Theory or framework:** Not reported	tax would not change behaviors.		
Schwartz et al. 2017[83]	***Howard County Unsweetened*** Howard County, MD (2013-2015)<br><br>**Goal:** Reduce sugary drinks intake of residents using policy, systems, and environmental strategies.	**Population:** Parents with children < 18 years.<br><br>**Strategies:** The community interventions included a multi-media campaign that disseminated ads through direct mail, outdoors, digital and social media platforms, cable television and broadcast media in child care centers, schools and health care settings. A 30-second PSA was aired called the Better Beverage Finder.org.<br><br>**Theory or framework:** Socio-ecological model	**Cognitive outcomes** Not reported	**Retail outcomes** 2012-2015: Regular soda sales in 15 Howard County (HC) stores decreased (-19.7%) and sales were stable (0.8%) in 17 control stores. Fruit drink sales decreased (15.3%) in HC stores and was stable (-0.6%) in comparison stores. Sales of 100% juice decreased more in HC (-15.0%) than comparison (-2.1%) stores Sales of sports drinks and diet soda decreased in both communities, but the decreases were not significantly different between groups.	**Social norm, policy and population health outcomes** Not reported
**5. Media advocacy or counter-marketing campaigns** (*n* = 1 evaluation was identified for The Bigger Picture campaign).					
Schillinger et al. 2018[86]	***The Bigger Picture*** San Francisco, CA (2013)<br><br>**Goal:** Encourage youth to reflect on how sugary beverages influence larger social, structural and environmental forces that shape behaviors and type 2 diabetes risk in an in-depth qualitative review.	**Population:** Low-income, ethnically and racially diverse youth (n= 13).<br><br>**Strategies:** Campaign website, PSAs disseminated via live high school assemblies, workshops and social media.<br><br>Youth filled out individual questionnaire and participated in a reflexive focus group.<br><br>**Theory or framework:** Not reported	**Cognitive outcomes**<br><br>Less than half (43%) of youth recognized key PSA messages. More in the focus groups (75%) identified type 2 diabetes in a public health literacy frame than (54%) for individual questionnaires. Four prominent themes emerged: Individual (choice to be healthy), environment (structural forces in the built environment), financial (in context of poverty and food insecurity, eating habits and choices	**Behavioral outcomes** Not reported<br><br>**Retail outcomes** Not reported	**Social norm, policy and population health outcomes** Not reported

			are limited), institutional (deceptive marketing).		
**6. Public policy or political media campaigns** (*n* = 0 evaluations were identified for sugary beverage tax campaigns to discourage sugary beverages).					

Abbreviations and Acronyms: Balance Calories Initiative (BCI); California (CA); Kentucky (KY); Los Angeles (LA); low-fat milk (LFM); Maryland (MD); New Jersey (NJ); New York (NY); New York City (NYC); non-fat milk (NFM); Oklahoma (OK); Oregon (OR); Pennsylvania (PA); public service announcement (PSA); San Francisco Department of Public Health (SFDPH); Santa Paula (SP); television (TV), Virginia (VA), whole milk (WM); West Virginia (WV).

* John et al. 2019 summarized relevant evidence from two earlier evaluations of the 1% Low-Fat Milk has Perks! Campaign implemented in Tulsa, OK and statewide. Therefore, Finnel et al. 2017 and Finnel and John 2018 were not included in the table.

References *(These numbers correspond with the published manuscript)*

51. Bogart LM, Castro G, Cohen DA. A qualitative exploration of parents', youths' and food establishment managers' perceptions of beverage industry self-regulation for obesity prevention. *Public Health Nutr.* 2019;22(5):805–813. https://doi.org/10.1017/S1368980018003865.
52. Cohen DA, Bogart L, Castro G, Rossi AD, Williamson S, Han B. Beverage marketing in retail outlets and The Balance Calories Initiative. *Prev Med.* 2018;115:1–7. https://doi.org/10.1016/j.ypmed.2018.07.014.
55. Hinckle AJ, Mistry R, McCarthy WJ, Yancey AK. Adapting a 1% or less milk campaign for a Hispanic/Latino population: the Adelante Con Leche Semi-descremada 1% experience. *Am J Health Promot.* 2008;23(2):108–111. http://dx.doi.org/10.4278/ajhp.07080780.
56. John R, Finnell KJ, Scott-Kaliki MS, DeBerry S.M. A case study of two successful social marketing interventions to promote 1% low-fat milk consumption. *Soc Market Quarter.* 2019;25(2):137–159. https://doi.org/10.1177/1524500418824292.
57. Maddock J, Maglione C, Barnett JD, Cabot C, Jackson S, Reger-Nash B. Statewide implementation of the 1% or Less Campaign. *Health Educ Behav.* 2007;34(6):953–963. http://dx.doi.org/10.1177/1090198106290621.
58. Reger B, Wootan MG, Booth-Butterfield S, Smith H. 1% or less: a community-based nutrition campaign. *Public Health Rep.* 1998;113(5):410–419. https://pubmed.ncbi.nlm.nih.gov/9769765.
59. Reger B, Wootan MG, Booth-Butterfield S. Using mass media to promote healthy eating: a community-based demonstration project. *Prev Med.* 1999;29(5):414–421. https://doi.org/10.1006/pmed.1998.0570.
60. Reger B, Wootan MG, Booth-Butterfield S. A comparison of different approaches to promote community-wide dietary change. *Am J Prev Med*. 2000;18(4):271–275. https://doi.org/10.1016/S0749-3797(00)00118-5.
61. Wechsler H, Wernick SM. A social marketing campaign to promote low-fat milk consumption in an inner-city Latino community. *Public Health Rep.* 1992;107(2):202–207. https://www.ncbi.nlm.nih.gov/pmc/articles/PMC1403632/.

62. Wootan MG, Reger-Nash B, Booth-Butterfield S, Cooper L. The cost-effectiveness of 1% or less media campaigns promoting low-fat milk consumption. *Prev Chronic Dis*. 2005;2(4):A05. http://www.ncbi.nlm.nih.gov/pmc/articles/PMC1435702/.
63. Bonnevie E, Morales O, Rosenberg SD, Goldbarg J, Silver M, Wartella E, Smyser J. Evaluation of a campaign to reduce consumption of sugar-sweetened beverages in New Jersey. *Prev Med.* 2020;136:106062. https://doi.org/10.1016/j.ypmed.2020.106062.
64. Farley T, Halper HS, Carlin AM, Emmerson KM, Foster KN, Fertig AR. Mass media campaign to reduce consumption of sugar-sweetened beverages in a rural area of the United States. *Am J Public Health*. 2017;107(6):989–995. https://doi.org/10.2105/AJPH.2017.303750.
72. Barragan NC, Noller AJ, Robles B et al. The "sugar pack" health marketing campaign in Los Angeles County, 2011-2012. *Health Promot Pract*. 2014;15(2):208–216. https://doi.org/10.1177/1524839913507280.
73. Bleakly A, Jordan, A, Mallya G, Hennessy M, Piotrowski JT. Do you know what your kids are drinking? Evaluation of a media campaign to reduce consumption of sugar-sweetened beverages. *Am J Health Promot*. 2018;32(6):1409–1416. https://doi.org/10.1177/0890117117721320.
74. Boehm R, Cooksey Stowers K, Schneider GE et al. Race, ethnicity, and neighborhood food environment are associated with adolescent sugary drink consumption during a 5-year community campaign. *J Racial Ethnic Health Disparities.* August 2021 (early release). https://doi.org/10.1007/s40615-021-01074-9.
75. Boles M, Adams A, Gredler A, Manhas S. Ability of a mass media campaign to influence knowledge, attitudes, and behaviors about sugary drinks and obesity. *Prev Med*. 2014;67(Suppl 1):S40–S45. https://doi.org/10.1016/j.ypmed.2014.07.023.
76. Caldwell JI, Robles B, Tyree R, Fraser RW, Dumke KA, Kuo T. Does exposure to the Choose Water campaign increase parental intentions to promote more water and less sugar-sweetened beverage consumption? *Am J Health Promot*. 2020;34(5):555–558. https://doi.org/10.1177%2F0890117120908785.
77. Hartigan P, Patton-Ku D, Fidler, C, Boutelle KN. Rethink Your Drink. *Health Promot Pract*. 2017;18(2):238–244. https://doi.org/10.1177/1524839915625215.
78. Hornsby WC, Bailey W, Braun PA, Weiss K, Heichelbech J. Busting the baby teeth myth and increasing children's consumption of tap water: building public will for children's oral health in Colorado. *Front Public Health.* 2017;5:238. https://doi.org/10.3389/fpubh.2017.00238.
79. James SA, White AH, Paulson SW, Beebe LA. Factors associated with sugar-sweetened beverage consumption in adults with children in the home after a statewide health communications program. *BMC Nutr*. 2020;6:23. https://doi.org/10.1186/s40795-020-00349-4.
80. Maghrabi P, Terry M. Effectiveness of a community-based health promotion “Rethink Your Drink” on reducing sugary beverage consumption: a case study. *J Exercise Nutr.* 2021;1(5):1–4. https://www.journalofexerciseandnutrition.com/index.php/JEN/article/view/24.
81. Robles B, Blitstein JL, Lieberman AJ, Barragan NC, Gase LN, Kuo T. The relationship between amount of soda consumed and intention to reduce soda consumption among adults exposed to the Choose Health LA 'Sugar Pack' health marketing campaign. *Public Health Nutr.* 2015;18(14):2582–2591. https://doi.org/10.1017/S1368980014003097.
82. Samuels & Associates. *Evaluation of San Francisco's Social Marketing Campaign “Pouring on the Pounds.”* California Obesity Prevention Program, 2010. Available online: https://www.iccp-portal.org/sites/default/files/multimediaresources/San%20Francisco_Pouring_on_the_Pounds_Report.pdf (accessed on 1 October 2021).

83. Schwartz MB, Schneider GE, Choi YY et al. Association of a community campaign for better beverage choices with beverage purchases from supermarkets. *JAMA Intern Med*. 2017;177(5):666–674. https://doi.org/10.1001/jamainternmed.2016.9650.
86. Schillinger D, Tran J, Fine S. Do low income youth of color see "*The Bigger Picture*" when discussing type 2 diabetes: a qualitative evaluation of a public health literacy campaign. *Int J Environ Res Public Health.* 2018;15(5):840. http://dx.doi.org/10.3390/ijerph15050840.

Fair Use Evaluation Documentation

Compiled using the **Fair Use Evaluator** [cc] 2008 Michael Brewer & the Office for Information Technology Policy, http://librarycopyright.net/fairuse/

**Name:**	Katherine Consavage Stanley, MS
**Job Title:**	PhD Student and Graduate Teaching Assistant
**Institution:**	Virginia Polytechnic Institute and State University
**Title of Work Used:**	Trademark or copyright images from U.S. beverage campaigns
**Copyright Holder:**	Global, national, regional and local companies and organizations, including multinational beverage companies, U.S.-based nonprofits and advocacy groups, and state and local governments, among others
**Publication Status:**	Published
**Publisher:**	Organizations who have funded or helped implement U.S. beverage campaigns
**Place of Publication:**	Internet and organization websites
**Publication Year:**	Varies based on campaign date
**Description of Work:**	The images will be included as part of two figures in a manuscript titled "A Systematic Scoping Review of Media Campaigns Used to Promote or Discourage Beverages in the United States to Reduce Sugary Beverage Health Risks for Americans"
**Date of Evaluation:**	October 5, 2021
**Date of Intended Use:**	October 5, 2021

Describe the **Purpose** **and Character of Your Intended Use:**

[+] Use is for "criticism, comment, news reporting, teaching, (including multiple print copies for classroom use), scholarship or research". The images used are a component of a systematic scoping review and analysis of U.S. beverage media campaigns conducted as part of a research study. The use is therefore solely for research.
[+] Use is transformative, i.e. it uses the existing work in a new way (creates an index to the work) or for a new purpose (parody, pastiche, instructional materials, etc.) and is socially beneficial. The study is innovative in its use of a media campaign typology to analyze and describe the landscape of U.S. beverage media campaigns. The findings of this study are used to suggest future actions and research needed to inform a social change movement to promote healthy hydration behaviors, with the aim of contributing to behavior change efforts to reduce sugary beverage health risks for Americans.
[+] Use is one-time, or is only occasional or spontaneous. The images will only be used in the published manuscript and for educational promotion of the manuscript on social media once published.
[+] Use is clearly defined, restricted in scope (limited duration, not iterative, restricted access, etc.), and not-for-profit. The use of these images is only in relation to specific U.S. beverage media campaigns identified as part of the systematic scoping review conducted for this study and is meant for illustrative purposes. The images will not be used

beyond the manuscript and educational promotion of the manuscript. The authors and published will not receive any monetary contribution for the use of these images.

Fair ⊢———————⊣ Unfair

☑ ☐ ☐ ☐ ☐ ☐ ☐

Describe the **Nature** **of Your Intended Use of the Copyrighted Work:**

[+] Work to be used has been previously PUBLISHED. The images being used in the manuscript have all been published elsewhere on the Internet and/or in print media, and they will be used solely asa companion to the text in the manuscript, in order to visually depict the messaging and images that accompanied select U.S. beverage campaigns.

Fair ⊢———————⊣ Unfair

☐ ☑ ☐ ☐ ☐ ☐ ☐

Describe the **Amount** **of Your Intended Use in Relation to the Copyrighted Work as a Whole:**

[+] The portion used is not the "heart" of the work (the portion considered most central to the work as a whole). The campaigns identified through the study's systematic scoping review, and their organization based on a media campaign typology, represents the central component of this manuscript. The copyrighted images will solely act as visual support for the campaigns described within the manuscript.
[+] Only the amount required to achieve the stated, socially-beneficial purpose or objective will be used (be that educational, artistic, scholarly, journalistic, etc.). The use of copyrighted images in the manuscript will be limited to only the quantity necessary to visually convey the findings of the study with regard to the landscape of U.S. beverage media campaigns and their categorization based on the media campaign typology. An educational promotion of the manuscript, once published, will only utilize the same images and figures included in the manuscript.

Fair ⊢———————⊣ Unfair

☐ ☑ ☐ ☐ ☐ ☐ ☐

Describe the **Effect** **of Your Intended Use on the Potential Market or Value of the Copyrighted Work:**

[+] Use of the work minimizes the potential for unauthorized use that could impact its value (i.e. steps are taken to ensure the content is not used outside of the stated purpose or audience). The copyrighted/trademarked images will only be used as part of the manuscript, which will be published in a peer-reviewed journal that's audience is academics and health professionals. The images will only be used for the purposes outlined within this fair use evaluation.
[+] Proper attribution will be given with the intended use

Fair — Unfair

☐ ☐ ☑ ☐ ☐ ☐ ☐

The Average "Fairness Level," Based on Your Rating of Each of the 4 Factors, Is:

[see tool disclaimer for important clarifying information]:

Fair — Unfair

☐ ☑ ☐ ☐ ☐ ☐ ☐

Based on the information and justification I have provided above, I, Katherine Consavage Stanley, MS, am asserting this use is **FAIR** under Section 107 of the U.S. Copyright Code.

Signature: Katherine Consavage Stanley

Date of Signature: 10/5/2021

Disclaimer: *This document is intended to help you* collect, organize & archive the information you might need to support your fair use evaluation. *It is not a source of legal advice or assistance. The results are only as good as the input you have provided by are intended to suggest next steps, and not to provide a final judgment. It is recommended that* you share this evaluation with a copyright specialist before proceeding with your intended use.
