# Supplementary material for: How have media campaigns been used to promote and discourage healthy and unhealthy beverages in the United States? A systematic scoping review to inform future research to reduce sugary beverage health risks
Source: Obes Rev. 2022 Feb 9;23(5):e13425. doi: 10.1111/obr.13425 (PMC9286342; doi:10.1111/obr.13425)
Supplement: Supplementary file 2 — Table S2: Comprehensive evidence summary of 24 evaluations for 20 unique U.S. beverage media campaigns organized by the typology category, goal, target population and outcomes, 1992–2021. [file OBR-23-0-s003.docx]

**Supplemental Table 2**

Comprehensive evidence summary of 24 evaluations for 20 unique U.S. beverage media campaigns organized by the typology category, goal, target population and outcomes, 1992-2021

Supplemental Table 2 provides detailed evidence for published evaluations (n=24) for 20 unique U.S. media campaigns oganized into four typology categories and describe the goal, objectives and target audiences; short-term outcomes (i.e., influence on cognitive outcomes including awareness, knowledge, attitudes and beliefs); mid-term outcomes (i.e., influence on retail policies, environments or individual behaviors); and long-term outcomes (i.e., influence on societal norms, values and population behaviors to educe obesity and diet-related non-communicable dieases including type 2 diabetes and cardiovascular diseases).

| **Typology Category** | ***Campaign name***  Location (city, state)  time frame  **Goal** | **Target population**  **Strategies**  **Theory or conceptual framework used to plan the campaign** | **Short-term outcomes**  **Cognitive outcomes** (i.e., awareness, attitudes, beliefs, knowledge or preferences) | **Mid-term outcomes**  **Behavioral outcomes**  (i.e., reported or measured individual behaviors)  **Retail outcomes**  (revenue or sales) | **Long-term outcomes**  **Social norm, policy and population health outcomes** (i.e., institutional policy change, weight, obesity or diabetes) |
| --- | --- | --- | --- | --- | --- |
| **1. Corporate advertising, marketing or entertainment branded campaigns** (n *=* 0 evaluations were identified for sugary beverage brands or products) | | | | | |
| **2. Corporate social responsibility, public relations, cause marketing campaigns** (*n* = 2 evaluations were identified for the Balance Calories Initiative campaign) | | | | | |
| Bogart et al. 2019^51^  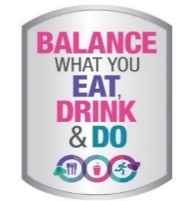  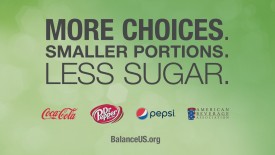 | ***Balance Calories Initiative***  Montgomery, AL; North  Mississippi Delta, MS; and  Eastern Los Angeles, CA  (2016-2017)  **Goal:** Decrease per capita intake of energy from beverages by 20% by 2025. | **Population:** Low-income  communities (*n* = 8-10)  with low access to  reduced-calorie or  no-calorie beverages.  **Strategies:** The American  Beverage Association’s  (ABA’s) BCI introduced  and expanded reduced-calorie products and smaller-portion packages; changed product  placement (e.g., end-aisle and  checkout displays featuring  only reduced-calorie beverages,  repositioning reduced  calorie beverages on  shelves); provided  coupons and promotions  for reduced- and no-calorie  options; conducted taste tests;  and promoted energy balance  messages on beverage coolers  and billboards.  **Theory or framework:**  Not reported | **Cognitive outcomes**  Parents (*n* = 12) and youth (*n* = 24) saw the BCI messages. Many parents and youth misunderstood the BCI messages, interpreted that they should drink *more* sugary beverages or that they needed to equalize healthy and unhealthy beverage intake.  Store managers (*n* = 4) were aware of BCI and (*n* = 1) had communicated with firms about BCI. | **Behavioral or retail outcomes**  Not reported | **Social norm, policy and population health outcomes**  Not reported |
| Cohen et al. 2018^52^  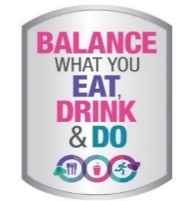  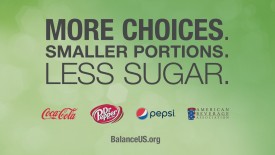 | ***Balance Calories Initiative***  Montgomery, AL; North  Mississippi Delta, MS; and  Eastern Los Angeles, CA  (2016-2017)  **Goal:** Decrease per capita  intake of energy from  beverages by 20% by 2025. | **Population:** Low-income  communities (*n* = 8-10) with  low access to reduced-calorie or  no-calorie beverages.  **Strategies:** Product  placement in low-income  communities featured  merchandising, couponing  and other incentives; and  more intensive efforts to  reduce SSB consumption  in the selected communities.  **Theory or framework:**  Not reported | **Cognitive outcomes**  Not reported | **Behavioral outcomes**  Not reported  **Retail outcomes**  Sugary beverages were the most common beverages sold in all outlets for the BCI companies’ brands placed in an average of 25 locations in grocer y stores versus 15 for low-or no-calorie beverages, and 11 locations for water.  No difference in control or intervention stores in AL, CA and MS after 2 years. | **Social norm, policy and population health outcomes** Not reported |
| **3. Social marketing campaigns** (*n* = 10 evaluations were identified for seven campaigns that discouraged sugary beverages or promoted water, low-fat or non-fat milk). | | | | | |
| Bonnevie et al. 2020^63^  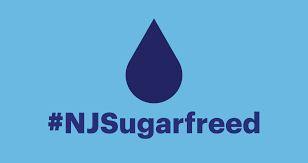 | ***NJ Live Sugarfreed***  New Jersey (NJ) statewide  (2017-2018)  **Goal:** Reduce sugary beverage intake among low-income residents; educate about the health effects of sugary beverages; encourage people to choose water instead; and offer tips to switch to healthier beverages. | **Population**: Low-income, Medicaid-eligible residents, especially African American and Hispanic mothers and caregivers.  **Strategies:** Passaic  County,  NJ received a higher dose  intervention. Messaging  disseminated through  digital channels (i.e.,  Facebook, Instagram and  Twitter) and websites for  NJ Sugarfreed, Natural  Beauty Sugarfreed and  Sugarfreed Belleza.  **Theory or framework:** Collective impact model | **Cognitive outcomes** Passaic NJ residents showed a statistically significant increase in those who agreed that sugary beverages can have long-term health consequences for children (61% baseline; 70% follow-up, p = 0.04).  Baseline and follow-up surveys (*n* = 800  baseline; *n* = 782 f/u) showed increased knowledge about SSBs. | **Behavioral outcomes**  Baseline and follow-up surveys (*n* = 800  baseline; *n* = 782 f/u) showed positive trends toward decreased soda consumption. Passaic NJ respondents showed a 5% decrease in those who consume 1+ soda/day compared to a 1% decrease among NJ respondents.  **Retail outcomes**  Total sugary beverage sales showed greatest decrease in Passaic, NJ (7% decrease) compared to NJ statewide (6%). | **Social norm, policy and population health outcomes** Not reported |
| Farley et al. 2017^64^  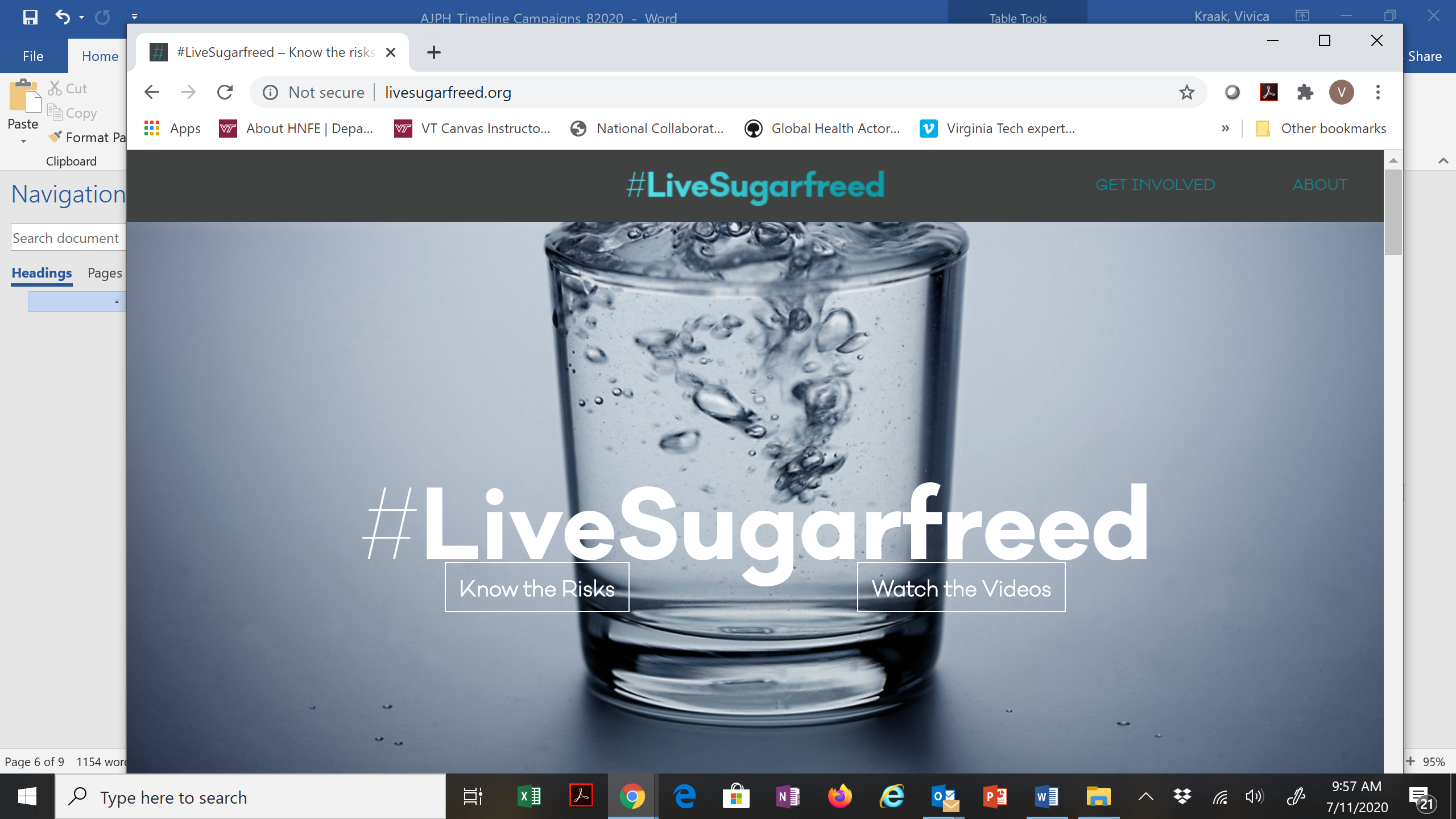 | ***Live Sugarfreed***  Rural Kentucky (KY),  Virginia (VA) and West  Virginia (WV) for 15 weeks  (2015-2016)  **Goal:** Reduce sugary beverage consumption. | **Population:** Adults aged 18-45 years, especially adults with highest reported sugary beverage consumption.  **Strategies:** Media messages through multiple channels.  Video ads appeared on broadcast and cable television in the Tri-Cities designated market area and on the digital channels including YouTube and Hulu. Audio ads appeared on the Internet radio platform Pandora; and print ads were distributed via Hulu and Facebook.  **Theory or framework:**  Not reported | **Cognitive outcomes**  Post-campaign: 54% recalled seeing a campaign ad, and 53% believed sugary beverages caused heart disease and were more likely to view sugary beverages as a cause of diabetes (75% vs 60%;  p <.001) after campaign. | **Behavioral outcomes**  Not reported  **Retail outcomes**  Compared with 12 months before and after the start of the campaign, sugary beverage sales decreased 3.4%, including a 4.1% decrease in soda sales in the intervention areas relative to comparison area (p < .01). | **Social norm, policy and population health outcomes** Not reported |
| Hinckle et al. 2008^55^ | ***Adelante Con Leche***  ***Semi-descremada 1%***  Santa Paula (SP), CA rural  6 weeks (1998) and  East Los Angeles (ELA),  CA urban 8 weeks ((2000)  **Goal:** Encourage 1% low-fat milk (LFM) or non-fat milk (NFM) sales and intake instead of whole milk (WM) in two Latino communities. | **Population:** Low-income Hispanic populations and milk vendors.  **Strategies:** Both SP and ELA used paid Spanish language radio, newspaper and point of purchase ads; taste tests; community events and school-based programs. ELA same as SP plus paid television.  **Theory or framework:**  Not reported | **Cognitive outcomes** Formative research showed reported preference for whole milk (WM). | **Behavioral outcomes**  Not reported  **Retail outcomes**  Total milk sales increased (p<0.001) from baseline. SP found pre/post decrease in WM sales (p<.005) and increase in LFM sales (p<.001). In East LA, decrease in WM sales pre/post (p<0.001) but an increase six months later (p.<.013); and LFM sales increase not sustained. | **Social norm, policy and population health outcomes**  Not reported |
| John et al. 2019^56^*  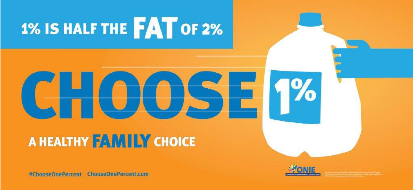*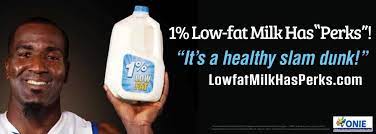* | ***1% Low-Fat Milk has Perks!***  Oklahoma City, OK  12 weeks (2012)  and  ***Choose 1% Milk: A Health Family Choice***  Oklahoma statewide  5 weeks (2014)  **Goal:** Promote 1% LFM using two multi-level approaches. | **Population:** SNAP-eligible adults Oklahoma City and statewide.  **Strategies**: 4Ps (product, place, price, promotion) in both campaigns including English and Spanish TV commercials, radio and print ads; point-of-sale promotions; bus wraps, billboards and digital media.    Oklahoma City (2012) used Oklahoma NBA spokesperson Kendrick Perkins. Statewide (2014)  No spokesperson but female narrator and new social media used.  **Theory or framework:**  Social marketing framework | **Cognitive outcomes**  Formative research conducted on milk attitudes, knowledge and practices (not reported).  ***1% Low-Fat Milk has Perks!* (**2012) campaign assessed by household telephone cross-sectional surveys among SNAP participants pre/post  knowledge of LFM had improved.  No pre/post telephone survey for ***Choose 1% Milk*** (2014) campaign. | **Behavioral outcomes**  ***1% Low-Fat Milk has Perks!*** Significant increase in self-reported consumption of 1% milk (from 4.1% to 7.9%), non-fat milk (from 3.6% to 4.7%) and NFM intake, coupled with a decrease in HFM intake (WM reduced by 10.2% and 2% milk reduced by 1.4% from baseline).  **Retail Outcomes**  **1% Low-Fat Milk has Perks!** Low-fat 1% milk sales increased from 10% to 11.5% of market share, translating to a relative change of a 15% increase vs no change in sales of low-fat milk comparison markets. Relative changes in WM sales: -4.6%, 2% milk +1.2% and NFM -2.0%.  **Choose 1% Milk** (2014). Milk sales data across OK state. Significant increase in market share of 1% milk sold from 7.1% to 10.1% or a 43% relative increase. Decreases in sales of whole milk (from 39.4% to 38.2%), 2% milk (from 48.4% to 46.6%) and no change in NFM sales. | **Social norm, policy and population health outcomes**  Not reported |
| Maddock et al. 2007^57^  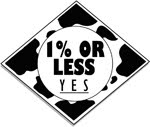 | ***1% or Less*** *campaign*  Hawaii statewide  6 weeks (2004)  **Goal:** Encourage people to switch from high-fat to low-fat milk and maintain that behavior change over time in a culturally diverse state. | **Population:** Multi-ethnic Japanese, Native Hawaiians, Filipinos, Whites and Chinese.  **Strategies:** Paid radio and TV advertising; press conference launch and advisory commission; taste tests and community events; posters and website.  **Theory or framework:**  Theory of reasoned action | **Cognitive outcomes**  Among all taste-testers (*n*=323), 39.5% of correctly identified milk type.  *92*% reported liking low-fat 1% or skim milk.  87.1% of high-fat milk drinkers pledged to switch to low-fat milk after taste test.  Positive low-fat milk attitudes increased from baseline of 13.99 to 14.45 post-campaign (p<.01) and held at 3 months 14.42 (p<.01).  Non-significant trend (p=12) seen in precontemplation stage of change from 46% baseline to 41.6% at end and 3 months post campaign. | **Behavioral outcomes**  65.2% of taste-testers reported drinking high-fat milk at baseline.  Significant increase in reported consumption of LFM (1%) from 30% baseline to 41% after the campaign (p<0.001) and 36% at 3 months post campaign (p.<05).    Largest reduction in 2% milk consumption (45% at baseline, 37% immediate post-campaign, and 41% at 3-months post campaign).  **Retail outcomes**  Milk sales from largest milk distributor in Hawaii by region and ethnicity. Average LFM sales increased from 32.7% pre-campaign baseline in April/May to 39.9% at 3 months post campaign in Sept/Oct follow up. No sales data were collected in summer months. | **Social norm outcomes**  No changes in social norms were observed at the endpoint or 3 months after the campaign.  **Policy outcomes**  At the start of campaign, schools stopped offering 2% milk and replaced it with 1% milk.  **Population health outcomes**  Not reported |
| Reger et al. 1998^58^  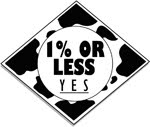 | ***1% or Less***  Clarksburg and Bridgeport, WV 7 weeks from Feb–Apr (1995) compared to Wheeling, WV  **Goal:** Test the effectiveness of a community education “Media-Plus” campaign to encourage change from high-fat milk (2% or WM) to LFM. | **Population:** Residents in two WV cities (population 25,000 each) campaign reaching 280,000 people.  **Strategies:** Media-Plus  pilot campaign of paid  advertising (newspaper,  TV, radio); public relations  (press conferences and  activities covered in press),  and community education  in schools, supermarkets,  worksites and churches.  **Campaign costs:**  Costs for community education was $36,000 plus $24,000 for advertising averaged a cost per resident of $2.40 in intervention areas, of which. advertising was $0.96. Total persons reached cost was estimated at $0.22 per person.  **Theory or framework:**  Not reported | **Cognitive outcomes**  94% of taste-test participants (*n* = 1910) reported liking either 1% LFM, NFM or both. | **Behavioral outcomes**  Pre (n=732)/ post (*n*=505) phone surveys showed 4 weeks post campaign, 38.2% of reported switching from HFM to LFM vs. 10.2% in the comparison (p.<0.00001).  48% of “2% only” milk drinkers reported switching to LFM as compared to 10.5% in the comparison city (p<0.00001).  36.4% of WM drinkers reported switching to LFM after campaign vs. 15.6% in the comparison (p<0.05).  **Retail outcomes**  In intervention cities, total volume of milk sold increased by 16% from baseline per supermarket per month in month following the campaign (p<0.05).  At six months, total volume increased by 25% from baseline to 9784 gallons per supermarket per month (p<0.01) vs. no significant changes in total milk sales in the comparison city.  Market share of LFM increased from 18% to 41% at end of the campaign and 35% six months later. | **Social norm, policy and population health outcomes**  Not reported |
| Reger et al. 1999^59^  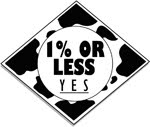 | ***1% or Less***  Wheeling, WV and comparison city Parkersburg, WV  6 weeks in Feb-Mar (1996)  **Goal:** Test a mass media campaign to produce a significant and sustained behavior change to replace drinking HFM with LFM. | Population: Residents in Wheeling (pop. 35,000) reaching 420,000 people.  **Strategies:** Mass media strategy of paid advertising (television, radio and newspaper) and public relations (press conferences, taste-tests and advisory board).  (Note: same 1% or Less campaign as Bridgeport or Clarksberg pilot)/Telephone household panel surveys Pre- (n=740)/post (n=543) campaign.  **Costs** Total campaign $43, 000 translated into 10 cents/person).  **Theory or framework:**  Not reported | **Cognitive outcomes**  Not reported | **Behavioral outcomes**  34% of HFM drinkers reported switching to LFM in the intervention community vs. 3.6% in comparison (p<0.0001).  Most change occurred among those drinking 2% milk (44% of 2% milk drinkers in intervention city reported switching to LFM as opposed to 3.2% (p. (p<0.0001).  **Retail outcomes**  LFM sales changed from 29% before campaign, to 46% in the month following the campaign, to 42% at 6-months following the campaign.  Volume of HFM sales decreased from 8135 gallons, to 6224, to 6134 in the month before the campaign, the month after and six months post campaign (p.<0.003), with no significant reductions in the comparison city (p<0.102).  No significant changes found (F<1.0) in total milk sales volume in gallons per supermarket per month between the intervention and comparison cities. | **Social norm, policy and population health outcomes**  Not reported |
| Reger et al. 2000^60^  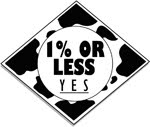 | ***1% or Less Campaign***  Parkersburg, WV  8 weeks in Feb-Mar (1997)  Beckeley, WV  6 weeks in Feb-Mar (1997)  Martinsburg, WV  Control in Feb-Mar (1997)  **Goal:** Promote switch from HFM to LFM intake and compare effectiveness of two different behavior change strategies with a control community. | **Population:** Middle-aged women residents (target) in rural communities.  Two intervention cities.   - Parkersburg WV Pop. 34,000 - Beckley, WV   Pop. 18,000  One comparison city:   - Martinsburg, WV   Pop. 14,000  **Strategies:**  Parkersburg: Public relations and community education  Beckley: Paid advertising only  Parkersburg campaign cost $51,000 (~$1.50/person) and Beckley campaign cost $50,000 (~$2.70/person).  **Theory or framework:**  Not reported | **Cognitive outcomes**  Not reported | **Behavioral outcomes**  (Any) milk drinking at baseline; Parkersburg 87% Beckley 90% comparison 90% respondents who reported switching from HFM to LFM against comparison at endpoint.  Parkersburg: 19.6% (p<0.0001) and Beckley:12.8% (p<0.01)  Comparison: 6.8%  **Retail outcomes**  LFM sales in supermarkets (n = 21) in the month before/month after/ and six months after campaign.  No significant differences in overall milk sales or between intervention and comparison communities.  Parkersburg: 23%/28%/29%  Beckley: 28%/34%/27%  Comparison: 23%/22%/21% | **Social norm, policy and population health outcomes**  Not reported |
| Wechsler and Wernick. 1992^61^ | ***Low-fat Milk Campaign***  Washington Heights-Inwood in New York City, NY  Phase 1: (Nov-Dec 1990)  Phase 2: (1991-1992)  **Goal:** Increase public awareness of and encourage institutional policies to promote LFM instead of HFM. | **Population:** Latina mothers with children aged 2-12 years.  **Strategies:**  ***Phase 1:*** Distributed bilingual (English and Spanish) print (fliers, posters), community activities, and local media engagement.  ***Phase 2:*** Persuaded local stores and institutions to promote LFM to residents and accept coupons.  **Theory or framework:**  Not reported | **Cognitive outcomes**  Not reported  Pre-campaign interviews with local store owners found preference for HFM. Pretesting of campaign messages and materials conducted with mothers. (Note not campaign outcomes) | **Behavioral outcomes**  Not reported  **Retail outcomes**  About 200 of 10,000 0.25 cent coupons to purchase low fat milk were redeemed in local supermarkets and bodegas (n=23). Note this rate is comparable to some commercial marketing campaigns. | **Social norm and population health outcomes**  Not reported  **Policy outcomes**  Day care and preschools (n=7) changed the institutional policy to offer only low LFM to children. |
| Wootan et al. 2005^62^  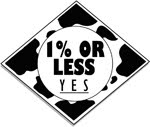 | ***1% or Less Campaign***  Clarksburg, WV (1996)  Wheeling, WV (1997)  Parkersburg, WV (1998)  Beckley, WV (1998)  6-8 week campaigns in  Feb-Mar of respective years.  **Goal:** Promote behavior change to switch from HFM to LFM and compare cost-effectiveness of four different campaign strategies. | **Population:** Four WV communities.  **Strategies:** Clarksburg, WV: Paid ads, media relations and community education.  Wheeling, WV: Paid ads and media relations.  Parkersburg, WV: Paid ads and community education.  Beckley, WV: Paid ads only.  Cost/person for LFM switch:  Clarksburg: $0.73  Wheeling: $0.57  Parkersburg $11.85  Beckley: $1.56  **Theory or framework:**  Not reported | **Cognitive outcomes**  Not reported | **Behavioral outcomes**  Adults who reported switching from HFM to LFM at end of campaign based on household telephone survey pre (*n*=400)/post (*n*$=$280)  Clarksburg 38%/ 10%/p<.001  Wheeling 34%/ 4%/p<.001  Parkersburg 20%/ 7%/p<.001  Beckley 13%/ 7%/p=.01  **Retail outcomes**  LFM % of supermarket sales - (baseline/post campaign/6-month f/u  /1-year f/u in all cities; 2-year f/u in Wheeling, WV only).  Clarksburg, WV (n=12)   - 18/41(p=0.003)/35 (p=0.017)/33   Wheeling, WV (n=12)   - 29/46 (p=0.013)/42/44/42   Parkersburg, WV (n= 12)   - 28/34/27/27 (NS)   Beckley, WV (n=7)  23/28/29/30 (NS) | **Social norm, policy and population health outcomes**  Not reported |
| **4. Public information, awareness, education or health promotion campaigns** (*n* = 12 evaluations were identified for 11 campaigns used to discourage sugary beverage buying and intake and/or encourage water intake). | | | | | |
| Barragan et al. 2014^72^  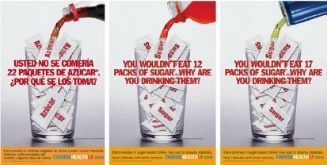 | ***Choose Health LA***  ***Sugar Pack***  Los Angeles County (LAC),  CA  (2011-2012)  **Goal:** Change social norms to reduce consumer demand for sugary beverages. | **Population:** Adults were main target population and children were secondary target.  **Strategies:** Paid media placements on billboards, buses, railways and video on transit television (TV). Campaign was augmented using websites and social media platforms (i.e., Twitter, Facebook and YouTube).  **Theory or framework:**  Not reported | **Cognitive outcomes**  18.3% of 323 adults who did not see the campaign versus 38.8% of 596 adults who reported seeing the campaign accurately reported the number of sugar packets in a soda.  > 60% of 1,041 participants who completed the street survey reported likely or very likely to reduce their daily intake of sugary beverages due to seeing the campaign. | **Behavioral outcomes**  Not reported  **Retail outcomes**  Not reported | **Social norm, policy and population health outcomes** Not reported |
| Bleakley et al. 2018^73^  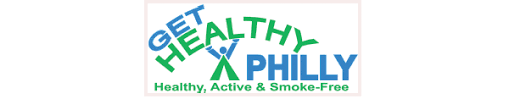 | ***Get Healthy Philly***  Philadelphia, PA  (2011-2012)  **Goal:** Reduce sugary  beverage intake as an  obesity prevention strategy. | **Population:** Parents of children aged 3-16 years.  **Strategies:** Media (i.e., radio, transit, and web). Paper evaluated TV public service announcements (PSAs).  **Theory or framework:**  Theory of reasoned action | **Cognitive outcomes**  Exposure significantly associated with belief that reducing sugary beverage consumption decreased diabetes risk (P=.04) and was significantly negatively related to the belief that reducing sugary beverages would make meals less enjoyable (P=.04).  Exposure to TV PSAs was significantly associated with intent to substitute non-sugary drinks for sugary drinks for parent (P=.04) and child (P=.02). | **Behavioral outcomes**  Not reported  **Retail outcomes**  Not reported | **Social norm, policy and population health outcomes**  Not reported |
| Boehm et al. 2021^74^  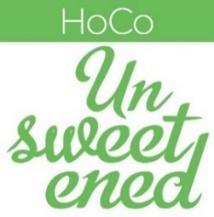 | ***Howard County Unsweetened***  Howard County, MD  (2012-2017)  **Goal:** Reduce consumption of sugary drinks of residents with a community-based campaign that used policy, systems, and environmental strategies | **Population:** Public school students in the sixth grade  (n = 13,129) by race/ethnicity and/or community food environment.  **Strategies:** Community campaign of policy changes, media exposure, and community outreach. E.g., vending machine removal, strengthened nutrition standards for sales, serving only healthier beverages, digital marketing, cable TV commercials, direct mail, social media posts, and the “Better Beverage Finder” online tool. Event outreach by “Street teams” and healthcare providers encouraged to counsel patients on sugary drink consumption.  **Theory or framework:**  Socio-ecological model | **Cognitive outcomes**  Not reported | **Behavioral outcomes**  For all students, the estimated daily calories from sugary drinks declined significantly from 220 at baseline in 2012/13 to 158 calories/day AT endpoint 2016/17, with a significant decline for reported daily sugary drink intake (49.4% to 6.9%).  Black (58.5%) and Hispanic (49.1%) youth students reported higher sugary drink intake in 2016/17 than Asian (22.5%), multiple/other race (37.7%) or white (33%) youth. | **Social norm, policy and population health outcomes**  Not reported |
| Boles et al. 2014^75^  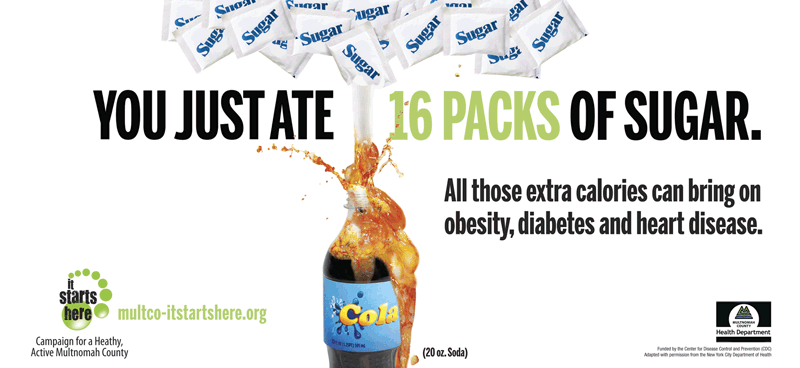 | ***It Starts Here***  Multnomah County,  Portland, OR (2011)  **Goal:** Educate about the sugar and calorie content of sugary beverages to raise awareness about how these products increase obesity. | **Population:** Women especially targeting mothers < 45 years.  **Strategies:** Paid and unpaid media on social media sites, TV, transit, billboards, one shopping mall, parks, recreation facilities, libraries and clinics, community advertising, and toolkits for use by community organizations.  **Theory or framework:**  Behavior change theory | **Cognitive outcomes**  85.9% of 125 respondents who were not aware of the campaign were more likely to agree that too much sugar caused health problems, compared with 97.3% of 277 respondents who were aware of the campaign.  80% who were aware of the media campaign reported an intention to reduce the amount of sugary drinks they offered to a child due to the campaign ads. | **Behavioral outcomes**  No change in self-reported soda intake.  **Retail outcomes**  Not reported | **Social norm, policy and population health outcomes**  Not reported |
| Caldwell et al. 2020^76^  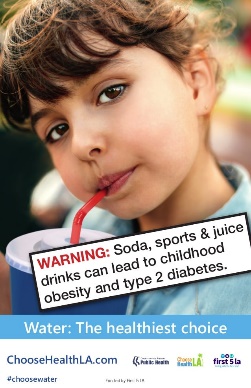 | ***Choose Water***  Los Angeles County, CA (LAC) (2015-2017)  **Goal:** Increase healthy beverage consumption in households. | **Population:** Parents of young children (*n* = 499).  **Strategies:** English and Spanish digital media, social media, radio, and out-of-home advertisements (in transit shelters and bus interiors).  **Theory or framework:**  Not reported | **Cognitive outcomes**  Parents who were exposed to and who discussed a campaign visual reported a greater intention to promote water intake and promote less sugary beverage intake than those who reported no campaign exposure. | **Behavioral outcomes**  A quarter (26%) of parents and 10.5% of low-education parents exposed to one or more campaign visual had discussed it with someone in their households.  **Retail outcomes**  Not reported | **Social norm, policy and population health outcomes**  Not reported |
| Hartigan et al. 2017^77^  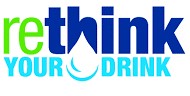 | ***Rethink Your Drink***  San Diego, CA  (2012-2013)  **Goal:** Reduce selection and  sales of sugary beverages  and increase non-sugary  beverage choices in a  children’s hospital setting. | **Population:** Hospital employees, physicians, patients and visitors  **Strategies:** Multi-faceted interventions with print media (i.e., fliers, posters, handouts, table tents) that used a traffic light system (i.e., red, yellow and green).  Baseline (3 months Jan-Mar 2012); Intervention (12 months (April 2012-Mar 2013); Post-intervention (4 months: April-July 2013).  **Theory or framework:**  Not reported | **Cognitive outcomes**  Not reported | **Behavioral outcomes**  Not reported  **Retail outcomes**  Red beverage sales decreased (p <0.001) from 56% at baseline to 32% at end; green beverage sales increased from 12.2% at baseline to 38%; and yellow beverages sales did not change (p = 0.05).  Sales revenue for all drinks was constant. | **Social norm, policy and population health outcomes** Not reported |
| Hornsby et al. 2017^78^  *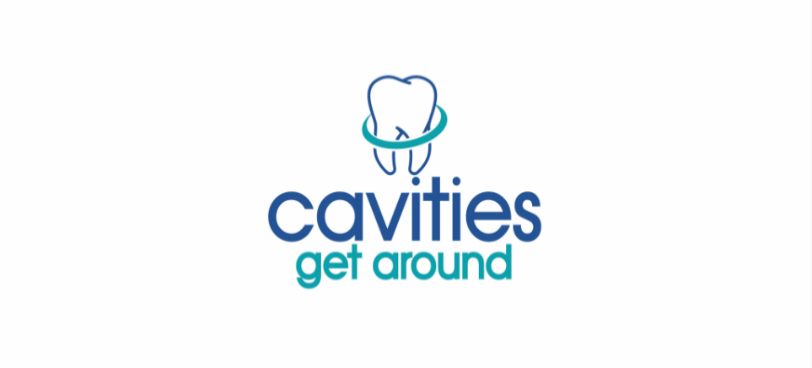* | ***Cavities Get Around***  Colorado statewide  Phase 1: 2011-2013  Phase II: 2014-present  **Goal:** Motivate families to limit children’s fruit juice consumption and increase consumption of tap water to protect baby teeth from caries, while also building public will for children’s oral health. | **Population:** All families (including low-income) with children aged 0–6 years.  **Strategies:** Targeted bilingual (English and Spanish) print and social media ads and education, community partnerships, policy engagement and educators.  **Theory or framework:**  Stages of change transtheoretical model | **Cognitive outcomes**  Decrease in percent of respondents who considered fruit juice consumption important to their child’s health and nutritional needs (from 72% in 2014 to 43% in 2015) (p <0.01).  Percent of parents considering baby teeth “less important” than adult teeth down from 21% in 2014 to 15% in 2015 (p <0.01). | **Behavioral outcomes**  Percent of children regularly drinking tap water from up 41% in 2014 to 63% in 2015 (p <0.01).  Reduced fruit juice consumption reported for young children from 66% in 2014 to 47% in 2015 (p <0.01).  **Retail outcomes**  Not reported | **Social norm and population health outcomes**  Not reported  **Policy outcomes**  Campaign contributed to new state rules prohibiting childcare centers from serving sugary beverages and capping 100% juice to twice weekly. |
| James et al. 2020^79^  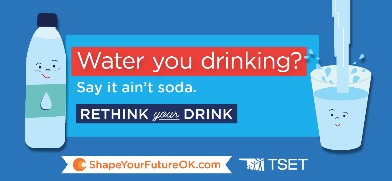 | ***Shape Your Future –***  ***Rethink Your Drink***  Oklahoma statewide  (2016-2017)  **Goal:** Educate people about the adverse health effects of sugary beverages and encourage them to limit sugary beverage intake. | **Population:** Parents and caregivers of children and adults with children living in the home.  **Strategies:** Combined print (posters), broadcast (i.e., radio, cable TV) and digital media.  **Theory or framework:**  Not reported | **Cognitive outcomes**  Among those with confirmed exposure, 76% reported sugary beverage consumption was linked to obesity, diabetes, and heart disease compared to 64% without confirmed exposure (p = 0.0045). | **Behavioral outcomes**  Sugary beverage intake decreased 18.6% (p = 0.0232) and heavy sugary beverage consumption (> 3/day) decreased 42.9% (p = 0.0083).  No differences in total sugary beverages by campaign exposure status.  **Retail outcomes**  Not reported | **Social norm, policy and population health outcomes**  Not reported |
| Maghrabi et al. 2021^80^  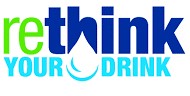 | ***Rethink Your Drink***  Rural City in SW Kentucky  4 weeks (2016)  **Goal:** Reduce sugary beverage consumption in the community to reduce obesity rates among adults. | **Population:** Adult volunteers (*n*=296) in *Rethink Your Drink* challenge.  **Strategies:** Campaign ran on local TV, radio and a website. Educational materials on beverage sugar content (categorized by red, yellow and green) and tracking tools provided to research partners in workplace human resource wellness initiatives. Volunteers filled in data sheets weekly.  **Theory or framework:**  Not reported | **Cognitive outcomes**  Not reported | **Behavioral outcomes**  Adults reported sugary beverage intake decreased weeks 1-4  (p < 0.001).  Average number of red (high sugar) beverages consumed weekly decreased (7 in week 1 to 3.2 drinks in week 4). Average number of green (no sugar) beverages increased (21 in week 1 to 27 drinks in week 4).  **Retail outcomes**  Not reported | **Social norm, policy and population health outcomes**  Not reported |
| Robles et al. 2015^81^  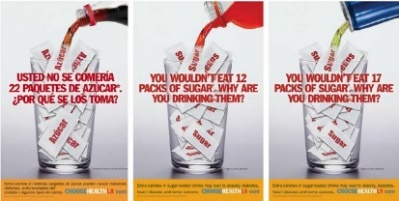 | ***Choose Health LA Sugar Pack***  Los Angeles County, CA (LAC) (2012)  **Goal:** Educate and encourage residents to reduce their sugary beverage consumption. | **Population:** LA County residents.  **Strategies:** English and Spanish ads in low-income areas with high obesity prevalence. (Metro) bus TVs, *Choose Health LA* website (www.choosehealthla.com)  and social media (i.e., Twitter, Facebook, YouTube). Ads were disseminated in the bus and railway systems.  **Theory or framework:**  Theory of planned behavior | **Cognitive outcomes**  Respondents who were exposed to campaign significantly more likely to perceive harm from soda or other sugary drinks (p<.01).  Different patterns related to intention emerged by population sub-groups (i.e., whether moderate or heavy sugary beverage consumers, education levels and/or age). | **Behavioral outcomes**  Not reported  **Retail outcomes**  Not reported | **Social norm, policy and population health outcomes**  Not reported |
| Samuels & Associates 2010^82^  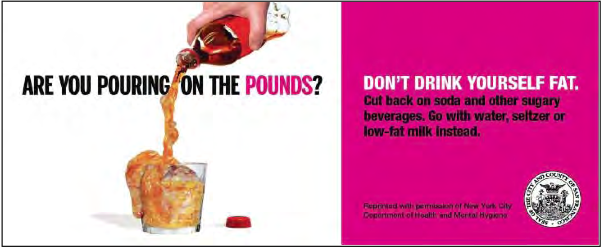  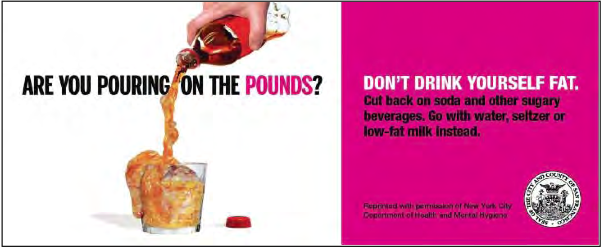 | ***Are You Pouring on the Pounds?***  San Francisco, CA (2010)  **Goal:** Motivate residents to eliminate or reduce sugary beverage consumption to prevent and reduce obesity. | **Population:** San Francisco residents.  **Strategies:** 400 ads were displayed on the interior of buses and 100 were posted on the exteriors during 2010 in Public Private Partnership.  (Blue Line Media donated 3 months of interior bus advertisement space to the San Francisco Department of Public Health that they purchased from the San Francisco Municipal Transit Agency. Additional advertising purchased by SFDPH to place the same posters on the exterior of buses for 6 weeks.)  No baseline. End-line Intercept Survey (n=318), two focus groups (n=17), and key informant interviews (n=7).  **Theory or framework:**  Not reported | **Cognitive outcomes:**  No differences in beliefs about sugary beverages and obesity/health outcomes between respondents who saw the campaign and those who did not post-campaign.  Of the respondents who had seen the campaign, only 13% reported that they had intended to decrease their consumption of sugary beverages.  One-third of focus group of participants had seen the campaign. Many participants reported that the campaign message was not clear. Despite general support for a sugary beverage tax, some participants expressed that a tax would not change behaviors. | **Behavioral outcomes**  ~60% of respondents reported infrequent intake of sugary beverages (across the three different data collection methods) post campaign.  No differences between survey respondents who saw the advertisement and those who did not in terms of sugary beverage consumption rates.  Some (2 out of 17 estimated) focus group participants said that the campaign made them think about sugary beverages and they then cut back on soda.  **Retail outcomes**  Not reported | **Social norm, policy and population health outcomes** Not reported |
| Schwartz et al. 2017^83^  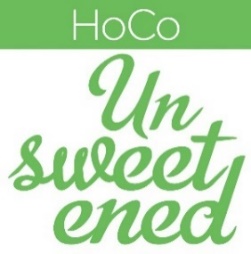 | ***Howard County Unsweetened***  Howard County, MD  (2013-2015)  **Goal:** Reduce sugary drinks intake of residents using policy, systems, and environmental strategies. | **Population:** Parents with children < 18 years.  **Strategies:** The community interventions included a multi-media campaign that disseminated ads through direct mail, outdoors, digital and social media platforms, cable television and broadcast media in child care centers, schools and health care settings. A 30-second PSA was aired called the Better Beverage Finder.org.  **Theory or framework:**  Socio-ecological model | **Cognitive outcomes**  Not reported | **Retail outcomes**  2012-2015: Regular soda sales in 15 Howard County (HC) stores decreased  (-19.7%) and sales were stable (0.8%) in 17 control stores. Fruit drink sales decreased (15.3%) in HC stores and was stable (-0.6%) in comparison stores. Sales of 100% juice decreased more in HC (-15.0%) than comparison (-2.1%) stores Sales of sports drinks and diet soda decreased in both communities, but the decreases were not significantly different between groups. | **Social norm, policy and population health outcomes**  Not reported |
| **5. Media advocacy or counter-marketing campaigns** (*n* = 1 evaluation was identified for The Bigger Picture campaign). | | | | | |
| Schillinger et al. 2018^86^  *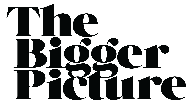* | ***The Bigger Picture***  San Francisco, CA (2013)  **Goal:** Encourage youth to reflect on how sugary beverages influence larger social, structural and environmental forces that shape behaviors and type 2 diabetes risk in an in-depth qualitative review. | **Population:** Low-income, ethnically and racially diverse youth (n= 13).  **Strategies:** Campaign website, PSAs disseminated via live high school assemblies, workshops and social media.  Youth filled out individual questionnaire and participated in a reflexive focus group.  **Theory or framework:**  Not reported | **Cognitive outcomes**  Less than half (43%) of youth recognized key PSA messages. More in the focus groups (75%) identified type 2 diabetes in a public health literacy frame than (54%) for individual questionnaires. Four prominent themes emerged: Individual (choice to be healthy), environment (structural forces in the built environment), financial (in context of poverty and food insecurity, eating habits and choices are limited), institutional (deceptive marketing). | **Behavioral outcomes**  Not reported  **Retail outcomes**  Not reported | **Social norm, policy and population health outcomes**  Not reported |
| **6. Public policy or political media campaigns** (*n* = 0 evaluations were identified for sugary beverage tax campaigns to discourage sugary beverages). | | | | | |

Abbreviations and Acronyms: Balance Calories Initiative (BCI); California (CA); Kentucky (KY); Los Angeles (LA); low-fat milk (LFM); Maryland (MD); New Jersey (NJ); New York (NY); New York City (NYC); non-fat milk (NFM); Oklahoma (OK); Oregon (OR); Pennsylvania (PA); public service announcement (PSA); San Francisco Department of Public Health (SFDPH); Santa Paula (SP); television (TV), Virginia (VA), whole milk (WM); West Virginia (WV).

* John et al. 2019 summarized relevant evidence from two earlier evaluations of the 1% Low-Fat Milk has Perks! Campaign implemented in Tulsa, OK and statewide. Therefore, Finnel et al. 2017 and Finnel and John 2018 were not included in the table.

**References** *(These numbers correspond with the published manuscript)*

51. Bogart LM, Castro G, Cohen DA. A qualitative exploration of parents’, youths’ and food establishment managers’ perceptions of beverage industry self-regulation for obesity prevention. *Public Health Nutr.* 2019;22(5):805–813. <https://doi.org/10.1017/S1368980018003865>.

52. Cohen DA, Bogart L, Castro G, Rossi AD, Williamson S, Han B. Beverage marketing in retail outlets and The Balance Calories Initiative. *Prev Med.* 2018;115:1–7. <https://doi.org/10.1016/j.ypmed.2018.07.014>.

55. Hinckle AJ, Mistry R, McCarthy WJ, Yancey AK. Adapting a 1% or less milk campaign for a Hispanic/Latino population: the Adelante Con Leche Semi-descremada 1% experience. *Am J Health Promot.* 2008;23(2):108–111. <http://dx.doi.org/10.4278/ajhp.07080780>.

56. John R, Finnell KJ, Scott-Kaliki MS, DeBerry S.M. A case study of two successful social marketing interventions to promote 1% low-fat milk consumption. *Soc Market Quarter.* 2019;25(2):137–159. [https://doi.org/10.1177/1524500418824292](https://doi.org/10.1177%2F1524500418824292).

57. Maddock J, Maglione C, Barnett JD, Cabot C, Jackson S, Reger-Nash B. Statewide implementation of the 1% or Less Campaign. *Health Educ Behav.* 2007;34(6):953–963. <http://dx.doi.org/10.1177/1090198106290621>.

58. Reger B, Wootan MG, Booth-Butterfield S, Smith H. 1% or less: a community-based nutrition campaign. *Public Health Rep.* 1998;113(5):410–419. <https://pubmed.ncbi.nlm.nih.gov/9769765>.

59. Reger B, Wootan MG, Booth-Butterfield S. Using mass media to promote healthy eating: a community-based demonstration project. *Prev Med.* 1999;29(5):414–421. <https://doi.org/10.1006/pmed.1998.0570>.

60. Reger B, Wootan MG, Booth-Butterfield S. A comparison of different approaches to promote community-wide dietary change. *Am J Prev Med*. 2000;18(4):271–275. <https://doi.org/10.1016/S0749-3797(00)00118-5>.

61. Wechsler H, Wernick SM. A social marketing campaign to promote low-fat milk consumption in an inner-city Latino community*. Public Health Rep.* 1992;107(2):202–207. <https://www.ncbi.nlm.nih.gov/pmc/articles/PMC1403632/>.

62. Wootan MG, Reger-Nash B, Booth-Butterfield S, Cooper L. The cost-effectiveness of 1% or less media campaigns promoting low-fat milk consumption. *Prev Chronic Dis*. 2005;2(4):A05. <http://www.ncbi.nlm.nih.gov/pmc/articles/PMC1435702/>.

63. Bonnevie E, Morales O, Rosenberg SD, Goldbarg J, Silver M, Wartella E, Smyser J. Evaluation of a campaign to reduce consumption of sugar-sweetened beverages in New Jersey. *Prev Med.* 2020;136:106062. <https://doi.org/10.1016/j.ypmed.2020.106062>.

64. Farley T, Halper HS, Carlin AM, Emmerson KM, Foster KN, Fertig AR. Mass media campaign to reduce consumption of sugar-sweetened beverages in a rural area of the United States. *Am J Public Health*. 2017;107(6):989–995. [https://doi.org/10.2105/AJPH.2017.303750](https://ajph.aphapublications.org/doi/abs/10.2105/AJPH.2017.303750).

72. Barragan NC, Noller AJ, Robles B et al. The "sugar pack" health marketing campaign in Los Angeles County, 2011-2012. *Health Promot Pract*. 2014;15(2):208–216. [https://doi.org/10.1177/1524839913507280](https://doi.org/10.1177%2F1524839913507280).

73. Bleakly A, Jordan, A, Mallya G, Hennessy M, Piotrowski JT. Do you know what your kids are drinking? Evaluation of a media campaign to reduce consumption of sugar-sweetened beverages. *Am J Health Promot*. 2018;32(6):1409–1416. [https://doi.org/10.1177/0890117117721320](https://doi.org/10.1177%2F0890117117721320).

74. Boehm R, Cooksey Stowers K, Schneider GE et al. Race, ethnicity, and neighborhood food environment are associated with adolescent sugary drink consumption during a 5-year community campaign. *J Racial Ethnic Health Disparities.* August 2021 (early release). <https://doi.org/10.1007/s40615-021-01074-9>.

75. Boles M, Adams A, Gredler A, Manhas S. Ability of a mass media campaign to influence knowledge, attitudes, and behaviors about sugary drinks and obesity. *Prev Med*. 2014;67(Suppl 1):S40–S45. https://doi.org/10.1016/j.ypmed.2014.07.023.

76. Caldwell JI, Robles B, Tyree R, Fraser RW, Dumke KA, Kuo T. Does exposure to the Choose Water campaign increase parental intentions to promote more water and less sugar-sweetened beverage consumption? *Am J Health Promot*. 2020;34(5):555–558. <https://doi.org/10.1177%2F0890117120908785>.

77. Hartigan P, Patton-Ku D, Fidler, C, Boutelle KN. Rethink Your Drink. *Health Promot Pract*. 2017;18(2):238–244. [https://doi.org/10.1177/1524839915625215](https://doi.org/10.1177%2F1524839915625215).

78. Hornsby WC, Bailey W, Braun PA, Weiss K, Heichelbech J. Busting the baby teeth myth and increasing children's consumption of tap water: building public will for children's oral health in Colorado. *Front Public Health.* 2017;5*:*238. <https://doi.org/10.3389/fpubh.2017.00238>.

79. James SA, White AH, Paulson SW, Beebe LA. Factors associated with sugar-sweetened beverage consumption in adults with children in the home after a statewide health communications program. *BMC Nutr*. 2020;6:23. <https://doi.org/10.1186/s40795-020-00349-4>.

80. Maghrabi P, Terry M. Effectiveness of a community-based health promotion “Rethink Your Drink” on reducing sugary beverage consumption: a case study.  *J Exercise Nutr.* 2021;1(5):1–4. <https://www.journalofexerciseandnutrition.com/index.php/JEN/article/view/24>.

81. Robles B, Blitstein JL, Lieberman AJ, Barragan NC, Gase LN, Kuo T. The relationship between amount of soda consumed and intention to reduce soda consumption among adults exposed to the Choose Health LA 'Sugar Pack' health marketing campaign*. Public Health Nutr.* 2015;18(14):2582–2591. <https://doi.org/10.1017/S1368980014003097>.

82. Samuels & Associates. *Evaluation of San Francisco’s Social Marketing Campaign “Pouring on the Pounds.”* California Obesity Prevention Program, 2010. Available online: <https://www.iccp-portal.org/sites/default/files/multimediaresources/San%20Francisco_Pouring_on_the_Pounds_Report.pdf> (accessed on 1 October 2021).

83. Schwartz MB, Schneider GE, Choi YY et al. Association of a community campaign for better beverage choices with beverage purchases from supermarkets. *JAMA Intern Med*. 2017;177(5):666–674. <https://doi.org/10.1001/jamainternmed.2016.9650>.

86. Schillinger D, Tran J, Fine S. Do low income youth of color see "*The Bigger Picture*" when discussing type 2 diabetes: a qualitative evaluation of a public health literacy campaign. *Int J Environ Res Public Health.* 2018;15(5):840. <http://dx.doi.org/10.3390/ijerph15050840>.
