# Supplementary material for: How have media campaigns been used to promote and discourage healthy and unhealthy beverages in the United States? A systematic scoping review to inform future research to reduce sugary beverage health risks
Source: Obes Rev. 2022 Feb 9;23(5):e13425. doi: 10.1111/obr.13425 (PMC9286342; doi:10.1111/obr.13425)
Supplement: Supplementary file 3 — Table S3: Fair use evaluation documentation for the images used in Figures 3 and 4 and Supplemental Table 2 to illustrate beverage campaigns used to promote or discourage sugary beverages and encourage water, milk or 100% juice to Americans, 1886–2021. [file OBR-23-0-s001.pdf]

Fair Use Evaluation Documentation

Compiled using the **Fair Use Evaluator** [cc] 2008 Michael Brewer & the Office for Information Technology Policy, http://librarycopyright.net/fairuse/

**Name:**	Katherine Consavage Stanley, MS
**Job Title:**	PhD Student and Graduate Teaching Assistant
**Institution:**	Virginia Polytechnic Institute and State University
**Title of Work Used:**	Trademark or copyright images from U.S. beverage campaigns
**Copyright Holder:**	Global, national, regional and local companies and organizations, including multinational beverage companies, U.S.-based nonprofits and advocacy groups, and state and local governments, among others
**Publication Status:**	Published
**Publisher:**	Organizations who have funded or helped implement U.S. beverage campaigns
**Place of Publication:**	Internet and organization websites
**Publication Year:**	Varies based on campaign date
**Description of Work:**	The images will be included as part of two figures in a manuscript titled "A Systematic Scoping Review of Media Campaigns Used to Promote or Discourage Beverages in the United States to Reduce Sugary Beverage Health Risks for Americans"
**Date of Evaluation:**	October 5, 2021
**Date of Intended Use:**	October 5, 2021

Describe the Purpose and Character of Your Intended Use:

[+] Use is for "criticism, comment, news reporting, teaching, (including multiple print copies for classroom use), scholarship or research". The images used are a component of a systematic scoping review and analysis of U.S. beverage media campaigns conducted as part of a research study. The use is therefore solely for research.
[+] Use is transformative, i.e. it uses the existing work in a new way (creates an index to the work) or for a new purpose (parody, pastiche, instructional materials, etc.) and is socially beneficial. The study is innovative in its use of a media campaign typology to analyze and describe the landscape of U.S. beverage media campaigns. The findings of this study are used to suggest future actions and research needed to inform a social change movement to promote healthy hydration behaviors, with the aim of contributing to behavior change efforts to reduce sugary beverage health risks for Americans.
[+] Use is one-time, or is only occasional or spontaneous. The images will only be used in the published manuscript and for educational promotion of the manuscript on social media once published.
[+] Use is clearly defined, restricted in scope (limited duration, not iterative, restricted access, etc.), and not-for-profit. The use of these images is only in relation to specific U.S. beverage media campaigns identified as part of the systematic scoping review conducted for this study and is meant for illustrative purposes. The images will not be used

beyond the manuscript and educational promotion of the manuscript. The authors and published will not receive any monetary contribution for the use of these images.

Fair | Unfair

☑ ☐ ☐ ☐ ☐ ☐ ☐

Describe the **<u>Nature</u>** **of Your Intended Use of the Copyrighted Work:**

[+] Work to be used has been previously PUBLISHED. The images being used in the manuscript have all been published elsewhere on the Internet and/or in print media, and they will be used solely asa companion to the text in the manuscript, in order to visually depict the messaging and images that accompanied select U.S. beverage campaigns.

Fair | Unfair

☐ ☑ ☐ ☐ ☐ ☐ ☐

Describe the **<u>Amount</u>** **of Your Intended Use in Relation to the Copyrighted Work as a Whole:**

[+] The portion used is not the "heart" of the work (the portion considered most central to the work as a whole). The campaigns identified through the study's systematic scoping review, and their organization based on a media campaign typology, represents the central component of this manuscript. The copyrighted images will solely act as visual support for the campaigns described within the manuscript.
[+] Only the amount required to achieve the stated, socially-beneficial purpose or objective will be used (be that educational, artistic, scholarly, journalistic, etc.). The use of copyrighted images in the manuscript will be limited to only the quantity necessary to visually convey the findings of the study with regard to the landscape of U.S. beverage media campaigns and their categorization based on the media campaign typology. An educational promotion of the manuscript, once published, will only utilize the same images and figures included in the manuscript.

Fair | Unfair

☐ ☑ ☐ ☐ ☐ ☐ ☐

Describe the Effect of Your Intended Use on the Potential Market or Value of the Copyrighted Work:

[+] Use of the work minimizes the potential for unauthorized use that could impact its value (i.e. steps are taken to ensure the content is not used outside of the stated purpose or audience). The copyrighted/trademarked images will only be used as part of the manuscript, which will be published in a peer-reviewed journal that's audience is academics and health professionals. The images will only be used for the purposes outlined within this fair use evaluation.
[+] Proper attribution will be given with the intended use

Fair — Unfair

[ ] [ ] [x] [ ] [ ] [ ] [ ]

The Average "Fairness Level," Based on Your Rating of Each of the 4 Factors, Is:

[see tool disclaimer for important clarifying information]:

Fair — Unfair

[ ] [x] [ ] [ ] [ ] [ ] [ ]

Based on the information and justification I have provided above, I, Katherine Consavage Stanley, MS, am asserting this use is **FAIR** under Section 107 of the U.S. Copyright Code.

Signature: Katherine Consavage Stanley

Date of Signature: 10/5/2021

***Disclaimer:** *This document is intended to help you* collect, organize & archive the information you might need to support your fair use evaluation. *It is not a source of legal advice or assistance. The results are only as good as the input you have provided by are intended to suggest next steps, and not to provide a final judgment. It is recommended that* you share this evaluation with a copyright specialist before proceeding with your intended use.
